# Supplementary material for: Multilabel prediction of virus target proteins via multimodal graph representation learning
Source: PLoS Comput Biol. 2026 May 26;22(5):e1014320. doi: 10.1371/journal.pcbi.1014320 (PMC13229406; doi:10.1371/journal.pcbi.1014320)
Supplement: S1 File — This document contains the following sections: S1.1 Analysis of model specificity in predicting host-virus relationships. S1.2 Analysis of model specificity in predicting VTPs within shared pathways. S1.3 Impact of sample similarity on prediction performance. S1.4 Network dependency analysis of MultiVTP. S1.5 Effect of PPI data quality on model performance. S1.6 Evaluation of simplified MultiVTP architectures. S1.7 Interpretability analysis via linking attention weights. S1.8 Comparison of MultiVTP with virus-host PPI prediction methods. S1.9 Robustness assessment under extreme data scarcity and unseen viruses. S1.10 Generalization to novel viruses with limited samples. S1.11 Comparative analysis of HIV-1 VTP candidates and HDFs. S1.12 Model re-evaluation using an updated dataset. S1.13 Comparison of different negative sampling strategies. S1.14 MultiVTP extension for directional interaction prediction. S1.15 Sensitivity analysis of VTP count thresholds. S1.16 Reasons for various decisions in building our model. (PDF) [file pcbi.1014320.s001.pdf]

### S1.1 Analysis of model specificity in predicting host-virus relationships

To determine whether our model predicts genuine host-virus interactions or merely captures host susceptibility, we categorized VTPs into different types. First, VTPs were divided into multiple virus target proteins (MVTPs) and single virus target proteins (SVTPs) according to the number of targeting viruses. Second, according to Chai et al., VTPs were grouped into forward (pro-viral) and backward (anti-viral) types based on interaction directionality [1]. Forward interactions mainly promote viral replication and are largely mediated by viral proteins, whereas backward interactions typically involve host immune-related proteins and are primarily mediated by host proteins. Because we only collected directionality annotations for HIV-1 VTPs, we used this virus as a case study. Analysis of prediction scores from 5-fold cross-validation showed that non-VTPs and VTPs targeted by other viruses exhibited relatively low scores, whereas HIV-associated MVTPs and SVTPs achieved higher scores. Notably, HIV-1 SVTPs possessed higher scores than MVTPs targeted by multiple distinct viruses (including HIV-1). This implies that our model not only learns a common host susceptibility signature but also effectively identifies HIV-specific biological characteristics, thereby distinguishing true virus-specific host-virus interactions. For forward interactions mediated by viral proteins, the median prediction score was similar to that of non-VTPs, and only the upper quartile suggested that the model could capture relevant interaction patterns. In contrast, for backward interactions mediated by host proteins, their prediction scores were significantly higher than those of other categories (Figure A). This confirms that our model excels at predicting host protein-mediated interactions but has limitations in predicting those mediated by viral proteins.

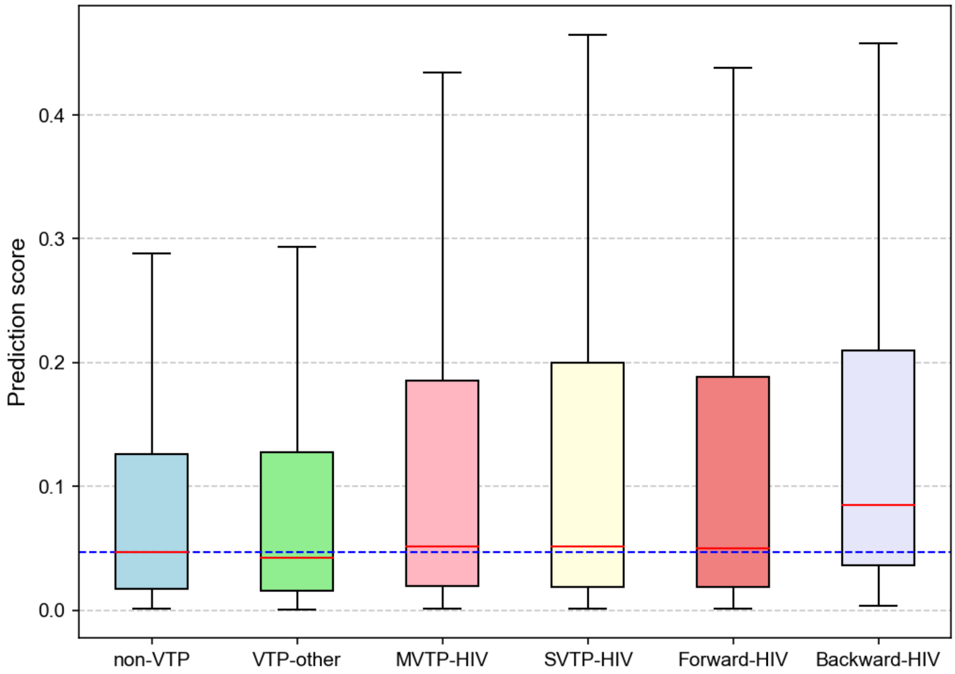


Figure A. Distribution of prediction scores for HIV-1 target proteins.

### S1.2 Analysis of model specificity in predicting VTPs within shared pathways

To investigate whether our model could identify the target proteins of different viruses in the same pathway, we analyzed VTPs and non-VTPs within the pathways from the test set. For the NF-κB pathway, the analysis showed that the predicted scores for VTPs of HIV-1, HHV-4, and HPV16 were higher than those of non-VTPs, while the results of HHV-1 and HCV-1b showed the opposite trend (Figure BA). This discrepancy may stem from the limited number of proteins covered by the NF-κB pathway in the test set (only 12 proteins), resulting in insufficient statistical power. We further extended our analysis to the cytokine-cytokine receptor interaction pathway (41 proteins), the immune pathway containing the largest number of proteins in the test set (Figure BB). Due to the scarcity of known VTPs in this pathway, the results remain inconclusive. To provide a more robust and convincing analysis, we retrained our model after excluding all NF-κB pathway-related proteins from the training set and re-evaluated the model on these proteins along with those from the test set (a total of 60 proteins). The result showed that VTPs had higher prediction score than non-VTPs, demonstrating that our model could effectively predict the VTPs of different viruses in the same pathway (Figure BC).

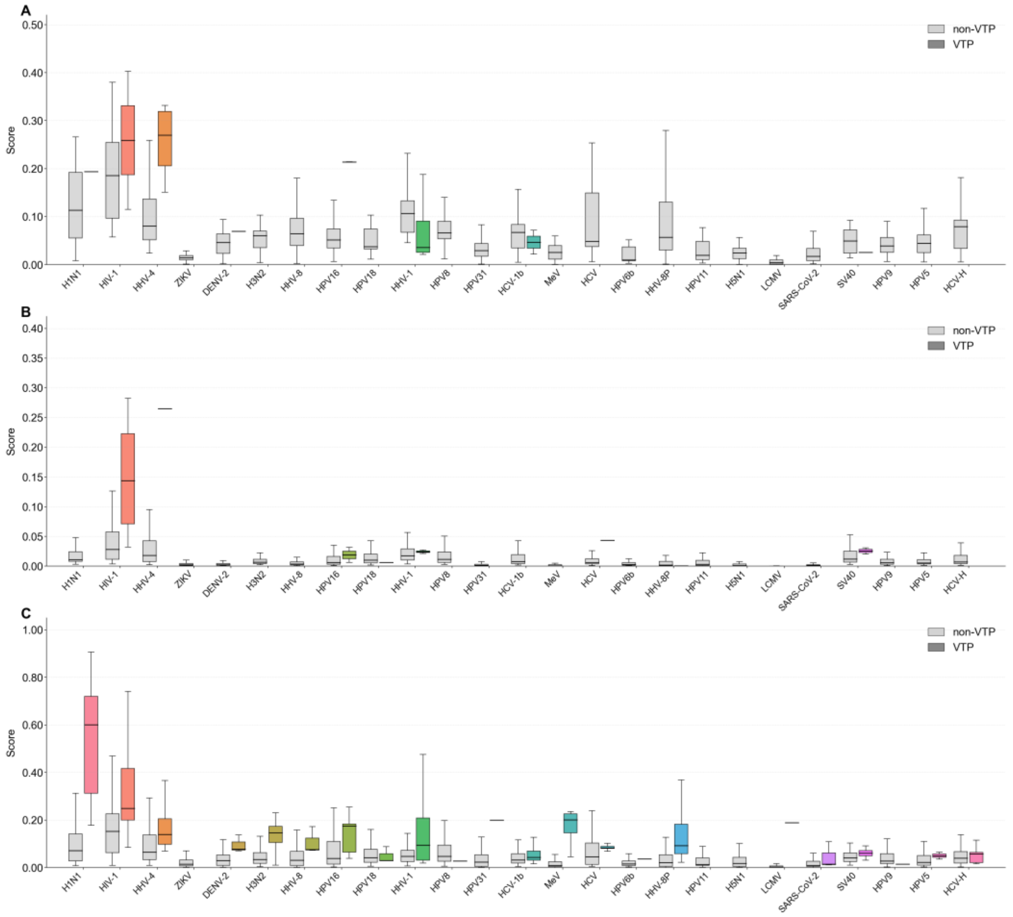


Figure B. Comparison of prediction scores for VTPs and non-VTPs in the same pathway. (A) Prediction scores of proteins involved in the NF-κB pathway. (B) Prediction scores of proteins involved in the cytokine-cytokine receptor interaction pathway. (C) Prediction scores of proteins involved in the NF-κB pathway using the retrained model.

### S1.3 Impact of sample similarity on prediction performance

To analyze the impact of potential similarities on the performance, we quantified the similarity between the training and test sets across three dimensions: protein sequence, functional annotation, and network topology.

For sequence homology, we employed BLAST to conduct sequence similarity analysis for each fold during cross-validation. Specifically, we compared each protein in the validation set against all proteins in the corresponding training set, and used the highest sequence identity as the homologous measure. As a result, the distributions of sequence similarity were consistent across all folds (Figure CA). Subsequently, we evaluated the performance on validation samples based on their similarity relative to training samples. This evaluation revealed that our model's effectiveness did not degrade as sequence identity decreased. Even in the lowest sequence identity range (0, 20), the MCC, AUPR, and F1-score all remained above 0.4 (Figure CB). These results implied that the model's predictive ability was not dependent on homologous information, and that no overfitting risk existed at the sequence level.

For functional annotation, we used GOSemSim to quantify functional similarity. The highest functional similarity score was assigned to each protein in the validation set. The results showed that the distribution of functional similarity was consistent, with values centered at approximately 0.3 (Figure CC). Although the model's performance fluctuated with decreasing functional similarity, the MCC and F1-score remained relatively stable even in the lowest functional similarity range (0, 0.3) (Figure CD). This indicates that the model maintained effective predictive performance for samples with functional annotations clearly distinct from those in the training set, demonstrating that it truly learned the biological principles underlying VTPs.

For network proximity, topological similarity was quantified using the maximum neighborhood overlap rate between each validation protein and all training proteins. The low neighborhood overlap (around 0.1) indicates limited topological resemblance between the training and test sets (Figure CE). Threshold-based evaluations revealed that the model maintained strong performance for proteins with an overlap rate above 0.2, while performance dropped noticeably for those below 0.1 (Figure CF). The inferior performance may be attributed to their inherent biological characteristics. In the highly connected host PPI network, these proteins are typically peripheral or isolated nodes that rarely participate in core functional pathways. Viral targeting of such proteins may be highly specific yet idiosyncratic, thus lacking generalizable patterns. Besides, these proteins often have sparse functional and interaction annotations in public databases, further increasing prediction difficulty.

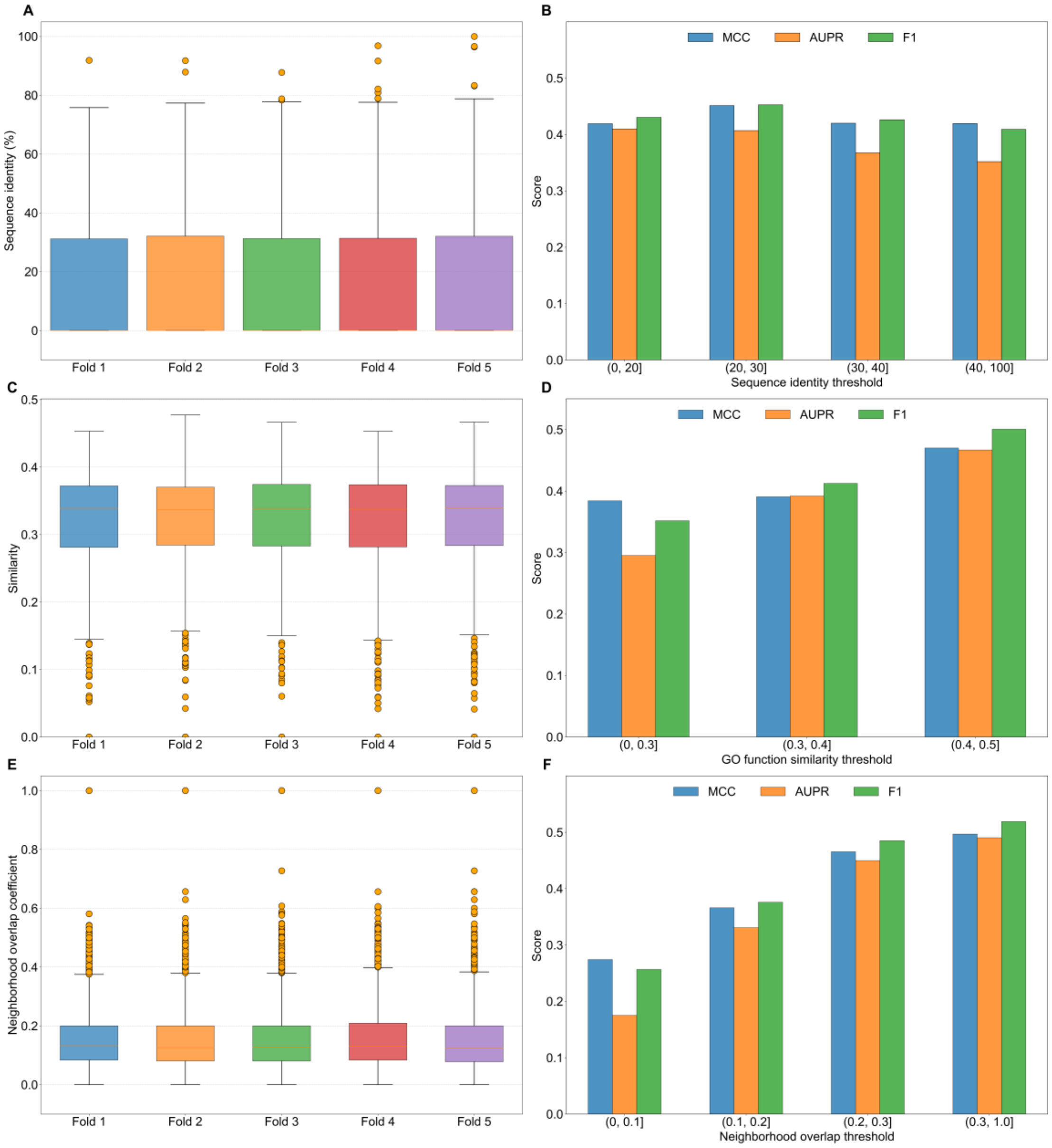


Figure C. Cross-fold similarity and model performance on the $D_{virus}$ dataset. (A) Sequence identity distribution across all folds. (B) Performance on cross-validation sets under different sequence identity thresholds. (C) Functional similarity distribution across all folds. (D) Performance on cross-validation sets under different functional similarity thresholds. (E) Topological similarity distribution across all folds. (F) Performance on cross-validation sets under different topological similarity thresholds.

### S1.4 Network dependency analysis of MultiVTP

To determine whether the model detects merely high-centrality proteins or truly virus targets, we re-evaluated its performance under the degree-controlled settings. Specifically, we divided the proteins into 10 bins based on their degree measures in the PPI network and assessed the performance using cross-validation. Although performance decreased across the (10, 70) intervals, our model still attained a notable AUPR of 0.287 for the lowest-degree interval, suggesting that VTP prediction does not solely rely on centrality signals (Figure DA). Furthermore, we conducted two additional ablation studies to ensure the model is not merely detecting topological features and to prevent inflated performance due to network proximity. First, we excluded all network-related components, including traditional topological properties, global/local topological attributes, and subgraphs. As a result, the model still retained robust performance, suggesting that its utility is not solely driven by these topological features. Second, we masked edges between the training and test samples to prevent information leakage and re-extracted all network topological features and subgraphs (Figure DB). The results showed that the model maintained stable performance, confirming that the predictive power of the model is not due to information leakage from neighboring proteins.

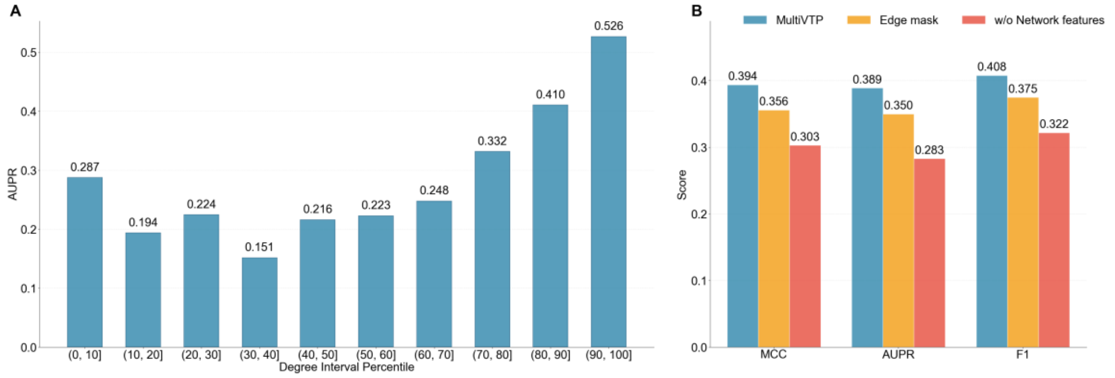


Figure D. Validation of MultiVTP's network dependency. (A) Performance evaluation on proteins with different degree measures on the $D_{virus}$ dataset. (B) Ablation studies of network features on the $D_{virus}$ dataset.

### S1.5 Effect of PPI data quality on model performance

To evaluate the impact of low-confidence data in the HIPPIE database, we filtered out PPIs with scores below 0.5 and re-evaluated our model using cross-validation. The results suggested that the complete network achieved better performance, demonstrating that low-confidence interactions also contain useful information for VTP prediction (Figure E).

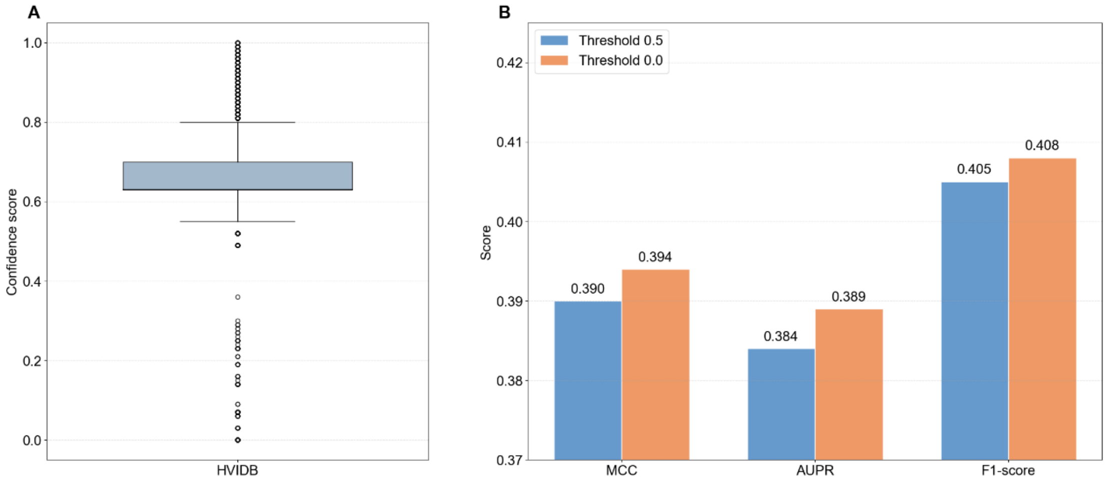


Figure E. Evaluations of interaction confidence in HVIDB. (A) Distribution of confidence scores for protein interactions. (B) Performance comparison between interaction networks with different confidence thresholds on the $D_{virus}$ dataset.

### S1.6 Evaluation of simplified MultiVTP architectures

To evaluate whether simpler architectures can achieve comparable performance, we conducted three additional ablation studies using only node2vec and ESM2 embeddings as features: (1) replacing the PLE module with a shared MLP head; (2) replacing the Graphormer with a 2-layer GCN; and (3) applying both modifications simultaneously. The results demonstrated that each component in the original model contributes to the overall performance, and a substantial performance drop occurs when these key components are replaced (Figure F).

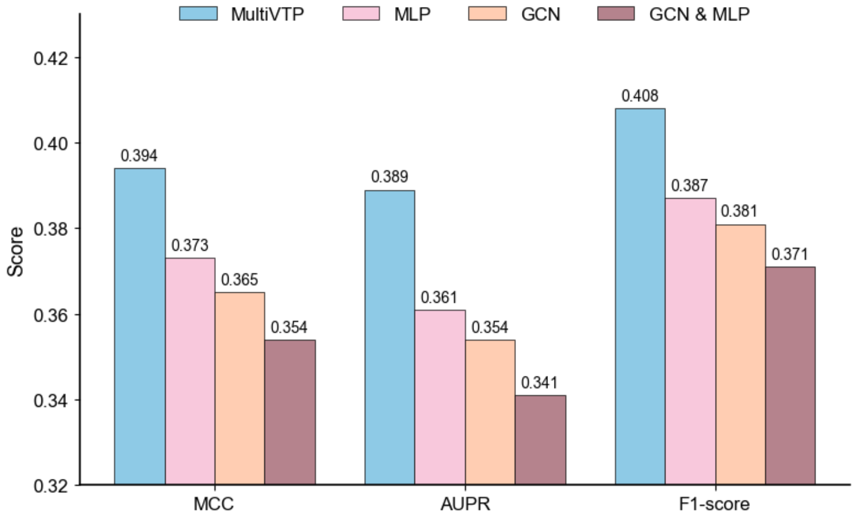


Figure F. Performance comparison between MultiVTP and simpler architectures on the $D_{virus}$ dataset.

### S1.7 Interpretability analysis via linking attention weights

In the main text, we found that our model assigned higher attention scores to VTPs. These proteins were enriched in biological processes closely associated with viral infection, including protein transport, apoptosis, transcriptional regulation, and antiviral defense. To further associate attention weights with interpretable biological functions, we extracted functional regulatory relationships between host proteins from the Signor database and classified them into activation and inhibition interactions [2]. By comparing attention scores, we observed higher values for protein pairs involved in such regulatory relationships, suggesting that our model could effectively capture key regulatory mechanisms (Figure GA). The heightened attention toward VTPs and regulatory interactions may reflect the molecular perturbations induced during viral infection. For instance, in the subgraph centered on BCL2A1, high attention scores were detected among BCL2A1, BAX, and BRD4. Previous studies have verified that the viral protein E2 binds to BRD4 during HPV infection, promoting the activation of BCL2A1, which in turn enhances inhibition of the downstream protein BAX. This cascade suppresses apoptosis in infected cells and creates a favorable environment for viral replication [3]. Another high-attention protein pair, KLHDC10 and RBX1, also represents a strong regulatory interaction. Upon recognition of viral proteins by KLHDC10, this protein recruits RBX1 to amplify the apoptotic signal, thereby eliminating infected cells and degrading viral replication factors (Figure GB) [4].

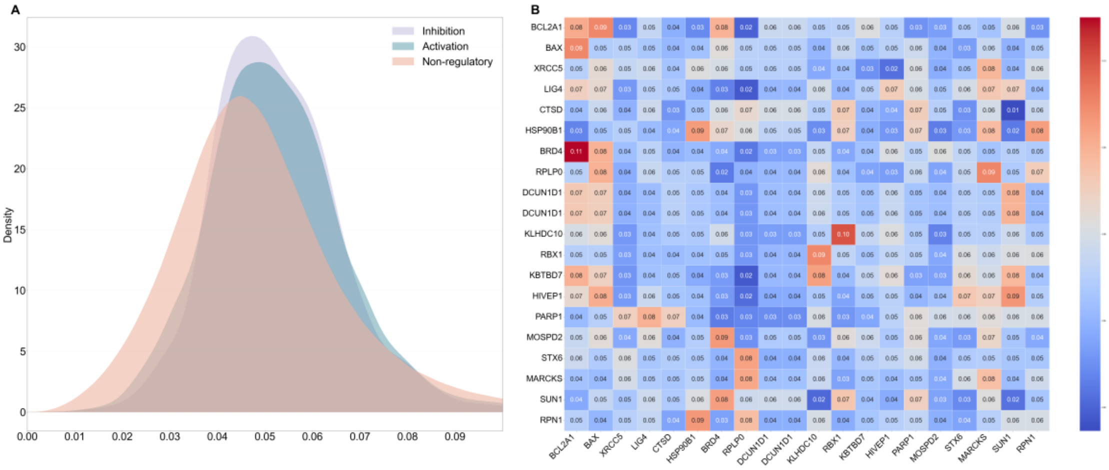


Figure G. Linking attention score analysis on the $D_{virus}$ dataset. (A) Density distribution of attention values of protein pairs across regulatory interaction types. (B) Attention scores within BCL2A1-centered subgraph.

### S1.8 Comparison of MultiVTP with virus-host PPI prediction methods

We selected two representative deep learning models as supplementary baselines, namely D-SCRIPT and HBFormer. D-SCRIPT is a sequence-based PPI prediction model with strong generalization in cross-species interaction prediction, whereas HBFormer uses a hybrid attention transformer combined with multimodal features for host-virus PPI prediction [5, 6]. Because these models were originally designed for binary PPI predictions, we adjusted the prediction strategy to ensure a fair comparison. Specifically, each host protein was paired with all viral proteins in the HVIDB database, and the highest score among all PPI predictions was used as the prediction score for the host protein. Considering the high computational cost of this pairing strategy, D-SCRIPT was evaluated on all 25 viruses due to its higher efficiency, whereas HBFormer was only tested on the five viruses with the highest VTP counts owing to its lower efficiency. The results suggested that MultiVTP remarkably outperformed the baseline models in terms of AUPR across all viruses (Figure H). This may be because selecting the highest PPI score leads the model to more easily classify host proteins as false positives. Therefore, traditional binary PPI classification methods are not suitable for VTP prediction.

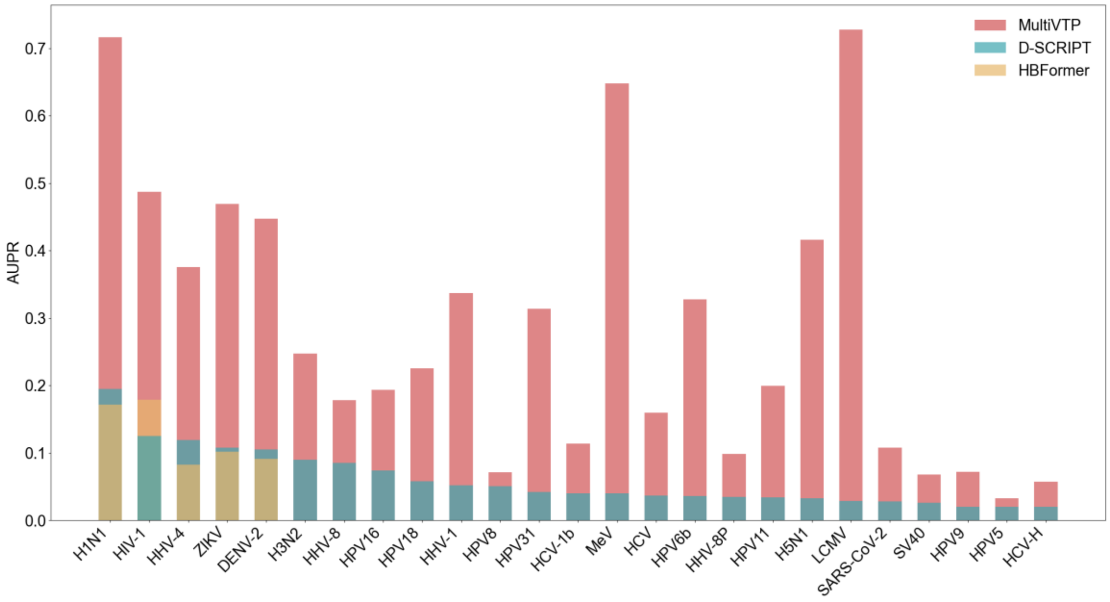


Figure H. Performance comparison of MultiVTP with virus-host protein interaction prediction methods.

### S1.9 Robustness assessment under extreme data scarcity and unseen viruses

To assess the model's robustness under more stringent low-data conditions, we conducted two experiments as requested. First, we simulated scenarios with extremely scarce labeled data by progressively removing VTPs for each virus in the training set at a high ratio. Notably, when 99% of VTPs were removed, even for the virus with the largest number of targets (H1N1), fewer than 20 VTPs remained available for training. The model was then evaluated on the test set. All experiments were repeated five times to reduce fluctuations caused by random sampling under limited data. The results showed that our model maintained stable AUPR values even after removing 90% of known VTPs, while performance dropped sharply when 99% of VTPs were eliminated (Figure IA). These results confirmed that the model remains effective under scarce labeled data conditions. Second, we performed leave-one-virus-out validation on five randomly selected viruses. Specifically, the model was pre-trained on a training set that excluded VTPs of the target virus. Because the pre-trained model lacked an output channel for the new virus, we introduced a virus-specific expert and prediction head, which were then fine-tuned using only 20 targets from that virus. The model was subsequently evaluated on the test set, with all experiments repeated five times. Compared with the original training scheme, this strategy yielded reduced but still effective performance across the five viruses, verifying the model's generalization ability to unseen viruses (Figure IB).

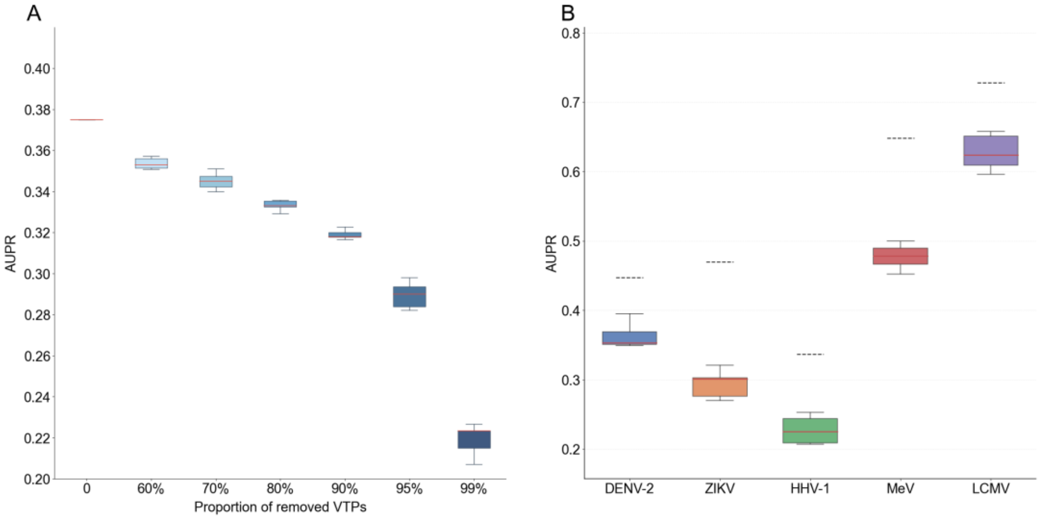


Figure I. Robustness of MultiVTP with limited training data on the $D_{virus}$ dataset. (A) Performance of MultiVTP after removing different fractions of training VTPs. (B) Performance of leave-one-virus-out validation.

## S1.10 Generalization to novel viruses with limited samples

To further address the issue of viruses with limited available data, we constructed a more challenging few-shot dataset consisting of 15 novel viruses (each with 5-17 VTPs), with none of these samples appearing in the training set (Figure JA). Under this setting, models trained from scratch suffered from severe performance collapse (AUPR < 0.1). In contrast, our fine-tuning strategy achieved clearly better performance, with AUPR values ranging from 0.1 to 0.7 for most viruses. Relatively poor performance was observed for the remaining viruses (e.g., HPV3, HPV16, H7N7, and H9N2), likely because some of their targets are SVTPs that lack cross-virus generalizable patterns, which remains a challenge for the current model (Figure JB). Collectively, our model is suitable for understudied or newly emerging viruses.

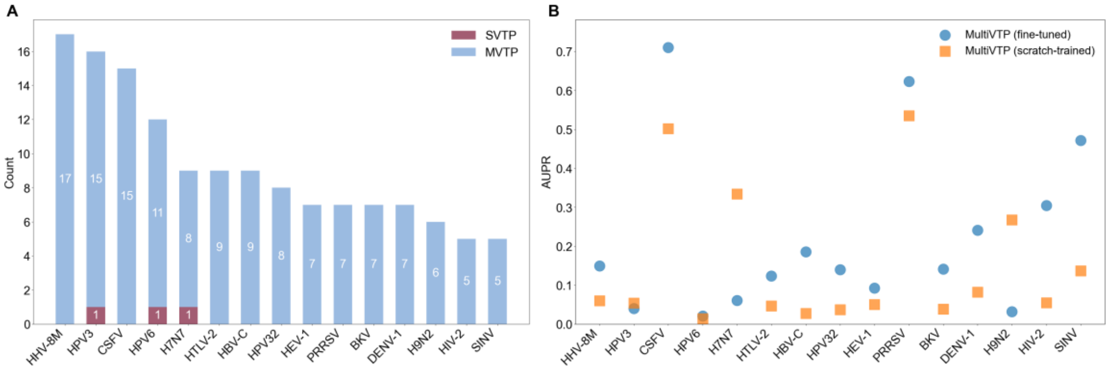


Figure J. Evaluation of MultiVTP on understudied viruses. (A) Distribution of VTP counts across viruses in the few-shot dataset. (B) Performance of our model on understudied viruses.

## S1.11 Comparative analysis of HIV-1 VTP candidates and HDFs

After collecting the host dependency factors (HDFs) of HIV-1, we performed functional enrichment analysis using the DAVID database. Figure K shows the overlap of enriched terms between HDFs and VTP candidates, suggesting that these two types of host proteins are highly associated during the infection process (Figure KA). We then ranked the biological process terms of HDFs based on their enrichment significance. Among the top 20 terms, HDFs were associated with core infection-related processes, such as defense response to virus, immune and inflammatory regulation, regulation of apoptosis, and regulation of gene expression. Notably, VTP candidates were also enriched in 15 out of these 20 key terms (Figure KB). Moreover, we calculated the recovery rate (overlap ratio) of VTP candidates for the top k terms. For terms within the top 50, the recovery rate of VTP candidates exceeded 70%. Even for the top 100 terms, the recovery rate remained stable at approximately 60%, and it only dropped below 50% for terms beyond the top 200 (Figure KC). These indicated that the predicted VTPs were biologically reliable and closely related to known infectious functions. Subsequently, we focused on the term ‘regulation of autophagy’, which was enriched in both VTP candidates and HDFs. Among the 12 candidates, 8 corresponded to known VTPs, and an additional 3 were supported by literature mining. Notably, USP10 (UniProt ID: Q14694) and MTOR (UniProt ID: P42345) were identified as HDFs [7, 8]. Moreover, MTOR has been validated as a key regulator of autophagy during ZIKV and HCMV infections, implying that it may exert a similar function during HIV-1 infection (Figure KD) [9].

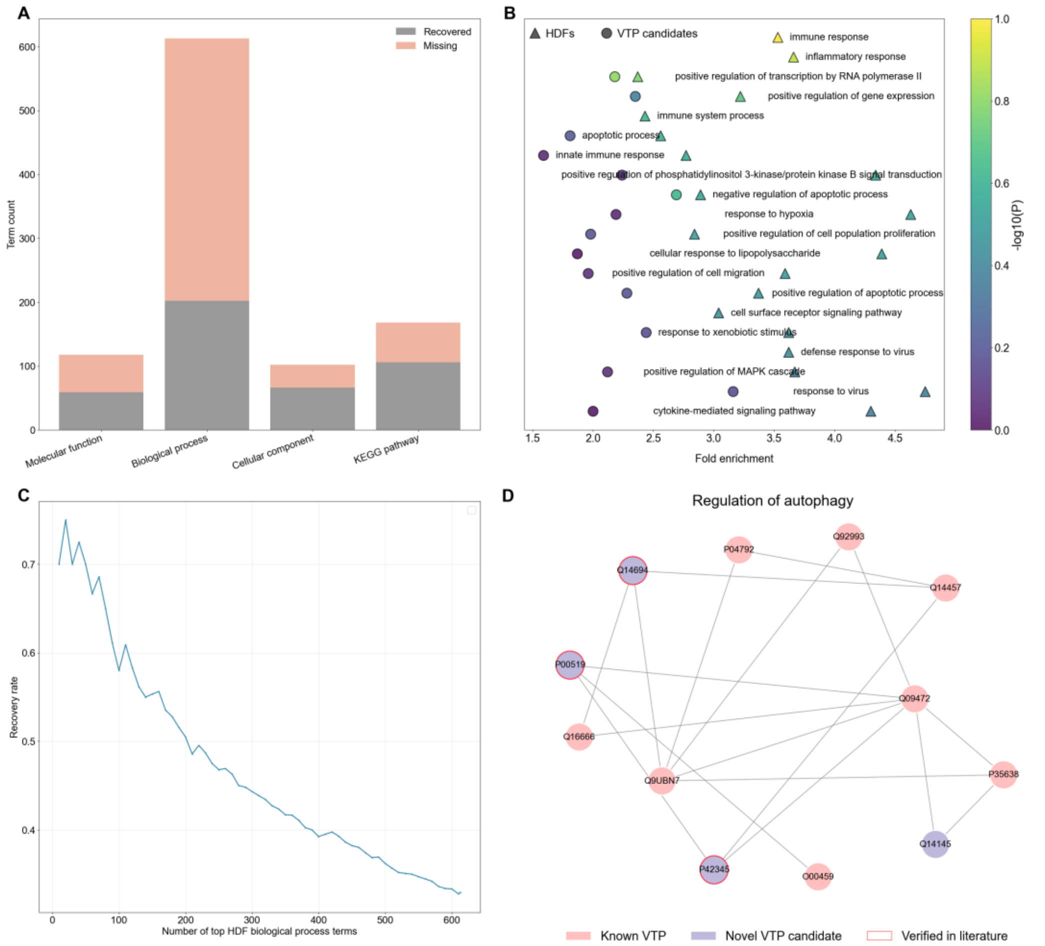


Figure K. Analysis of VTP candidates and known HDFs in HIV-1 infection. (A) Distribution of functional terms enriched in predicted VTPs and HDFs. The results are classified into two categories: recovered (overlapping terms between VTP candidates and HDFs), and missing (terms enriched exclusively in HDFs). (B) Top 20 biological processes enriched in HDFs. (C) Recovery rate of VTP candidates for the top k terms enriched in HDFs. (D) Interaction network of VTPs related to the 'regulation of autophagy' term.

### S1.12 Model re-evaluation using an updated dataset

We expanded the original dataset by incorporating the other two virus-host protein interaction databases, IntAct and BioGRID, and updated the labels of samples in this work [10, 11]. The number of newly added VTPs was most notable for HPV16, H5N1, HHV-8P, and SARS-CoV-2. Notably, the number of VTPs for SARS-CoV-2 increased by 2,363, reflecting the recent surge in research on COVID-19 virus-host interactions. For the other viruses included in this study, the number of newly identified VTPs was relatively limited, confirming the completeness of HVIDB for these viruses (Figure LA). In addition, we retrained the model using the updated dataset and evaluated its performance on the newly annotated test set. The model showed only limited AUPR improvement for most viruses, while relatively substantial gains were observed for those with a large number of newly added VTPs. Notably, the AUPR of our original model was merely 0.108 on the uncorrected SARS-CoV-2 test set. In contrast, its AUPR increased to 0.814 when evaluated on the updated test set (Figure LB). This result indicates that our model can effectively identify potential VTPs, even when trained on samples containing a high proportion of false negatives.

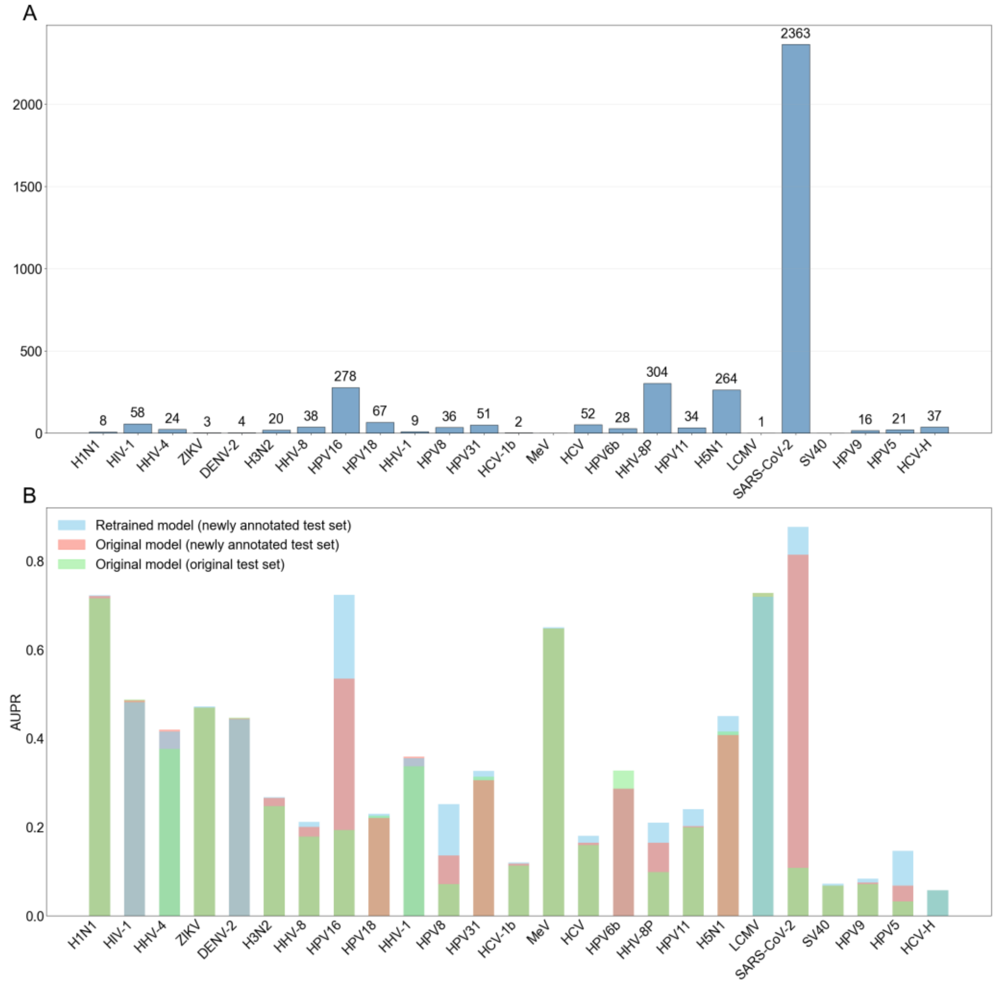


Figure L. Impact of database integration on VTP prediction. (A) Number of newly added VTPs for each virus. (B) Performance comparison of models trained on different data sources.

## S1.13 Comparison of different negative sampling strategies

To evaluate the effect induced by the quality of non-VTPs, we adopted two strategies to construct the training set. The first strategy utilizes the positive-unlabeled learning method with a spy technique. We randomly selected 15% of VTPs as 'spy samples' and combined them with non-VTPs to train an XGB classifier. We then set the lowest score covering 95% of the spy samples as the threshold, and considered non-VTPs with a score below this threshold as reliable negative samples. We retrained our model with this refined training set and evaluated its performance on test sets. The results showed that all metrics except recall decreased. This is likely because while the strategy successfully removes false negative samples, the selected non-VTPs differ markedly from VTPs in terms of their features. As a result, the model fails to adequately learn from 'difficult negative samples', and thus struggles to accurately predict non-VTPs that resemble genuine VTPs. For the second strategy, we performed functional enrichment analysis on VTPs and selected GO terms and KEGG pathways with a p-value < 0.05 and fold enrichment > 2 as more conserved functions for viral infection [12]. Non-VTPs not annotated to these terms were then regarded as reliable non-VTPs. The results indicated that the MCC, recall, precision, and F1 measures increased, while the AUPR measure remained stable (Table A). This could be attributed to the higher quality of negative samples and the reduction of label noise. Altogether, our analyses demonstrate that the initial non-VTP selection scheme reliably reflects the predictive power of our model.

Table A. Performance comparison of different negative sampling strategies

| Negative sampling strategy | $D_{virus}$ | | | | | |
|---|---|---|---|---|---|---|
| | MCC | AUPR | AUC | RC | F1 | Pre |
| MultiVTP | 0.347 | 0.375 | 0.839 | 0.433 | 0.370 | 0.390 |
| PU_spy | 0.323 | 0.362 | 0.830 | 0.558 | 0.336 | 0.314 |
| Not in BP | 0.372 | 0.376 | 0.840 | 0.482 | 0.390 | 0.408 |
| Not in CC | 0.373 | 0.375 | 0.840 | 0.507 | 0.390 | 0.395 |
| Not in KEGG | 0.376 | 0.375 | 0.841 | 0.482 | 0.393 | 0.421 |
| Not in any conserved terms | 0.372 | 0.371 | 0.838 | 0.469 | 0.390 | 0.417 |

### S1.14 MultiVTP extension for directional interaction prediction

The host proteins associated with viral infections could be divided into HDFs and HRFs according to their functions. Specifically, HDFs are host proteins essential for the virus to complete its life cycle, whereas HRFs function to inhibit viral infection and replication. To extend MultiVTP for interaction directionality prediction, we first annotated HIV-1 VTPs based on Chai et al.'s work and categorized them into forward (HDFs) and backward (HRFs) types [1]. We then expanded our model's output for HIV-1 into two dedicated channels, each corresponding to one directionality type. Results show that the two types of directional predictions achieved comparable performance, and the superior performance of HDF prediction could be attributed to the larger number of training samples for HDFs (695 samples) compared with HRFs (only 290 samples) (Figure M).

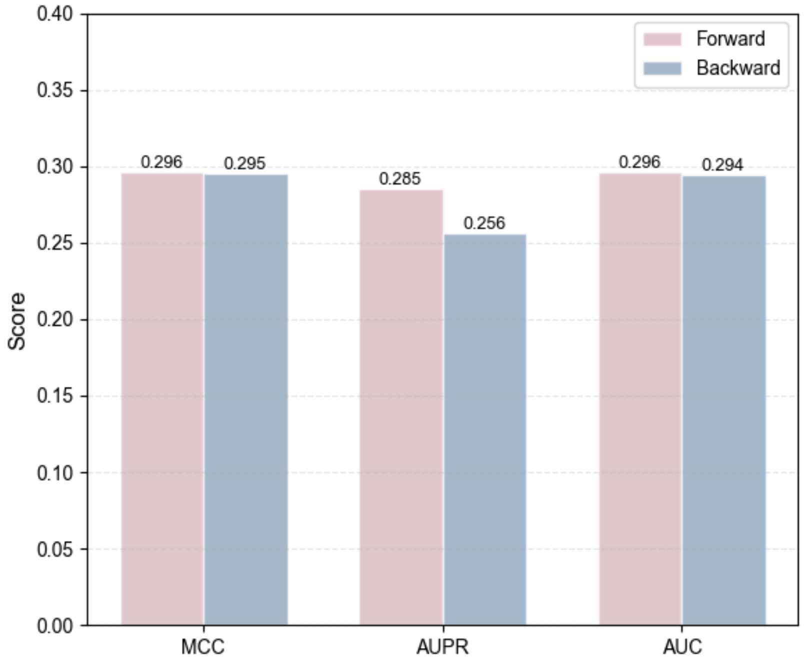


Figure M. Performance of MultiVTP for interaction directionality prediction.

### S1.15 Sensitivity analysis of VTP count thresholds

We tested four minimum VTP count thresholds (i.e., 100, 200, 300, and 400) based on 5-fold cross-validation. The results showed that the model maintained stable prediction performance for the corresponding viruses when the threshold was set to 200, 300, or 400. However, when the threshold was set to 100, the performance clearly declined for most viruses (Figure N). This may be attributed to the extreme imbalance between positive and negative samples, as well as the seesaw phenomenon and negative transfer in multi-task learning. Based on the results, we ultimately selected 200 as the minimum VTP count threshold.

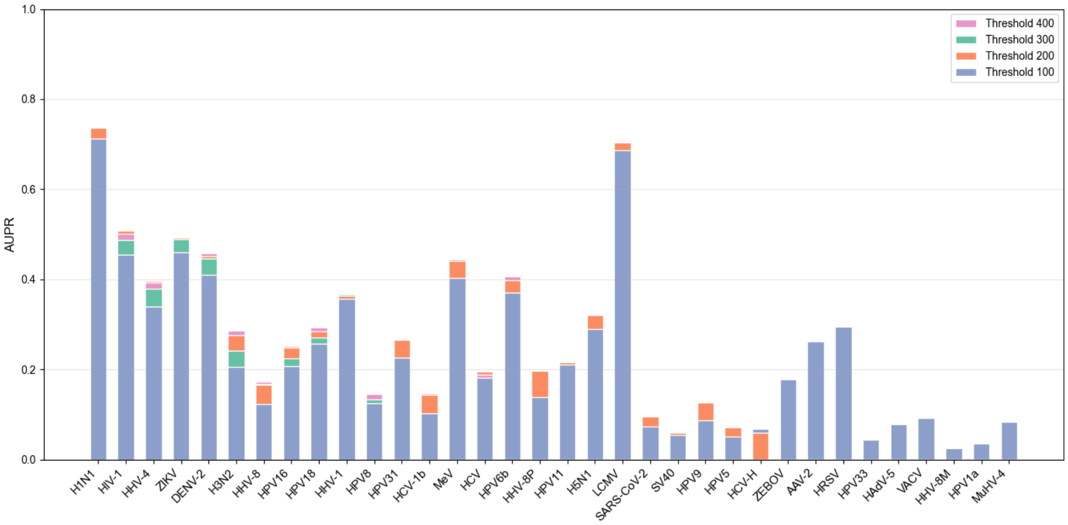


Figure N. Model performance based on different minimum VTP count thresholds.

**S1.16 Reasons for various decisions in building our model**

For global network embedding generation, we performed a grid search on the return parameter p and in-out parameter q in Node2vec. Through 5-fold cross-validation, we observed that the combination of p=4 and q=1 yielded the highest AUPR value (Figure O). For structural information, the four properties (α-helix, β-sheet, random coil, and solvent accessibility) effectively describe the basic structural characteristics of proteins and have been widely used for predicting protein functions and protein interactions. As for sequence representation, pretrained protein language models have become the primary feature extraction method in host-virus interaction prediction. In our main text, we compared several popular models such as ProtTrans, ESM2, and ProteinBERT, and found that ESM2 had the best performance. Regarding protein functional information, the GO database provides systematic annotations by categorizing protein functions from three perspectives (molecular function, biological process, and cellular component), enabling the effective annotation of host proteins. For functional similarity calculation, we employed GOsemSim, a widely used tool that supports multiple similarity qualification methods. Additionally, it can not only assess protein functional similarity but also evaluate semantic relationships between GO terms, thus meeting the analytical needs of our study.

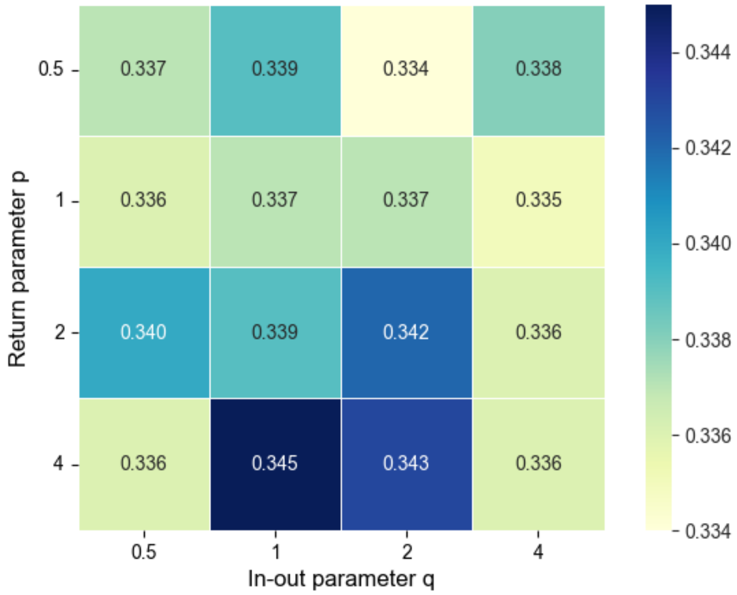


Figure O. Impact of return and in-out parameters in node2vec on prediction performance on the $D_{virus}$ dataset.

**Reference**


1. Chai H, Gu Q, Hughes J, Robertson DL. In silico prediction of HIV-1-host molecular interactions and their directionality. PLoS computational biology. 2022;18(2):e1009720.
2. Lo Surdo P, Iannuccelli M, Contino S, Castagnoli L, Licata L, Cesareni G, et al. SIGNOR 3.0, the SIGnaling network open resource 3.0: 2022 update. Nucleic acids research. 2023;51(D1):D631-d7.
3. McBride AA, Warburton A, Khurana S. Multiple Roles of Brd4 in the Infectious Cycle of Human Papillomaviruses. Frontiers in molecular biosciences. 2021;8:725794.
4. Pilcher C, Buco PAV, Truong JQ, Ramsland PA, Smeets MF, Walkley CR, et al. Characteristics of the Kelch domain containing (KLHDC) subfamily and relationships with diseases. FEBS letters. 2025;599(8):1094-112.
5. Zhang L, Wang S, Wang Y, Zhao T. HBFormer: a single-stream framework based on hybrid attention mechanism for identification of human-virus protein-protein interactions. Bioinformatics (Oxford, England). 2024;40(12).
6. Sledzieski S, Singh R, Cowen L, Berger B. D-SCRIPT translates genome to phenome with sequence-based, structure-aware, genome-scale predictions of protein-protein interactions. Cell systems. 2021;12(10):969-82.e6.
7. Luo Y, Zhang X, Chen R, Li R, Liu Y, Zhang J, et al. USP10 regulates B cell response to SARS-CoV-2 or HIV-1 nanoparticle vaccines through deubiquitinating AID. Signal transduction and targeted therapy. 2022;7(1):7.
8. Taylor HE, Calantone N, Lichon D, Hudson H, Clerc I, Campbell EM, et al. mTOR Overcomes Multiple Metabolic Restrictions to Enable HIV-1 Reverse Transcription and Intracellular Transport. Cell reports. 2020;31(12):107810.
9. Zhao J, Li Z, Wang M, Zhang Z, Ma H, Chang J, et al. Manipulation of autophagy by HCMV infection is involved in mTOR and influences the replication of virus. Acta biochimica et biophysica Sinica. 2013;45(11):979-81.
10. Del Toro N, Shrivastava A, Ragueneau E, Meldal B, Combe C, Barrera E, et al. The IntAct database: efficient access to fine-grained molecular interaction data. Nucleic acids research. 2022;50(D1):D648-d53.
11. Stark C, Breitkreutz BJ, Reguly T, Boucher L, Breitkreutz A, Tyers M. BioGRID: a general repository for interaction datasets. Nucleic acids research. 2006;34(Database issue):D535-9.
12. Ben-Hur A, Noble WS. Choosing negative examples for the prediction of protein-protein interactions. BMC bioinformatics. 2006;7 Suppl 1(Suppl 1):S2.
