# Supplementary material for: Multilabel prediction of virus target proteins via multimodal graph representation learning
Source: PLoS Comput Biol. 2026 May 26;22(5):e1014320. doi: 10.1371/journal.pcbi.1014320 (PMC13229406; doi:10.1371/journal.pcbi.1014320)
Supplement: S2 File — This document contains the following tables: Table A. Virus species and virus families in this work. Table B. Number of VTPs in the Dvirus dataset. Table C. Number of VTPs in the Dfamily dataset. Table D. Number of VTPs in the few-shot learning dataset across 5-fold cross-validation. Table E. Performance of protein embeddings with different models. Table F. Novel VTP candidates in the main text and their supporting literature. Table G. Literature-supported novel pathways enriched in H1N1 VTP candidates. (PDF) [file pcbi.1014320.s002.pdf]

Table A. Virus species and virus families in this work

Virus families in the D_{family} dataset	Virus species (abbreviation) in the D_{virus} dataset
Orthomyxoviridae	Influenza A virus-H1N1 (H1N1)
	Influenza A virus-H3N2 (H3N2)
	Influenza A virus-H5N1 (H5N1)
Flaviviridae	Zika virus (ZIKV)
	Dengue virus type 2 (DENV-2)
	Hepatitis C virus genotype 1b (HCV-1b)
	Hepacivirus C (HCV)
	Hepatitis C virus genotype 1a (HCV-H)
Herpesviridae	Human herpesvirus 4 (HHV-4)
	Human herpesvirus 8 (HHV-8)
	Human herpesvirus 1 (HHV-1)
	Human herpesvirus 8 type P (HHV-8P)
Papillomaviridae	Human papillomavirus type 16 (HPV16)
	Human papillomavirus type 18 (HPV18)
	Human papillomavirus type 8 (HPV8)
	Human papillomavirus type 31 (HPV31)
	Human papillomavirus type 6b (HPV6b)
	Human papillomavirus type 11 (HPV11)
	Human papillomavirus type 9 (HPV9)
	Human papillomavirus type 5 (HPV5)
Retroviridae	Human immunodeficiency virus type 1 (HIV-1)
Paramyxoviridae	Measles virus (MeV)
Arenaviridae	Lymphocytic choriomeningitis virus (LCMV)
Polyomaviridae	Simian virus 40 (SV40)
Coronaviridae	Severe acute respiratory syndrome coronavirus 2 (SARS-CoV-2)
Poxviridae	Not available
Parvoviridae	Not available

Table B. Number of VTPs in the D_{virus} dataset

Virus species	Train	Test
H1N1	1487	367
HIV-1	931	221
HHV-4	624	166
ZIKV	552	138
DENV-2	547	137
H3N2	370	87
HHV-8	352	94
HPV16	337	99
HPV18	348	83
HHV-1	341	70
HPV8	337	63
HPV31	294	85
HCV-1b	273	81
MeV	256	68
HCV	259	61
HPV6b	257	58
HHV-8P	203	59
HPV11	196	56
H5N1	193	49
LCMV	197	42
SARS-CoV-2	173	58
SV40	177	43
HPV9	179	39
HPV5	163	40
HCV-H	161	41

Table C. Number of VTPs in the D_{family} dataset

Virus family	Train	Test
Orthomyxoviridae	1635	391
Flaviviridae	1399	345
Herpesviridae	1332	352
Papillomaviridae	1262	312
Retroviridae	1040	245
Paramyxoviridae	353	86
Arenaviridae	203	44
Polyomaviridae	217	53
Coronaviridae	195	53
Poxviridae	182	35
Parvoviridae	160	42

Table D. Number of VTPs in the few-shot learning dataset across 5-fold cross-validation

Virus species	CV1	CV2	CV3	CV4	CV5
HDV	61/15	60/16	59/17	60/16	64/12
VACV	49/13	50/12	48/14	49/13	52/10
HTLV-1	42/9	42/9	40/11	40/11	40/11
MuHV-4	35/9	36/8	35/9	34/10	36/8
HEV-1	34/8	35/7	34/8	33/9	32/10
HAdV-5	19/5	19/5	20/4	18/6	20/4
RUBV	19/4	19/4	19/4	19/4	16/7
AAV-2	19/4	19/4	19/4	19/4	16/7
HPV33	17/4	17/4	17/4	17/4	16/5
HPV39	18/3	16/5	18/3	16/5	16/5

Table E. Performance of protein embeddings with different models

Embedding	Model	D_{virus}						D_{family}					
		MCC	AUPR	AUC	RC	F1	Pre	MCC	AUPR	AUC	RC	F1	Pre
ProteinBERT	LSTM	0.186	0.162	0.714	0.468	0.215	0.204	0.247	0.289	0.718	0.426	0.336	0.327
	CNN	0.210	0.188	0.732	0.383	0.230	0.294	0.230	0.280	0.716	0.489	0.326	0.286
	MLP	0.219	0.202	0.739	0.426	0.238	0.299	0.253	0.304	0.735	0.479	0.341	0.324
ProtTrans	LSTM	0.220	0.196	0.741	0.436	0.247	0.247	0.274	0.321	0.752	**0.506**	0.357	0.325
	CNN	0.231	0.214	0.742	0.411	0.256	0.281	0.291	0.348	0.760	0.494	0.373	0.358
	MLP	0.256	0.230	0.772	**0.469**	0.272	0.291	0.286	0.344	0.760	0.464	0.370	**0.366**
ESM2	LSTM	0.264	0.242	0.780	0.427	0.282	0.314	0.293	0.341	0.765	0.490	0.378	0.349
	CNN	0.262	0.240	0.774	0.420	0.281	0.324	0.290	0.342	0.765	0.500	0.374	0.348
	MLP	**0.269**	**0.250**	**0.781**	0.429	**0.282**	**0.338**	**0.305**	**0.353**	**0.772**	0.496	**0.386**	0.359
GO_PubMedBERT	MLP	0.255	0.231	0.771	**0.455**	0.273	0.303	0.294	0.350	**0.769**	**0.531**	0.378	0.342
	GCN	**0.272**	**0.254**	**0.779**	0.432	**0.288**	**0.336**	**0.305**	**0.364**	0.767	0.505	**0.384**	**0.373**

Table F. Novel VTP candidates in the main text and their supporting literature

UniProt ID	Gene	Virus	PMID
P24390	KDELR1	DENV-2	25753416
Q96MU7	YTHDC1	H1N1	41066792
P05362	ICAM1	H1N1	26499045
P08069	IGF1R	H1N1	31849847
P61224	RAP1B	H1N1	39474066
P67870	CSNK2B	H1N1	37938186
P27361	MAPK3	H1N1	22822053
P68400	CSNK2A1	H1N1	37938186
P28482	MAPK1	H1N1	32709018
P61586	RHOA	H1N1	23283961
P12830	CDH1	H1N1	36351985
Q01094	E2F1	HIV-1	17011204
P42345	MTOR	HIV-1	32579936
P01116	KRAS	HIV-1	35232997
Q07812	BAX	HIV-1	21653671
P01106	MYC	HIV-1	34277639
P49841	GSK3B	HIV-1	25616162
Q15796	SMAD2	HIV-1	40632811
P06400	RB1	HIV-1	22701041
P31749	AKT1	HIV-1	41032412
P38936	CDKN1A	HIV-1	32615986
Q9H9S3	SEC61A2	H1N1, HIV-1, DENV-2	34769437
O43402	EMC8	DENV-2, ZIKV	31067454
Q58FF8	HSP90AB2P	SARS-CoV-2, HSV-1, EV71	37011862, 39948206

Table G. Literature-supported novel pathways enriched in H1N1 VTP candidates

KEGG pathway	Biological process	PMID
Pathogenic Escherichia coli infection	Escherichia coli Nissle 1917 activates host defense immunity in the lung, producing an immediate protective effect against H1N1 infection.	39122688
Adherens Junction	H1N1 infection disrupts the epithelial barrier by damaging AJ.	32610119
RNA Degradation	H1N1 infection caused rapid host RNA degradation.	29203926
Regulation of Actin Cytoskeleton	H1N1 infection regulates the dynamics of the actin cytoskeleton.	30634554
Apoptosis	H1N1can prevent cellular apoptosis and permit sufficient time for the replication and production.	31667646
Fatty Acid Metabolism	Fatty acid widely involved in the regulation of H1N1 infection.	33807642
