# Supplementary material for: Multilabel prediction of virus target proteins via multimodal graph representation learning
Source: PLoS Comput Biol. 2026 May 26;22(5):e1014320. doi: 10.1371/journal.pcbi.1014320 (PMC13229406; doi:10.1371/journal.pcbi.1014320)
Supplement: S3 File — This document contains the following figures: Fig A. Statistical analysis of traditional features of samples in the Dvirus dataset. Fig B. Statistical differences in traditional features between different sample groups in the Dvirus dataset. Fig C. Feature analysis and comparison on the Dfamily dataset. Fig D. Statistical analysis of traditional features of samples in the Dfamily dataset. Fig E. Statistical differences in traditional features between different sample groups in the Dfamily dataset. Fig F. SHAP analysis of traditional features. Fig G. t-SNE visualization of global topological properties for non-VTPs (gray points) and VTPs from the Dvirus dataset. Fig H. Distribution of GO similarity among VTPs and between VTPs and non-VTPs for each virus. Fig I. t-SNE visualization of global topological properties for non-VTPs (gray points) and VTPs from the Dfamily dataset. Fig J. Ablation experiments at the feature level. Fig K. Interpretability and ablation studies on the Dfamily dataset. Fig L. Multilabel prediction strategies in machine learning. Figure M. Performance comparison of MultiVTP and baseline methods on the Dfamily dataset. Fig N. Distribution of KEGG pathways enriched in predicted and known VTPs. Fig O. Analysis of HIV-1 VTP candidates. Fig P. Evaluation of our model using overlapping and virus-specific VTPs. Fig Q. Analysis of predicted and known VTPs in the human proteome across virus families. (PDF) [file pcbi.1014320.s003.pdf]

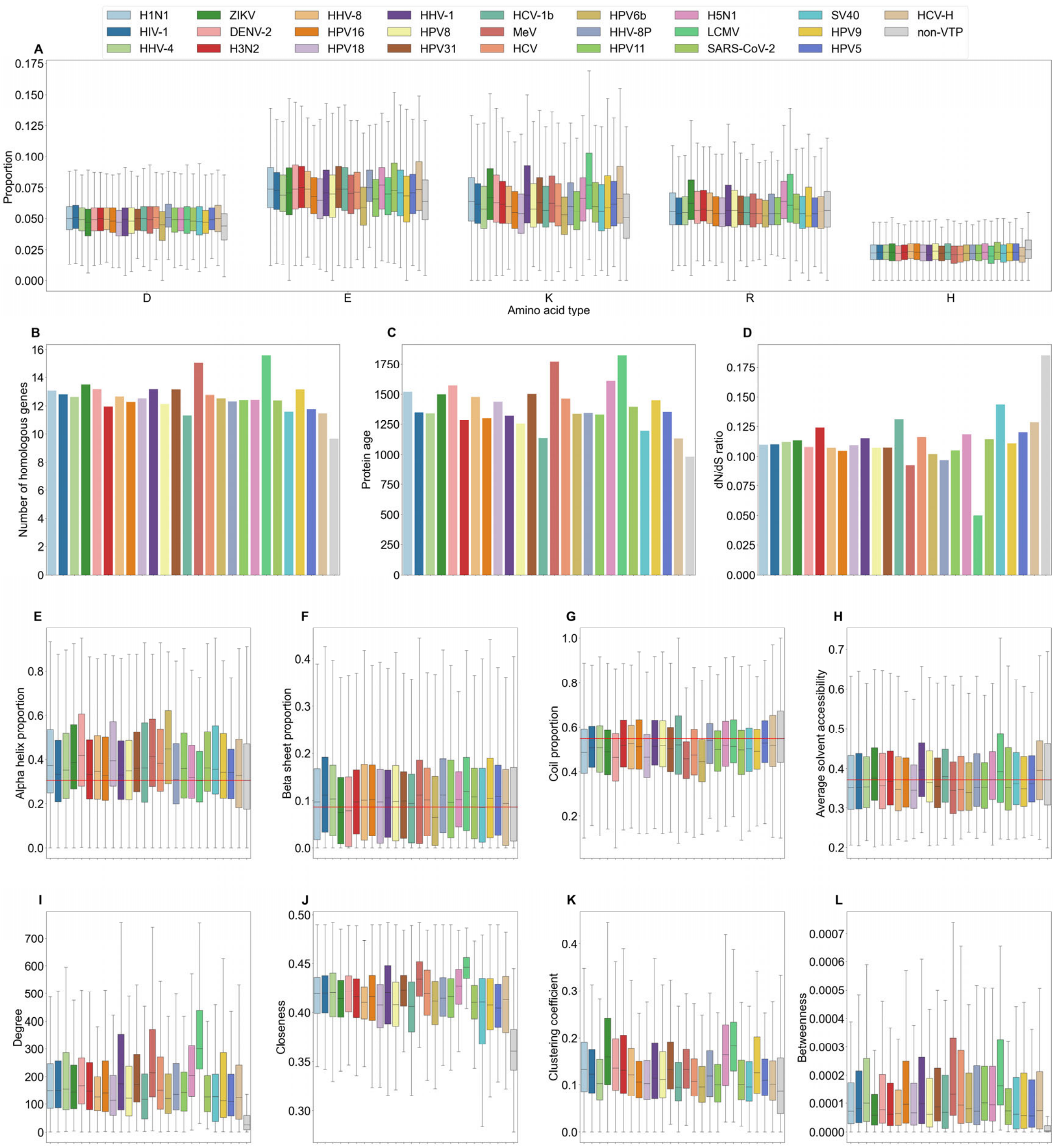

Fig A. Statistical analysis of traditional features of samples in the D_{virus} dataset. (A) Amino acid composition. (B) Number of homologous genes. (C) Protein age. (D) dN/dS ratio. (E) Alpha helix proportion. (F) Beta sheet proportion. (G) Coil proportion. (H) Average solvent accessibility. (I) Degree. (J) Closeness. (K) Clustering coefficient. (L) Betweenness.

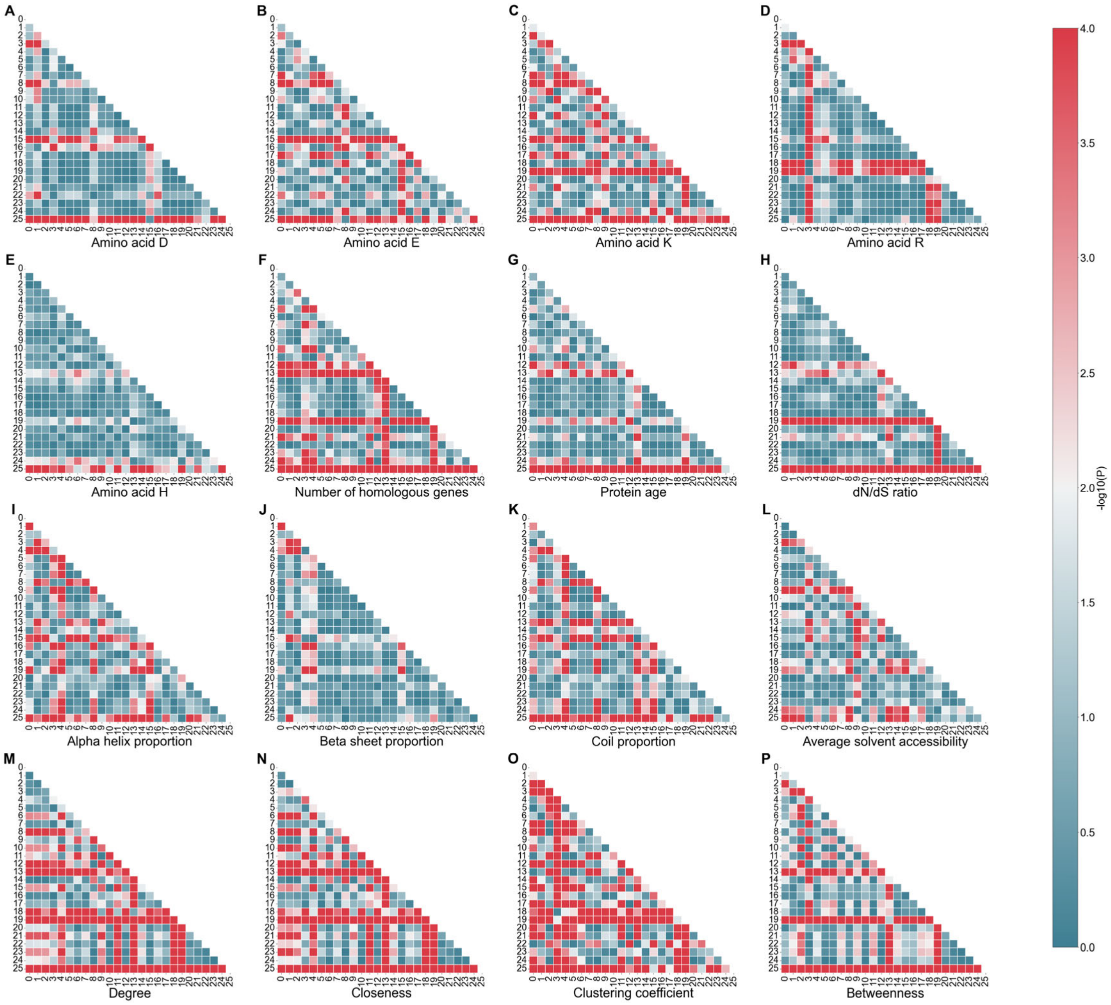

Fig B. Statistical differences in traditional features between different sample groups in the D_{virus} dataset (Wilcoxon rank sum tests). (A) Aspartic acid composition. (B) Glutamic acid composition. (C) Lysine composition. (D) Arginine composition. (E) Histidine composition. (F) Number of homologous genes. (G) Protein age. (H) dN/dS ratio. (I) Alpha helix proportion. (J) Beta sheet proportion. (K) Coil proportion. (L) Average solvent accessibility. (M) Degree. (N) Closeness. (O) Clustering coefficient. (P) Betweenness. 0-25: H1N1, HIV-1, HHV-4, ZIKV, DENV-2, H3N2, HHV-8, HPV16, HPV18, HHV-1, HPV8, HPV31, HCV-1b, MeV, HCV, HPV6b, HHV-8P, HPV11, H5N1, LCMV, SARS-CoV-2, SV40, HPV9, HPV5, HCV-H, and non-VTP.

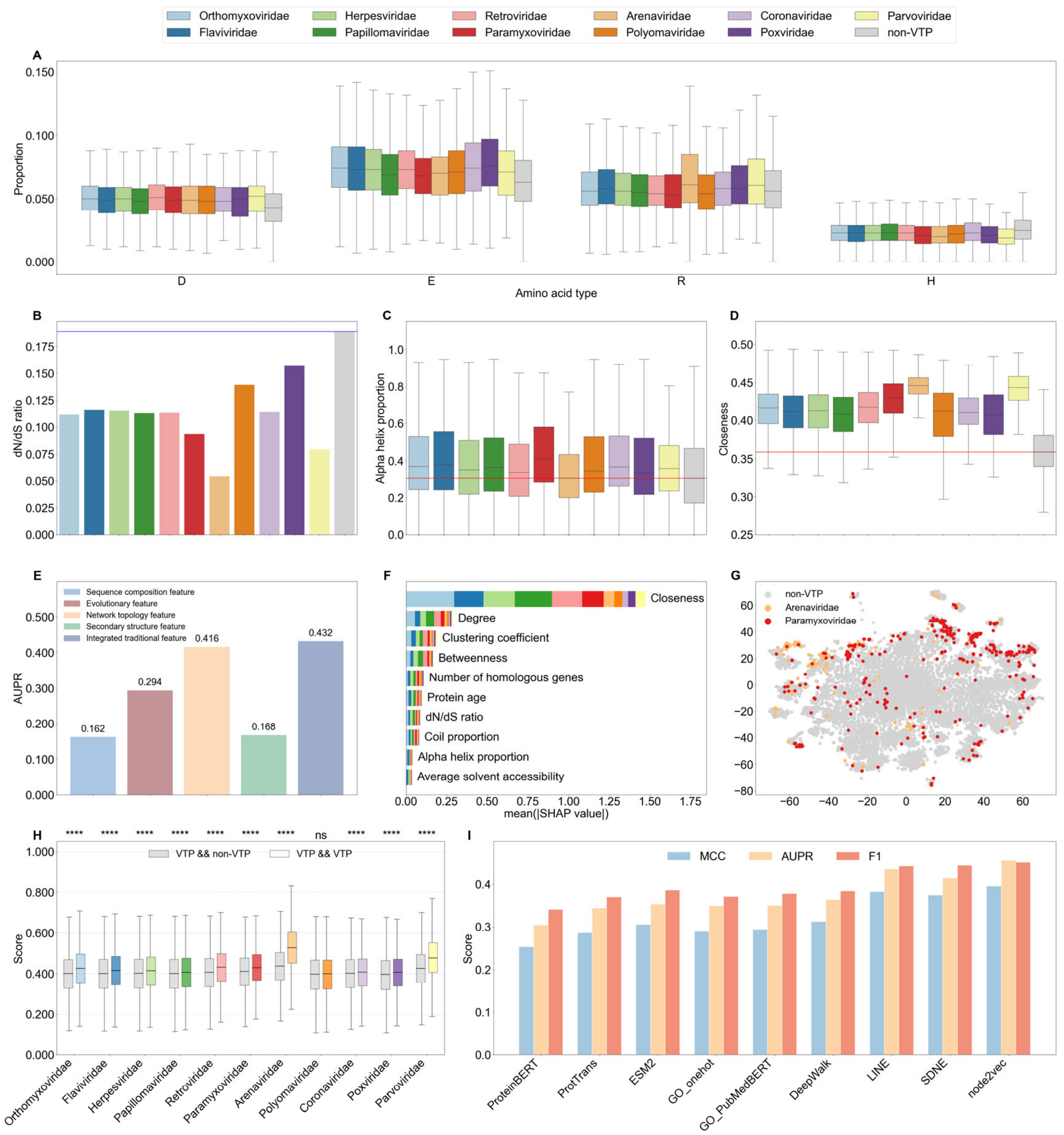

Fig C. Feature analysis and comparison on the D_{family} dataset. Statistical analysis of traditional features: (A) amino acid composition, (B) dN/dS ratio, (C) predicted coil proportion, and (D) closeness. (E) AUPRs achieved by different types of traditional features. (F) SHAP analysis of traditional features. (G) t-SNE visualization of global topological properties for VTPs and non-VTPs. (H) Gene ontology similarity among VTPs and between VTPs and non-VTPs for each virus families. Significant differences are evaluated using Wilcoxon rank sum tests. **** $p < 0.0001$, *** $0.0001 \leq p < 0.001$, ** $0.001 \leq p < 0.01$, * $0.01 \leq p < 0.05$, and ns: $p \geq 0.05$. (I) Performance of different protein embeddings.

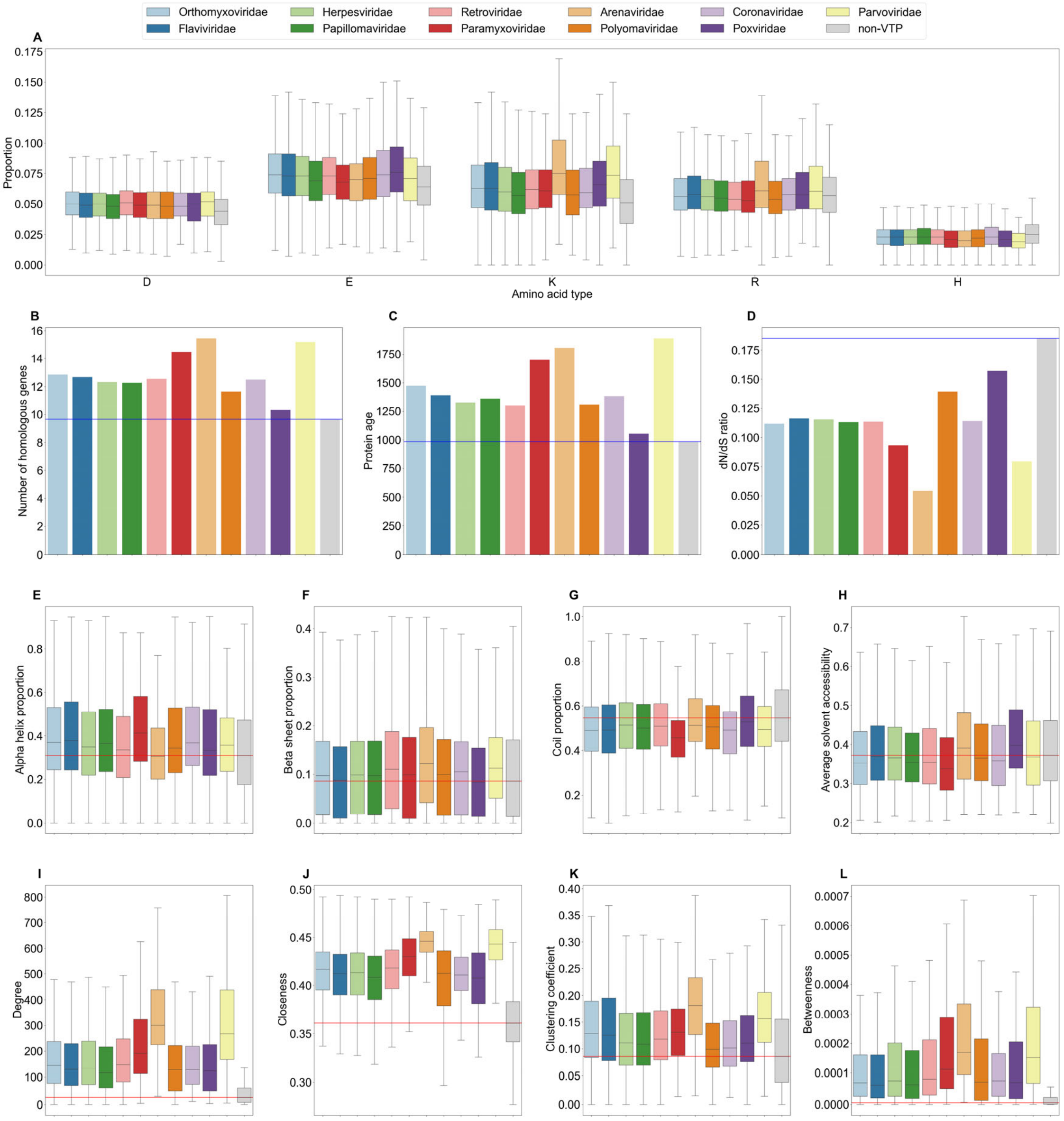

Fig D. Statistical analysis of traditional features of samples in the D_{family} dataset. (A) Amino acid composition. (B) Number of homologous genes. (C) Protein age. (D) dN/dS ratio. (E) Alpha helix proportion. (F) Beta sheet proportion. (G) Coil proportion. (H) Average solvent accessibility. (I) Degree. (J) Closeness. (K) Clustering coefficient. (L) Betweenness.

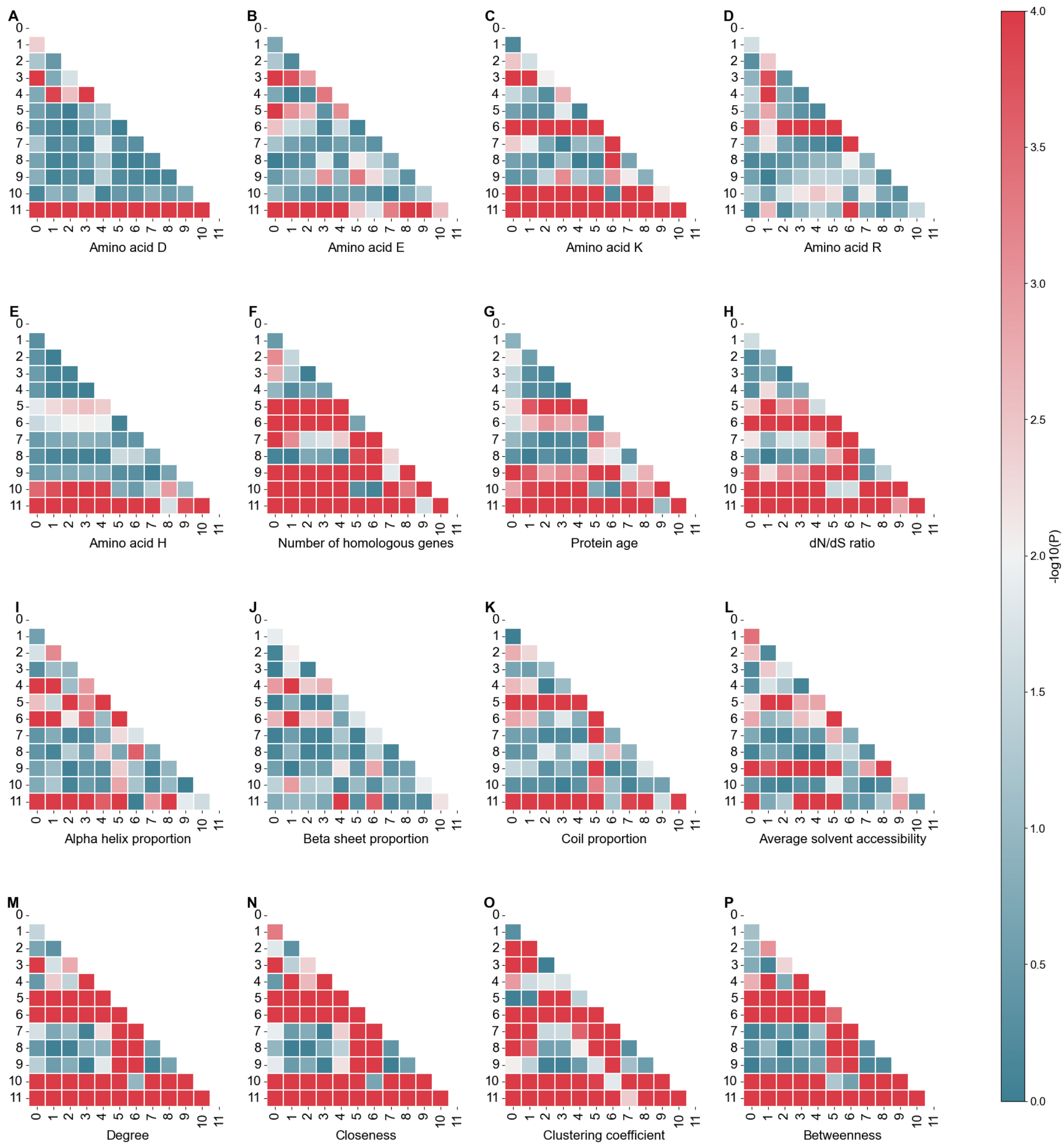

Fig E. Statistical differences in traditional features between different sample groups in the D_{family} dataset (Wilcoxon rank sum tests). (A) Aspartic acid composition. (B) Glutamic acid composition. (C) Lysine composition. (D) Arginine composition. (E) Histidine composition. (F) Number of homologous genes. (G) Protein age. (H) dN/dS ratio. (I) Alpha helix proportion. (J) Beta sheet proportion. (K) Coil proportion. (L) Average solvent accessibility. (M) Degree. (N) Closeness. (O) Clustering coefficient. (P) Betweenness. 0-11: Orthomyxoviridae, Flaviviridae, Herpesviridae, Papillomaviridae, Retroviridae, Paramyxoviridae, Arenaviridae, Polyomaviridae, Coronaviridae, Poxviridae, Parvoviridae, and non-VTP.

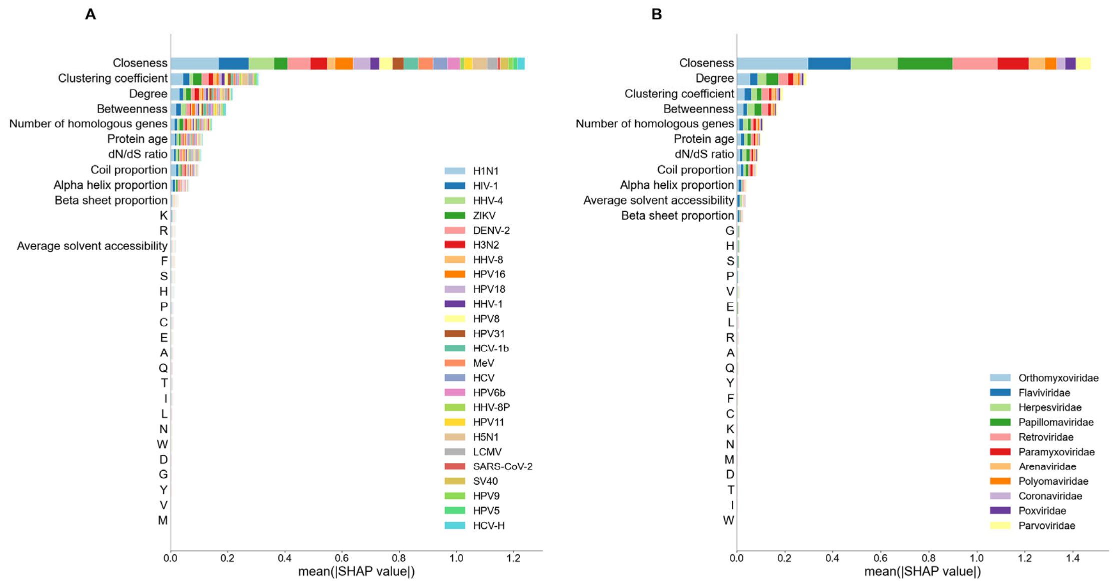

Fig F. SHAP analysis of traditional features. (A) Results on the D_{virus} dataset, and (B) Results on the D_{family} dataset.

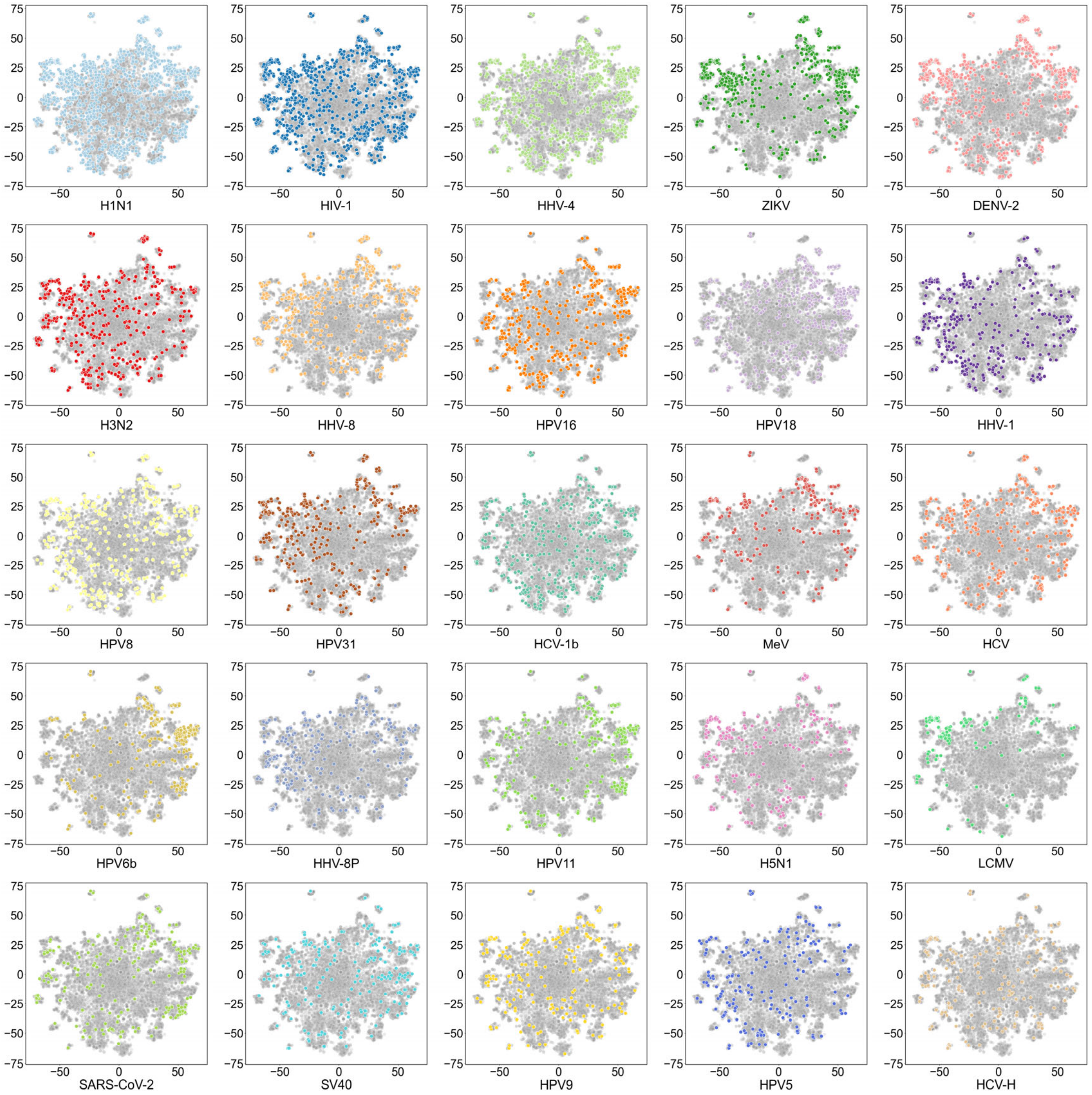

Fig G. t-SNE visualization of global topological properties for non-VTPs (gray points) and VTPs from the D_{virus} dataset.

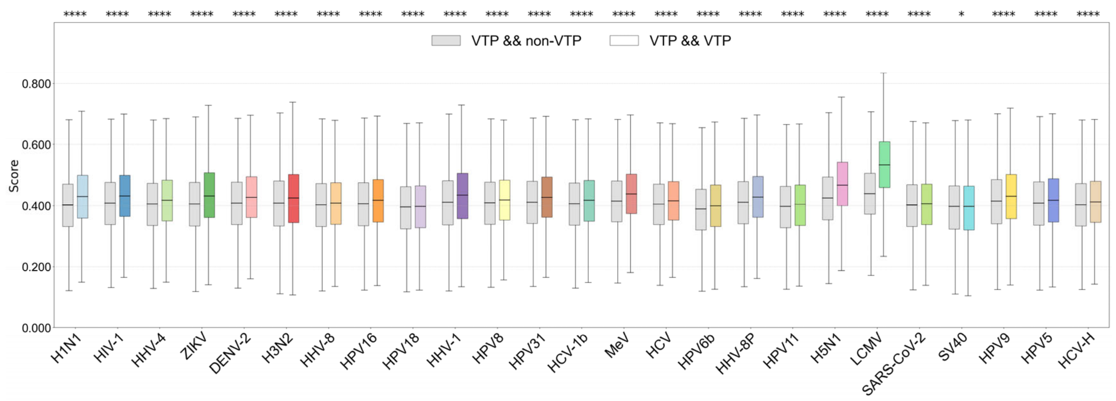

Fig H. Distribution of GO similarity among VTPs and between VTPs and non-VTPs for each virus. Significant differences are evaluated using Wilcoxon rank sum tests. **** $p < 0.0001$, *** $0.0001 \leq p < 0.001$, ** $0.001 \leq p < 0.01$, * $0.01 \leq p < 0.05$, and ns: $p \geq 0.05$.

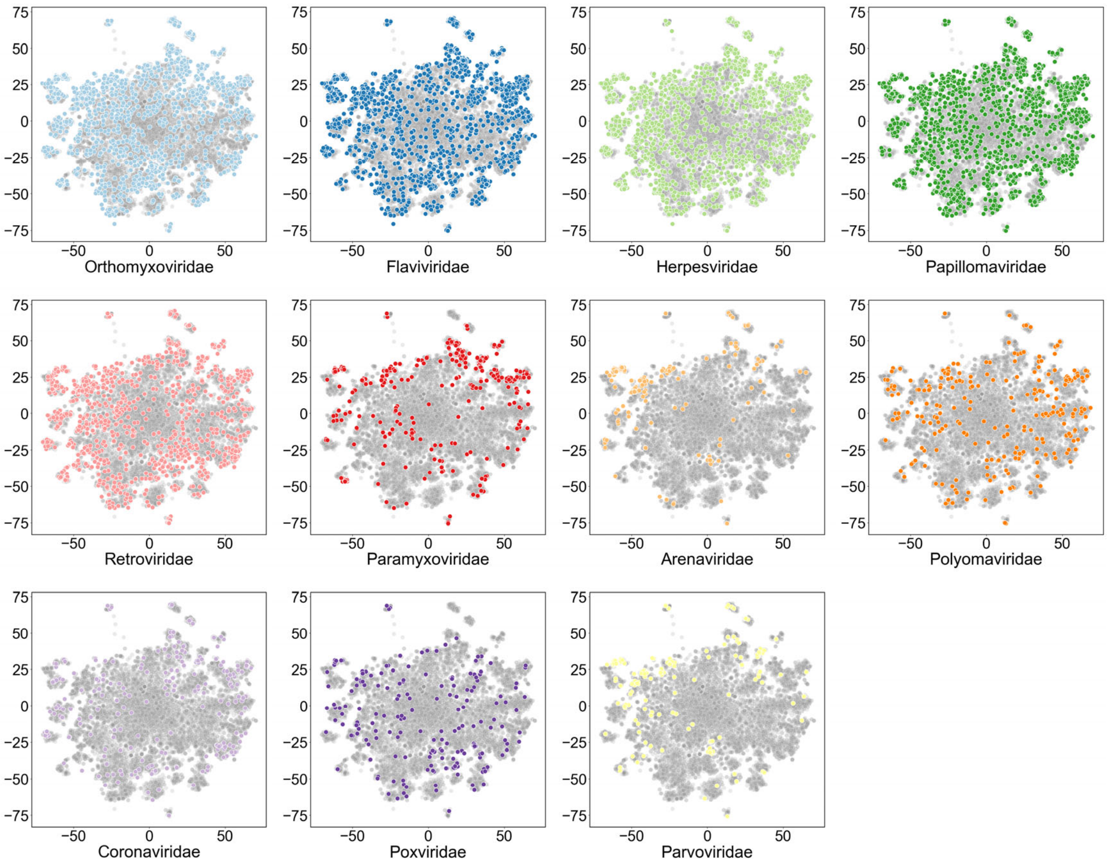

Fig I. t-SNE visualization of global topological properties for non-VTPs (gray points) and VTPs from the D_{family} dataset.

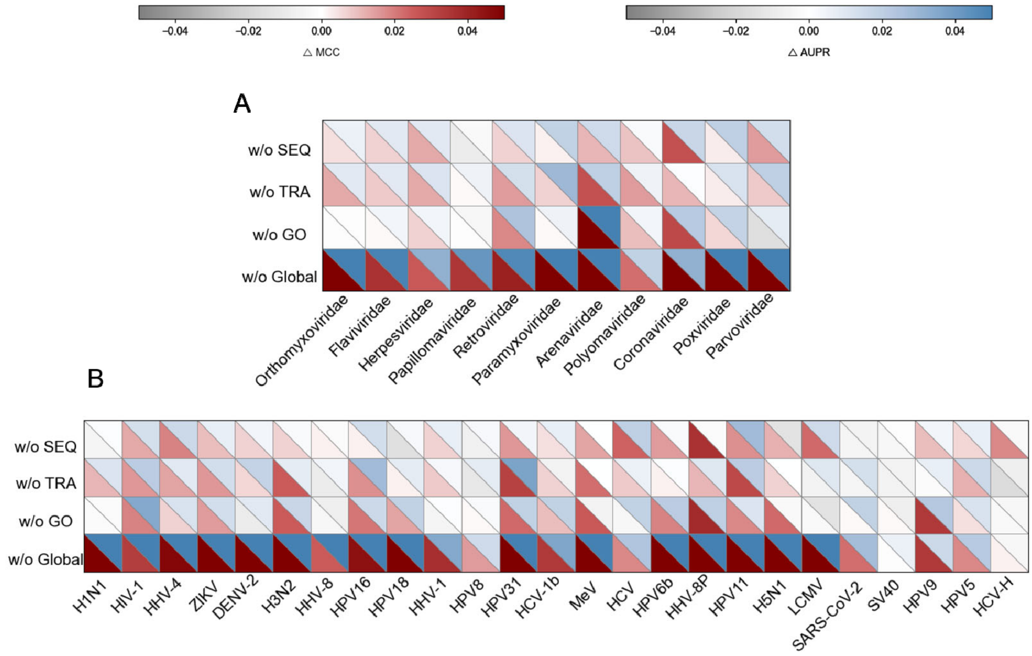

Fig J. Ablation experiments at the feature level. (A) Results on the D_{family} dataset, and (B) Results on the D_{virus} dataset.

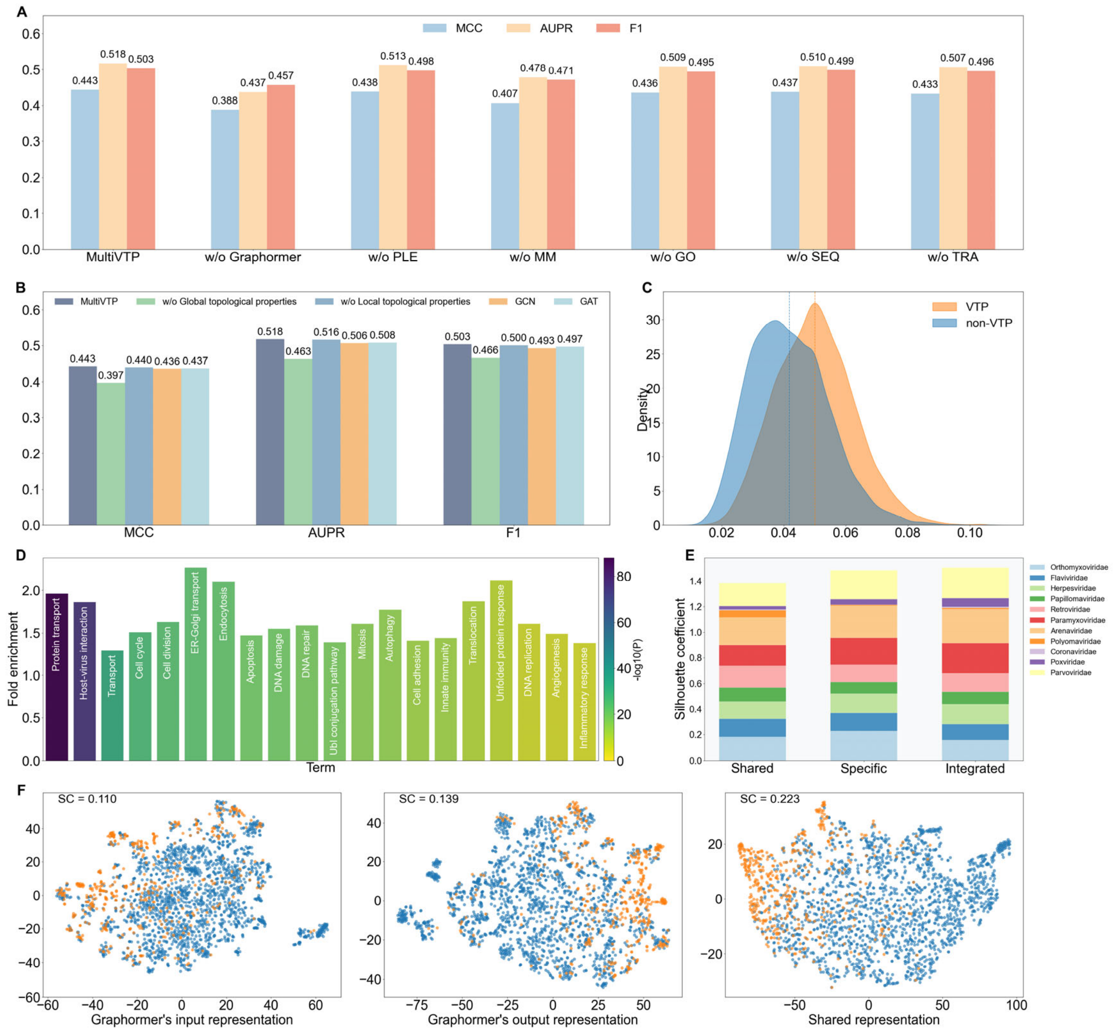

Fig K. Interpretability and ablation studies on the D_{family} dataset. (A) Ablation studies at both feature and module levels. MM: multimodal features, GO: functional features, SEQ: sequence features, and TRA: traditional features. (B) Ablation experiments for the components of Graphormer. (C) Density distribution of attention values of VTPs and non-VTPs. (D) Top 20 biological processes enriched in the top 50% of proteins sorted by attention scores. (E) Silhouette coefficients for evaluating the separation of different samples through t-SNE reduction. (F) t-SNE visualization of representations of VTPs (orange points) and non-VTPs (blue points).

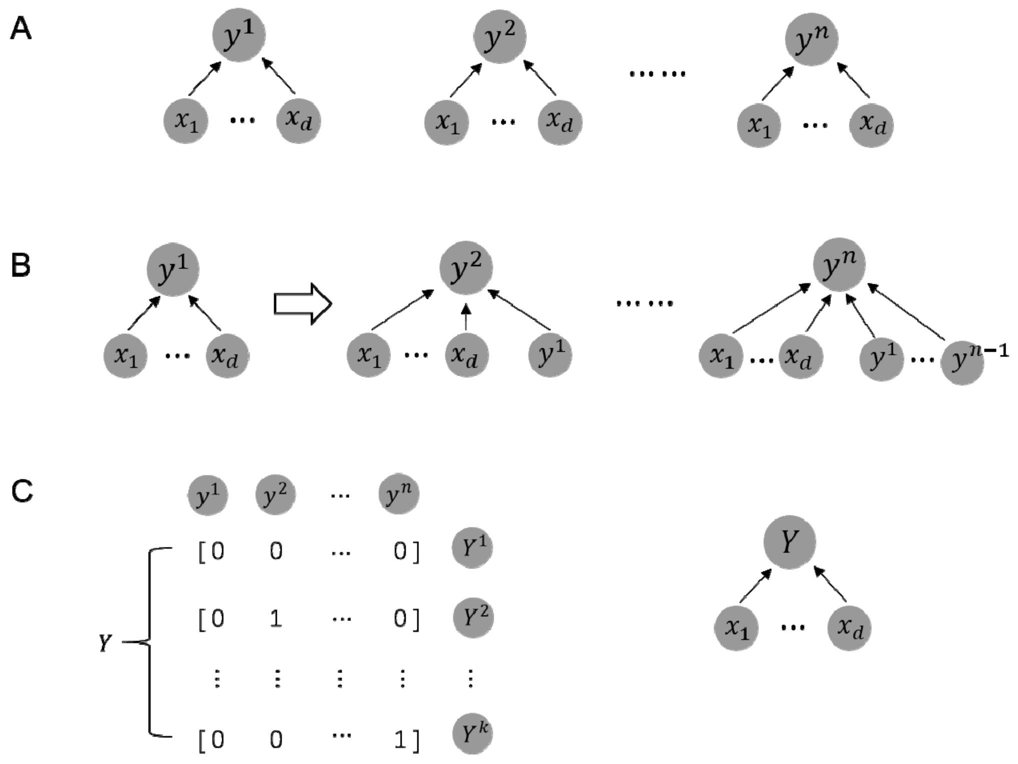

Fig L. Multilabel prediction strategies in machine learning. (A) Binary relevance (BR). BR treats each label as an independent binary classification task. (B) Classifier chains (CC). The problem is converted into a chain of binary classifiers, where the output of each preceding classifier is used as an additional input feature for the next classifier. (C) Label powerset (LP). Each unique combination of labels present in the training data is treated as a distinct class, and the multilabel problem is converted into a multiclass prediction task.

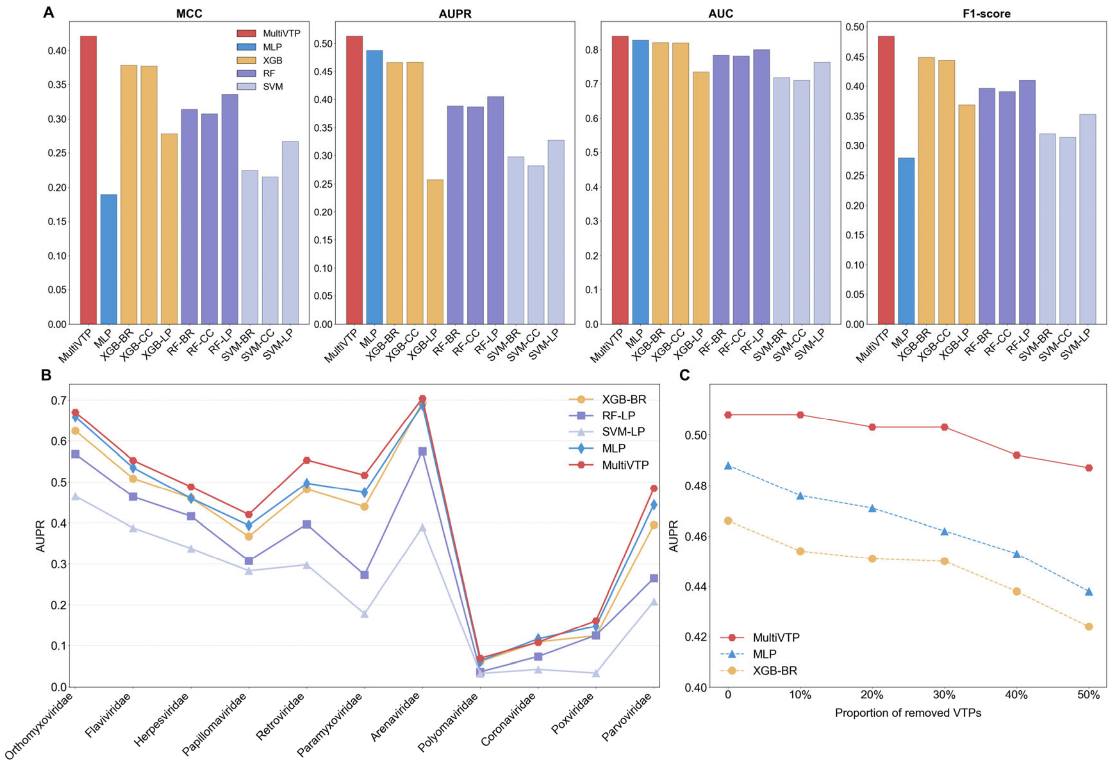

Fig M. Performance comparison of MultiVTP and baseline methods on the D_{family} dataset. (A) Performance of MultiVTP and machine learning methods. BR: binary relevance, CC: classifier chains, and LP: label powerset. (B) Comparison of AUPR between MultiVTP and baseline methods (i.e., MLP and other machine learning models with the optimal learning strategy). Virus families are sorted from left to right in descending order of VTP counts. (C) Performance of various approaches after removing different fractions of training VTPs.

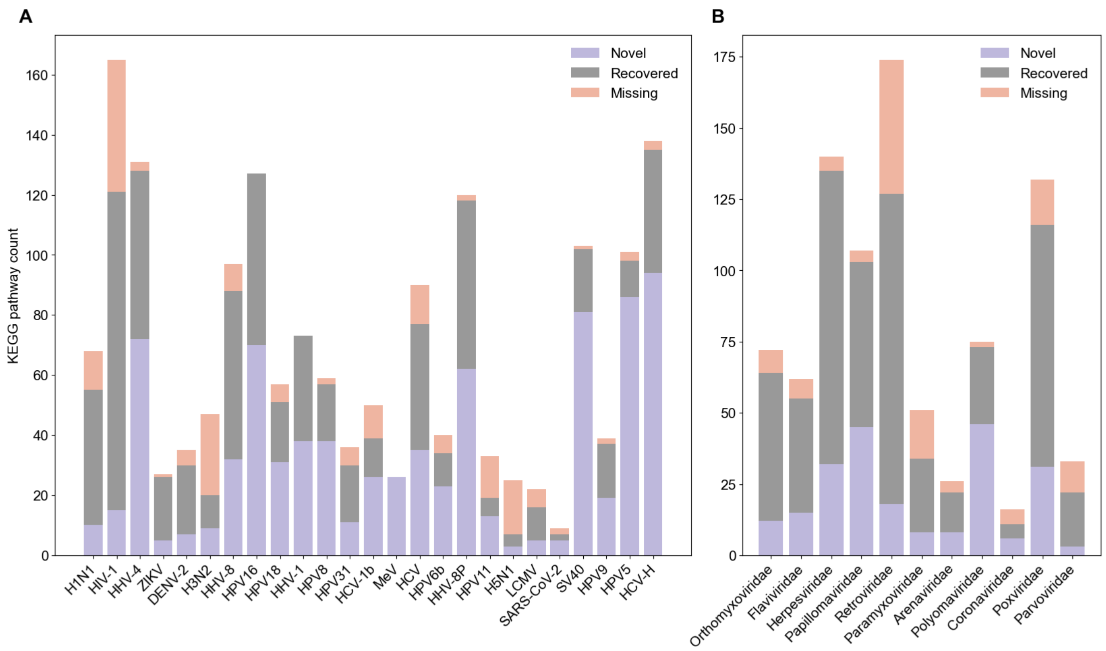

Fig N. Distribution of KEGG pathways enriched in predicted and known VTPs. (A) Results for virus species. (B) Results for virus families. The results are classified into three categories: novel (terms enriched exclusively in predicted VTPs), recovered (overlapping terms between predicted and known VTPs), and missing (terms enriched exclusively in known VTPs).

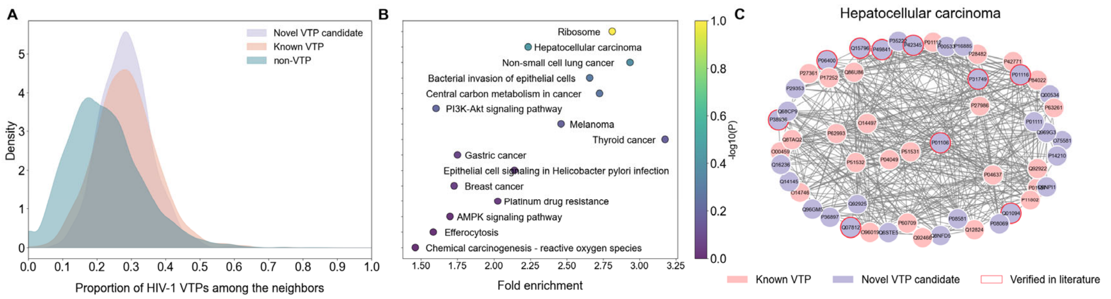

Fig O. Analysis of HIV-1 VTP candidates. (A) Density map of proportion of HIV-1 VTPs among the interacting neighbors of different samples. (B) Novel pathways related to HIV-1 VTP candidates. (C) Interaction network of relevant VTPs in the hepatocellular carcinoma pathway.

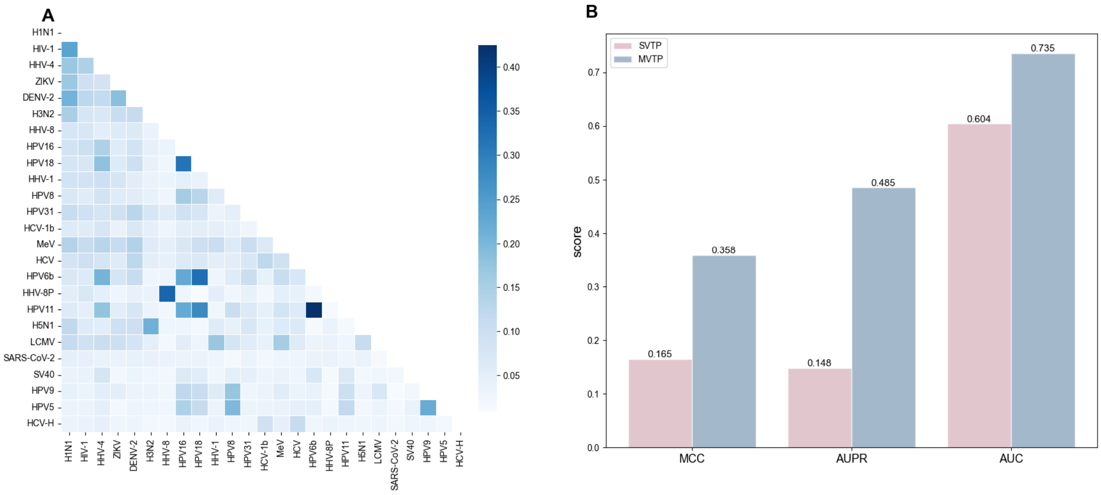

Fig P. Evaluation of our model using overlapping and virus-specific VTPs. (A) Overlap of VTPs among different viruses by Jaccard indices. (B) Performance of our model on SVTPs and MVTPs.

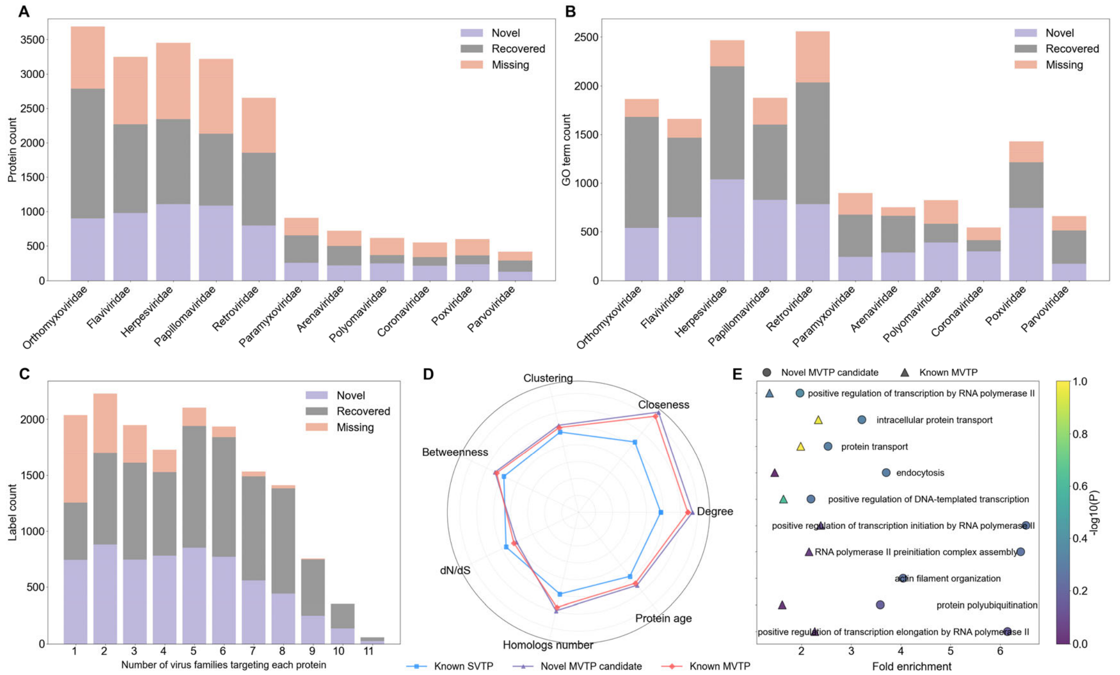

Fig Q. Analysis of predicted and known VTPs in the human proteome across virus families. (A) Distribution of predicted and known VTPs for each virus families. The results are classified into three categories: novel (newly predicted VTPs), recovered (overlap between predicted and known VTPs), and missing (known but unrecognized VTPs). (B) Distribution of GO terms enriched in predicted and known VTPs for each virus families. The results are classified into three categories: novel (terms enriched exclusively in predicted VTPs), recovered (overlapping terms between predicted and known VTPs), and missing (terms enriched exclusively in known VTPs). (C) Distribution of novel, recovered, and missing labels. The classification is similar to that of VTPs. (D) Network topology and evolutionary conservation attributes of SVTPs and MVTPs. SVTP: single virus family target protein, and MVTP: multiple virus families target protein. (E) Top 10 biological processes enriched in MVTPs.
