# Supplementary material for: Multilabel prediction of virus target proteins via multimodal graph representation learning
Source: PLoS Comput Biol. 2026 May 26;22(5):e1014320. doi: 10.1371/journal.pcbi.1014320 (PMC13229406; doi:10.1371/journal.pcbi.1014320)
Supplement: Attachment — Submitted filename: Response to Reviewers.pdf [file pcbi.1014320.s004.pdf]

Dear Editor and Reviewers,

We greatly appreciate your efforts to improve our manuscript. In response to your insightful comments and suggestions, we have carefully revised the original manuscript, and all revisions are highlighted in red in the current version. Owing to space limitations in the main text, the additional experiments recommended by the reviewers and their corresponding results are presented in Text S1. This document contains the following sections: S1.1 Analysis of model specificity in predicting host-virus relationships. S1.2 Analysis of model specificity in predicting VTPs within shared pathways. S1.3 Impact of sample similarity on prediction performance. S1.4 Network dependency analysis of MultiVTP. S1.5 Effect of PPI data quality on model performance. S1.6 Evaluation of simplified MultiVTP architectures. S1.7 Interpretability analysis via linking attention weights. S1.8 Comparison of MultiVTP with virus-host PPI prediction methods. S1.9 Robustness assessment under extreme data scarcity and unseen viruses. S1.10 Generalization to novel viruses with limited samples. S1.11 Comparative analysis of HIV-1 VTP candidates and HDFs. S1.12 Model re-evaluation using an updated dataset. S1.13 Comparison of different negative sampling strategies. S1.14 MultiVTP extension for directional interaction prediction. S1.15 Sensitivity analysis of VTP count thresholds. S1.16 Reasons for various decisions in building our model.

Yours sincerely,

Rong Liu
College of Informatics
Huazhong Agricultural University
Wuhan 430070, China

Response to reviewer 1

Comment 1: The focus of this research is on the interaction of viruses with human proteins, thereby infecting humans with certain diseases. There is no discussion about the factors that cause this interaction. For example, does the presence of identical motifs between the virus and the protein cause this interaction?

Response: Thank you for your comment. To determine whether shared motifs underlie virus-host protein interactions, we collected viral and host protein sequences from the HVIDB database and performed motif annotation using the InterPro database. The results showed that only 1% of host-virus protein interaction pairs shared at least one common motif, while a similar proportion (0.7%) of non-interacting protein pairs also exhibited shared motifs (Figure R1). This suggested that identical motifs may not be a primary driver of these interactions. Instead, motif mimicry has been proposed as a plausible intrinsic factor governing host-virus protein interactions. Specifically, viral proteins can mimic host short linear motifs to evade immunity and hijack host proteins (PMID: 39477943). In addition to motif mimicry, the physicochemical and structural

properties mentioned in our manuscript are also critical. For instance, viral proteins frequently carry a positive charge and thus tend to interact with negatively charged host proteins via electrostatic attraction (PMID: 36851498). Additionally, viral proteins can bind to host proteins by leveraging α-helices, which serve as common recognition elements in protein-protein interactions and provide stable interfaces for binding (PMID: 17350572, 19454348). The revision is provided in Lines 154-155 and 163-165.

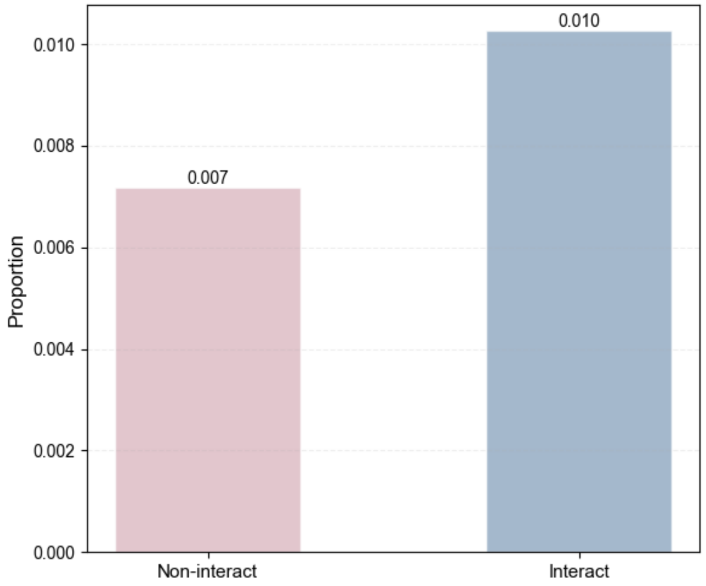

Figure R1. Proportion of protein pairs sharing at least one common motif.

Comment 2: This research claims existing approaches remain time-consuming. It would be good to talk about the specific existing approach they talk about and show that the developed method outperforms those previously developed ones.

Response: Thank you for your comment. The statement 'remain time-consuming and costly' specifically refers to experimental approaches for identifying VTPs. Currently, we compared our algorithm with existing PPI prediction methods (e.g., D-SCRIPT and HBFormer) for VTP prediction. D-SCRIPT is a sequence-based prediction model with strong generalization in cross-species interaction prediction, whereas HBFormer uses a hybrid attention transformer combined with multimodal features for host-virus PPI prediction. Because these models were originally designed for binary PPI predictions, we adjusted the prediction strategy to ensure a fair comparison. Specifically, each host protein was paired with all viral proteins in the HVIDB database, and the highest score among all PPI predictions was used as the prediction score for the host protein. The results suggested that MultiVTP remarkably outperformed the baseline models in terms of AUPR across all viruses (Figure R2). This may be because selecting the highest PPI score leads the model to more easily classify host proteins as false positives. Besides, our method is computationally more efficient, requiring only 10 minutes for all 25 viruses, whereas D-SCRIPT and HBFormer require 1 hour and 5 hours for a single virus, respectively. Therefore, traditional binary PPI classification methods may not be the optimal choice for VTP prediction. The revision is provided in Lines 289-292.

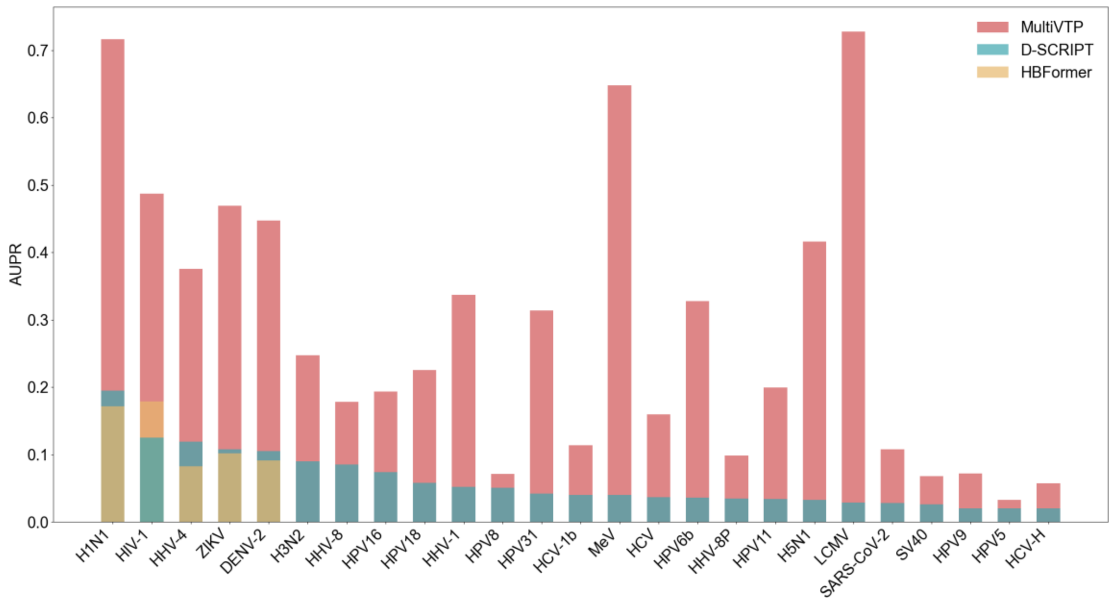

Figure R2. Performance comparison of MultiVTP with virus-host protein interaction prediction methods.

Comment 3: Briefly discuss what network topological properties you are talking about when claiming that your analysis revealed that specific network topological properties affect the interactions. Overall, you should be more specific in the whole discussions.

Response: Thank you for the suggestion. We expand our discussion on local and global network topological attributes. Viruses preferentially target host proteins having high topological centralities in the protein interaction network. Despite the effectiveness of traditional features, the topological attributes derived from node2vec balanced breadth-first and depth-first search strategies. These attributes captured both local community associations and global network positions, thereby providing a more comprehensive characterization of the network and mitigating information loss caused by subgraph sampling. For local network topological attributes, we used the shortest path distance as an attention bias, which enables the model to dynamically adjust attention weights according to the relative topological positions of nodes. The revision is provided in Lines 202-204.

Comment 4: In the clustering of VTPs and non-VTPs you used a 30% sequence identity. Explain how the degree of sequence identity was measured. For example, did you use Needleman-Wunsch alignment method?

Response: Thank you for your comment. In our study, we used CD-HIT for sequence redundancy removal instead of the Needleman-Wunsch (NW) alignment algorithm. CD-HIT is a widely used tool for large-scale sequence redundancy reduction, offering 2-3 orders of magnitude higher computational efficiency than the NW algorithm. This tool employs a greedy incremental clustering algorithm, and its alignment process

comprises two steps. The first step is fast filtering using k-mers, where each protein sequence is split into consecutive k-mer segments. If two sequences share no common k-mers, they are directly classified as dissimilar and excluded. The second step is local alignment for similar sequences. Unlike global alignment, which aligns sequences from end to end, CD-HIT identifies the optimal local segment between two sequences while allowing insertions and deletions. The sequence identity is calculated as the number of identical residues in the optimal alignment region divided by the length of the shorter sequence.

Comment 5: It is said that “we retained PPIs with confidence scores greater than 0”. In my opinion, keeping zero confidence score graph will increase the computation time of the whole method. Please provide a reason that you have to logically keep these graphs.

Response: Thanks for your comment. Regarding your concern, we provide explanations as follows. First, the HIPPIE database used to construct our PPI network includes only experimental interactions and excludes computationally predicted PPIs, ensuring that all interactions derived from this resource are reliable. Second, the computational cost of using the complete network is manageable, as the entire training process required only 5 hours on a single NVIDIA GTX 3080 Ti 12GB GPU. Finally, statistical analysis shows that most protein pairs have a confidence score above 0.6 (Figure R3A). To evaluate the impact of low-confidence data, we filtered out PPIs with scores below 0.5 and re-evaluated our model using cross-validation. The results suggested that the complete network achieved better performance, demonstrating that low-confidence interactions also contain useful information for VTP prediction (Figure R3B). The revision is provided in Lines 243-245.

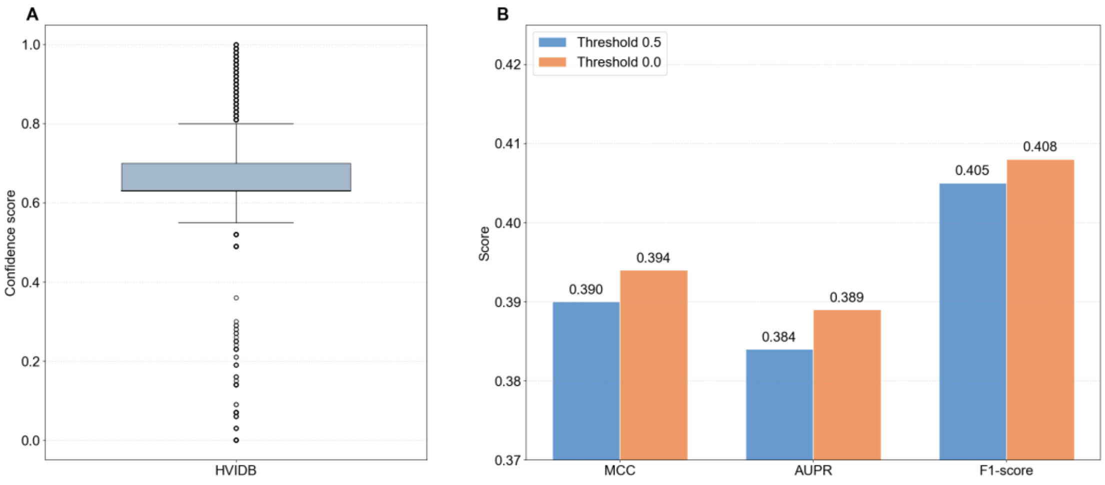

Figure R3. Evaluations of interaction confidence in HVIDB. (A) Distribution of confidence scores for protein interactions. (B) Performance comparison between interaction networks with different confidence thresholds.

Comment 6: In general, the article does not explain the reasons for various decisions in many decision-making stages, for example, and are set to 4 and 1 respectively, using

four structural attributes for each protein, adoption of ESM2 model, usage of GO database, and using the gosemsim program. It would be better if these reasons were clearly stated.

Response: Thanks for your suggestion. For global network embedding generation, we performed a grid search on the return parameter p and in-out parameter q in Node2vec. Through 5-fold cross-validation, we observed that the combination of p=4 and q=1 yielded the highest AUPR value (Figure R4). For structural information, the four properties (α-helix, β-sheet, random coil, and solvent accessibility) effectively describe the basic structural characteristics of proteins and have been widely used for predicting protein functions and protein interactions. As for sequence representation, pretrained protein language models have become the primary feature extraction method in host-virus interaction prediction. In our manuscript, we compared several popular models such as ProtTrans, ESM2, and ProteinBERT, and found that ESM2 had the best performance. Regarding protein functional information, the GO database provides systematic annotations by categorizing protein functions from three perspectives (molecular function, biological process, and cellular component), enabling the effective annotation of host proteins. For functional similarity calculation, we employed GOsemSim, a widely used tool that supports multiple similarity qualification methods. Additionally, it can not only assess protein functional similarity but also evaluate semantic relationships between GO terms, thus meeting the analytical needs of our study. The revision is provided in Lines 530.

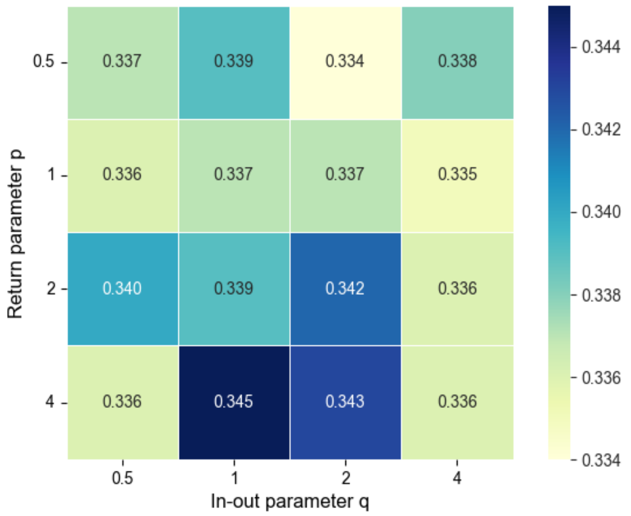

Figure R4. Impact of return and in-out parameters in node2vec on prediction performance.

Comment 7: Although many papers are cited but some closely related papers such as "Alpha influenza virus infiltration prediction using virus-human protein–protein interaction network" are not recognized. It would be good to review such papers.

Response: Thanks for your suggestion. We have cited this paper in the revised version as follows: Khorsand et al. developed a random forest model that integrated sequence composition, Gene Ontology (GO) semantic similarity, and network properties to

predict PPIs between human and H1N1. The revision is provided in Lines 75-77.

Comment 8: The implementation and evaluation section refers to many figures and diagrams that are included in the appendix of the article. However, it is not clear exactly what we have learned from this research. The article also lacks a conclusion section.

Response: Thank you for your comments. In the main text, we only displayed the most important prediction and analysis results for the VTPs of 25 viruses. In the supporting file, we provided extensive analyses as complementary information. Furthermore, we extended the above pipelines to the VTPs of 11 virus families. Similar results were observed for these virus families, further confirming the usefulness and robustness of the entire computational framework proposed in this work.

As you suggested, we add the conclusion section as follows: In this work, we presented MultiVTP, a multilabel framework for predicting VTPs by combining graph learning and multimodal data. Specifically, we generated protein embeddings by jointly capturing their network topological properties and multimodal characteristics. A graph attention mechanism and a progressive hierarchical extraction module were then introduced to integrate and refine these feature descriptors, enabling the multilabel prediction of VTPs at both the virus species and family levels. Ablation experiments confirmed the critical role of graph structural features and the designed modules in improving performance, while other components further enhance prediction accuracy. MultiVTP consistently outperformed existing VTP and PPI prediction models across diverse experimental settings, with especially notable advantages when training data were scarce. Finally, we applied MultiVTP to the human proteome to systematically identify novel host proteins associated with single or multiple viruses. The revision is provided in Lines 466-478.

Comment 9: I had difficulty finding what star symbols refer to in Figure 2.

Response: Thank you for your question. The meaning of the star symbols in Figure 2 has been clearly stated in the figure legend. The symbols denote significant differences evaluated using the Wilcoxon rank-sum test, where **** indicates $p < 0.0001$, *** indicates $0.0001 \leq p < 0.001$, ** indicates $0.001 \leq p < 0.01$, * indicates $0.01 \leq p < 0.05$, and ns indicates $p \geq 0.05$. The revision is provided in Lines 905-907.

Response to reviewer 2

1. The necessity of VTP predictions. Although the authors explained the necessity of developing VTP predictions, this prediction task appears simpler than host-virus PPI predictions. In many cases, the interacting viral protein partners are even more important for deciphering the secret of host-virus relationships. The authors may wish to comment on this issue.

Response: Thank you for this valuable comment. Host-virus PPI (HPI) prediction models leverage information from both proteins, providing more abundant information for prediction. In contrast, the VTP prediction relies only on host proteins to identify their interactions with single or multiple viruses. This is more challenging, as the models need to learn both common and virus-specific targeting patterns from the host side. This conclusion is supported by the performance of existing models in the HPI prediction field. State-of-the-art algorithms, such as HBFormer, TransPPI and STEP, achieved overall AUPRs above 0.94. For viruses like H1N1 and HIV, AUPRs remained above 0.80 (Table R1). In comparison, VTP prediction algorithms show much poorer performance. For instance, HIVPRE achieved an AUPR of 0.350 for HIV-1 VTP prediction. Our MultiVTP model, while achieving its highest AUPR of 0.723 for the LCMV virus, had an overall value of 0.375 across multiple viruses. These results confirmed the difficulty of the VTP prediction task and indicated a research gap in this field, which underscores the necessity of this study. Moreover, we selected a representative PPI algorithm, D-SCRIPT, to assess whether incorporating viral protein information could facilitate VTP prediction. Specifically, we paired each host protein in our test set with all viral proteins from the HVIDB database, and each resulting protein pair was then fed into D-SCRIPT for host-pathogen PPI prediction. For each host protein, the highest score among all pairs was used as the final VTP score. The results showed that the AUPR achieved by D-SCRIPT was considerably lower than that of our proposed model (Figure R5). This indicated that although viral protein information is crucial for host-virus interactions, HPI prediction may not be an optimal strategy for recognizing VTPs. The revision is provided in Lines 289-292.

Table R1. Performance comparison (AUPR) between VTP and HPI prediction models

Methods	Overall	H1N1	HIV	ZIKV	DENV	SARS-CoV-2
MultiVTP	0.375	0.716	0.487	0.470	0.447	0.108
HBFormer	0.992	0.849	0.976	0.558	0.683	0.336
TransPPI	0.943	0.834	0.974	0.482	0.636	0.390
LSTM-PHV	0.373	0.781	0.959	0.507	0.457	0.390
STEP	0.948	0.843	0.976	0.482	0.638	0.393
HIVPRE	NA	NA	0.350	NA	NA	NA

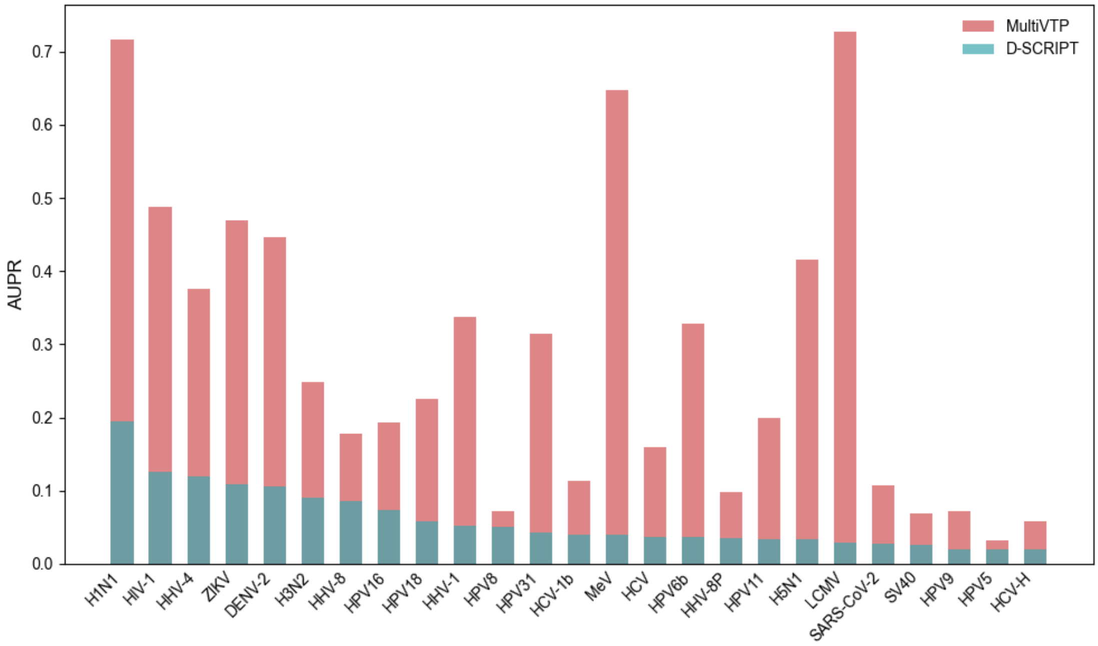

Figure R5. Comparison of MultiVTP and D-SCRIPT for VTP prediction.

2. Another common prediction task in virus bioinformatics is to predict HDFs/HRFs (host dependent factors/host restrictive factors). What is the relationship between HDFs/HRFs and VTPs?

Response: Thanks for this comment. VTPs are defined at the molecular interaction level, referring to host proteins that can directly and physically interact with viral proteins. HDFs and HRFs are defined at the functional level: the former refers to host proteins essential for the virus to complete its life cycle, while the latter refers to host proteins that inhibit viral infection and replication. More importantly, VTPs may serve as a key source of candidates for HDFs/HRFs. Most experimentally validated HDFs/HRFs exert their functions by interacting with viral proteins. Following the HIVPRE method, we collected 510 HRFs and 1314 HDFs from the HIV-1 human interaction database, and all of these HDFs/HRFs are known VTPs (Figure R6). Therefore, VTP predictions

could provide a new avenue for the discovery of HDFs/HRFs. The revision is provided in Lines 433-439.

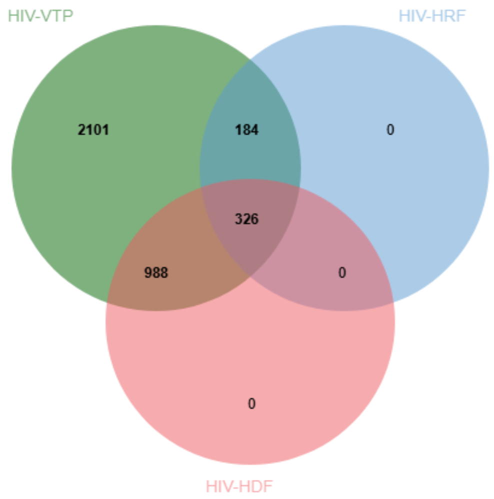

Figure R6. Venn diagram of HIV-related host proteins.

3. The non-VTPs are also a very important concept in this work. Therefore, the authors should provide a serious definition.

Response: Thanks for this comment. Non-VTPs are defined as host proteins for which no experimentally confirmed interaction with any virus has been reported in the HVIDB database. This definition has been added into the revised manuscript. The revision is provided in Lines 485-486.

4.The manuscript defines host proteins without reported virus interactions in the HVIDB database as non-VTPs. However, this assumption does not adequately account for the severe incompleteness and reporting bias inherent in virus–host interaction databases. Many proteins labeled as non-VTPs may simply represent uncharacterized or insufficiently studied virus targets rather than true negatives. Moreover, HVIDB is not the only available resource for virus–host protein interactions. Other widely used databases, such as IntAct and BioGRID, also curate experimentally supported and predicted virus–host interactions. Restricting the definition of VTPs and non-VTPs to a single database may further amplify label noise and dataset bias.

Response: Thanks for this useful comment. We completely agree with your viewpoint. Therefore, we expanded the original dataset by incorporating the other two virus-host protein interaction databases, IntAct and BioGRID, and updated the labels of samples in this work. The number of newly added VTPs was most notable for HPV16, H5N1, HHV-8P, and SARS-CoV-2. Notably, the number of VTPs for SARS-CoV-2 increased by 2,363, reflecting the recent surge in research on COVID-19 virus-host interactions. For the other viruses included in this study, the number of newly identified VTPs was relatively limited, confirming the completeness of HVIDB for these viruses (Figure R7A). In addition, we retrained the model using the updated dataset and evaluated its

performance on the newly annotated test set. The model showed only limited AUPR improvement for most viruses, while relatively substantial gains were observed for those with a large number of newly added VTPs. Notably, the AUPR of our original model was merely 0.108 on the uncorrected SARS-CoV-2 test set. In contrast, its AUPR increased to 0.814 when evaluated on the updated test set (Figure R7B). This result indicates that our model can effectively identify potential VTPs, even when trained on samples containing a high proportion of false negatives. The revision is provided in Lines 427-433.

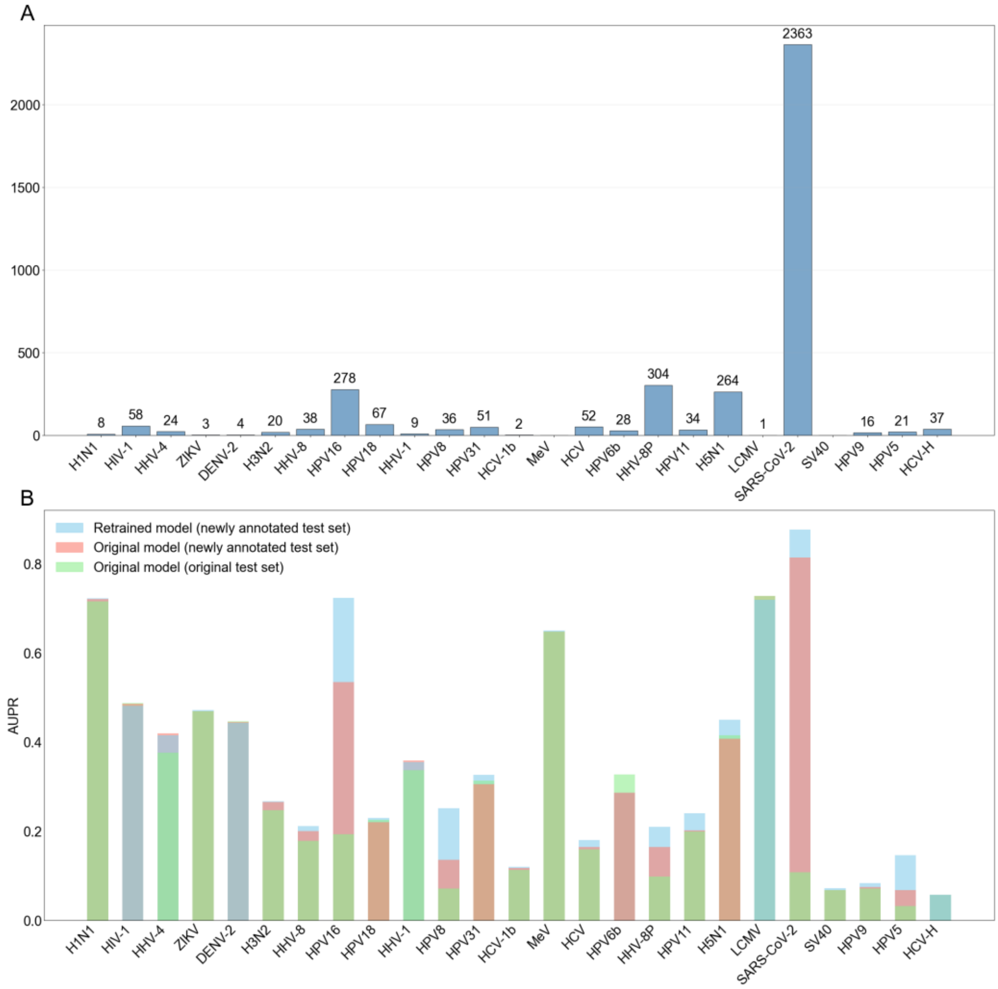

Figure R7. Impact of database integration on VTP prediction. (A) Number of newly added VTPs for each virus. (B) Performance comparison of models trained on different data sources.

5. In the section "Datasets", the authors mention that "To mitigate the imbalance of positive and negative samples, the virus having less than 200 VTPs was excluded from this study". However, later in the Results section, the authors claim that: "These results implied the potential of our model in few-shot learning tasks". Excluding viruses with fewer than 200 known targets introduces a strong selection bias toward well-studied viruses and directly contradicts the claim that the model is suitable for few-shot scenarios. Viruses with limited known targets are removed from the main dataset, yet the model is later presented as effective under limited-data conditions.

Response: Thank you for your insightful comment. We apologize for the ambiguity in our original manuscript, which may have caused confusion. Viruses with fewer than 200 known VTPs were excluded only from the training process of our main model, not from the entire study. This was to ensure stable learning of general interaction features and to avoid training instability caused by scarce positive samples for certain viruses. To evaluate the model's few-shot learning ability, we conducted two experiments in the original manuscript: (1) assessing model performance by randomly removing VTPs from the training set, and (2) transferring the pre-trained model to novel viruses with 20-100 VTPs. The results demonstrated the model's potential for few-shot learning applications.

In the revised version, we conducted more experiments to validate this point. First, we simulated scenarios with extremely scarce labeled data by progressively removing VTPs for each virus in the training set at a high ratio. The model was then evaluated on the test set. All experiments were repeated five times to reduce fluctuations caused by random sampling under limited data. The results showed that our model maintained stable AUPR values even after removing 90% of known VTPs, while performance dropped sharply when 99% of VTPs were eliminated (Figure R8A). These results confirm that the model remains effective under scarce labeled data conditions. Second, we performed leave-one-virus-out validation on five randomly selected viruses. Specifically, the model was pre-trained on a training set that excluded VTPs of the target virus. Because the pre-trained model lacked an output channel for the new virus, we introduced a virus-specific expert and prediction head, which were then fine-tuned using only 20 targets from that virus. The model was then evaluated on the test set, with all experiments repeated five times. Compared with the original training scheme, this strategy yielded reduced but still effective performance across the five viruses, verifying the model's generalization ability to unseen viruses (Figure R8B). The revision is provided in Lines 329-332.

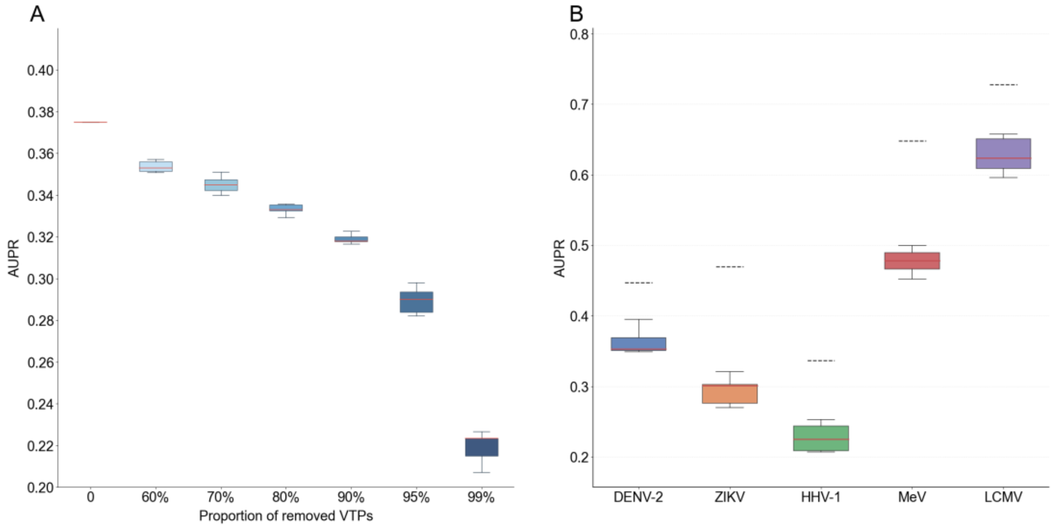

Figure R8. Robustness of MultiVTP with limited training data. (A) Performance of MultiVTP after removing different fractions of training VTPs. (B) Performance of leave-one-virus-out validation.

6. This inconsistency between dataset design and claimed application scope needs to be

addressed. A clearer justification for the chosen threshold, or a sensitivity analysis demonstrating how model performance changes with different minimum VTP counts, would significantly strengthen the study.

Response: Thank you for your suggestion. To address this issue, we added a sensitivity analysis as you suggested. Specifically, we tested four minimum VTP count thresholds (i.e., 100, 200, 300, and 400) based on 5-fold cross-validation. The results showed that the model maintained stable prediction performance for the corresponding viruses when the threshold was set to 200, 300, or 400. However, when the threshold was set to 100, the performance clearly declined for most viruses (Figure R9). This may be attributed to the extreme imbalance between positive and negative samples, as well as the seesaw phenomenon and negative transfer in multi-task learning. Based on the results, we ultimately selected 200 as the minimum VTP count threshold. The revision is provided in Lines 498-499.

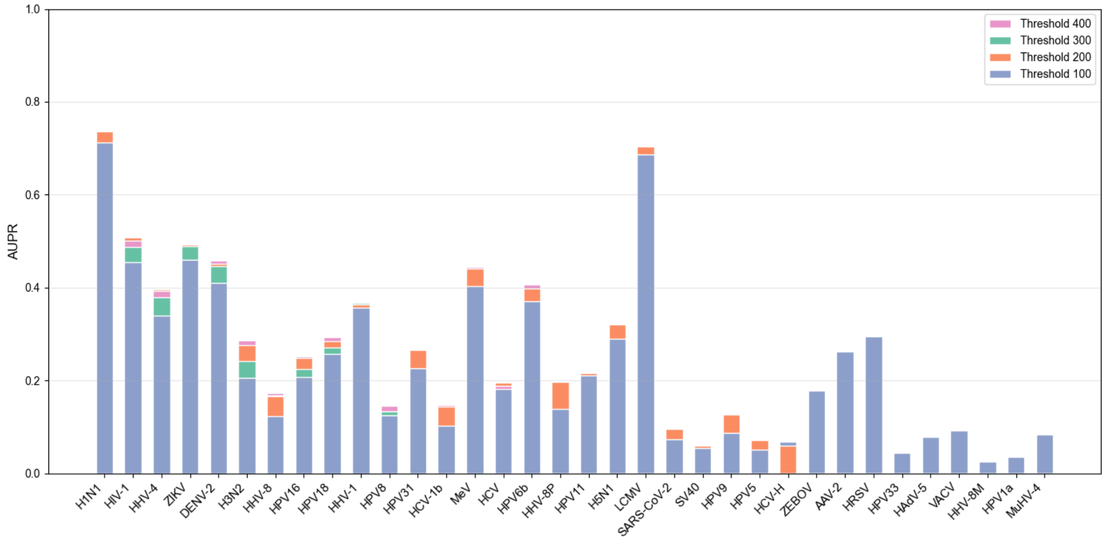

Figure R9. Model performance based on different minimum VTP count thresholds.

7. The baseline comparisons in the manuscript are primarily limited to traditional machine learning models (e.g., XGBoost, SVM, Random Forest, and MLP) combined with standard multilabel learning strategies. While these comparisons are informative, they do not include recent state-of-the-art deep learning models for host–virus interaction or VTP prediction methods. Notably, several transformer- or GNN-based methods that leverage protein language models and network information are discussed in the Introduction, yet none of these approaches are included as baselines in the experimental evaluation.

Response: Thank you for the valuable suggestion. We selected two representative deep learning models as supplementary baselines, namely D-SCRIPT and HBFormer. D-SCRIPT is a sequence-based PPI prediction model with strong generalization in cross-species interaction prediction, whereas HBFormer uses a hybrid attention transformer combined with multimodal features for host-virus PPI prediction. Because these models

were originally designed for binary PPI predictions, we adjusted the prediction strategy to ensure a fair comparison. Specifically, each host protein was paired with all viral proteins in the HVIDB database, and the highest score among all PPI predictions was used as the prediction score for the host protein. Considering the high computational cost of this pairing strategy, D-SCRIPT was evaluated on all 25 viruses due to its higher efficiency, whereas HBFormer was only tested on the five viruses with the highest VTP counts owing to its lower efficiency. The results suggested that MultiVTP remarkably outperformed the baseline models in terms of AUPR across all viruses (Figure R10). This may be because selecting the highest PPI score leads the model to more easily classify host proteins as false positives. Therefore, traditional binary PPI classification methods are not suitable for VTP prediction. The revision is provided in Lines 289-292.

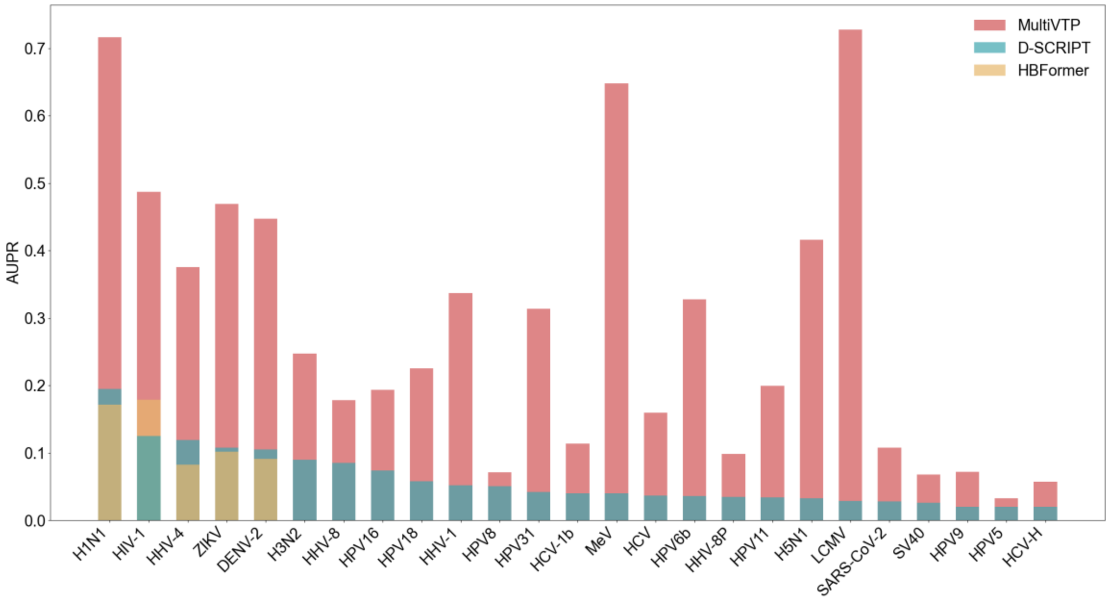

Figure R10. Performance comparison of MultiVTP and PPI prediction models for VTP identification.

8. The manuscript presents attention score distributions, t-SNE visualizations, and functional enrichment analyses to support model interpretability. However, it remains unclear how these analyses translate into concrete mechanistic insights. For example, while VTPs are shown to receive higher attention scores, it is not demonstrated whether these attention patterns correspond to specific biological functions, network motifs, or known host–virus interaction mechanisms. Additional analyses linking attention weights or learned representations to interpretable biological structures would strengthen the interpretability claims.

Response: Thanks for your suggestion. In the present study, we found that our model assigned higher attention scores to VTPs. These proteins were enriched in biological processes closely associated with viral infection, including protein transport, apoptosis, transcriptional regulation, and antiviral defense. To further associate attention weights with interpretable biological functions, we extracted functional regulatory relationships between host proteins from the Signor database and classified them into activation and

inhibition interactions. By comparing attention scores, we observed higher values for protein pairs involved in such regulatory relationships, suggesting that our model could effectively capture key regulatory mechanisms (Figure R11A). The heightened attention toward VTPs and regulatory interactions may reflect the molecular perturbations induced during viral infection. For instance, in the subgraph centered on BCL2A1, high attention scores were detected among BCL2A1, BAX, and BRD4. Previous studies have verified that the viral protein E2 binds to BRD4 during HPV infection, promoting the activation of BCL2A1, which in turn enhances inhibition of the downstream protein BAX. This cascade suppresses apoptosis in infected cells and creates a favorable environment for viral replication (PMID: 34386523). Another high-attention protein pair, KLHDC10 and RBX1, also represents a strong regulatory interaction. Upon recognition of viral proteins by KLHDC10, this protein recruits RBX1 to amplify the apoptotic signal, thereby eliminating infected cells and degrading viral replication factors (PMID: 39887712) (Figure R11B). The revision is provided in Lines 262-265.

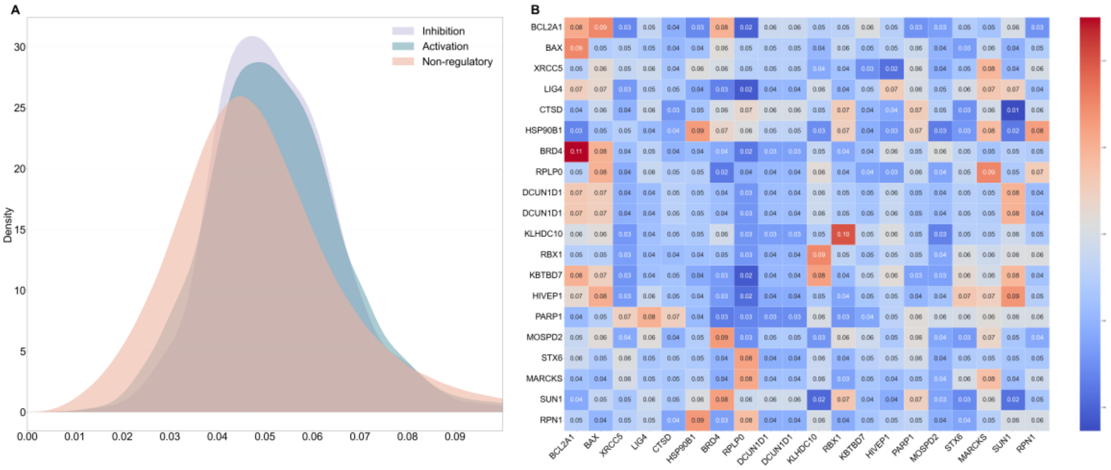

Figure R11. Linking attention score analysis. (A) Density distribution of attention values of protein pairs across regulatory interaction types. (B) Attention scores within BCL2A1-centered subgraph.

9. While the authors provide source code and model implementations, the manuscript lacks essential details regarding computational resources and training procedures. Information such as hardware configuration (e.g., GPU type and memory), training time, and scalability with respect to network size or number of viruses is not reported.

Response: Thank you for your comment. All models in this study were trained under a unified hardware and software environment. The hardware configuration included an NVIDIA GTX 3080Ti 12GB GPU, paired with an Intel Xeon Silver 4116T CPU and 187GB of RAM. The workflow was implemented using the PyTorch 2.2 framework and accelerated by CUDA 11.4. During the training process, we adopted the Adam optimizer combined with a linear learning rate scheduler incorporating a warm-up phase, with the initial learning rate set to 5e-4 and a batch size of 64. This setup enabled the models to converge after training on all 25 viruses in approximately 5 hours. Theoretically, the model can be scaled to protein-protein interaction networks of any

size and any number of virus types, provided that sufficient computational resources are available. For emerging viruses, we appended a virus-specific expert module to the output layer of Graphormer. Its representations were fused with those of pre-trained shared experts, and new output heads were introduced for each novel virus. In the fine-tuning process, a differential learning rate strategy was adopted: 1e-4 for the general backbone and 5e-4 for the virus-specific module, allowing adaptation to an unseen virus in approximately 10 minutes. The revision is provided in Lines 651-653.

10. According to the current model architecture, can the proposed method be used for predicting VTPs for newly emerging viruses?

Response: Thanks for your comment. Our model is applicable to the prediction of VTPs for newly emerging viruses. In the manuscript, we have adapted the pre-trained model to emerging viruses with VTP counts ranging from 20 to 100 using scratch training and fine-tuning strategies. To further examine emerging viruses with extremely limited data, we constructed a more challenging few-shot dataset consisting of 15 novel viruses (each with 5-17 VTPs), ensuring that none of these samples appeared in the training set (Figure R12A). This dataset contained 117 VTPs and 1,372 non-VTPs, and all experiments were evaluated using stratified 5-fold cross-validation. As a result, models trained from scratch suffered from performance collapse (AUPR < 0.1), while the fine-tuning strategy yielded better performance, with AUPRs ranging from 0.1 to 0.7 for most viruses. Relatively poor performance was observed for the remaining viruses (e.g., HPV3, HPV16, H7N7, and H9N2), probably because some targets are SVTPs that lack cross-virus generalizable patterns, which remains a challenge for the current model (Figure R12B). Collectively, our model is suitable for newly emerging viruses. The revision is provided in Lines 329-332.

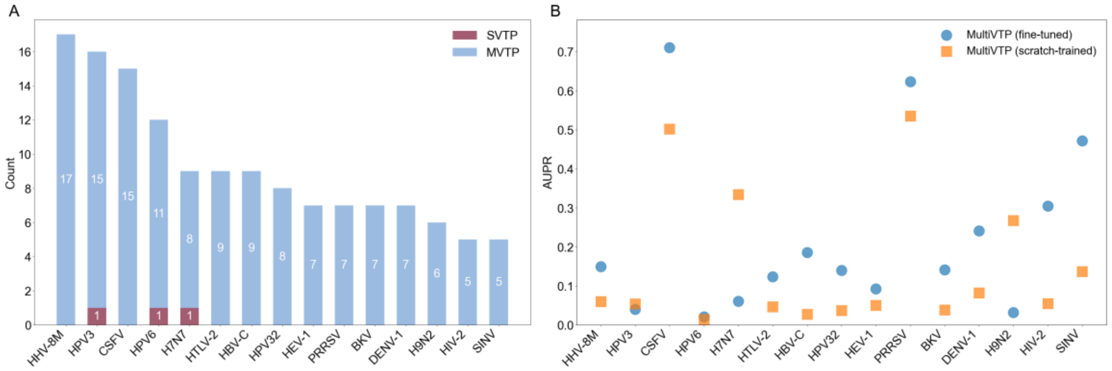

Figure R12. Evaluation of MultiVTP on understudied viruses. (A) Distribution of VTP counts across viruses in the few-shot dataset. (B) Performance of our model on understudied viruses.

11. The analysis should follow the basic principle of biochemistry and human-virus biology. For example, the authors stated that “VTPs had more α-helices and fewer coils than non-VTPs, reflecting their potential structural specificity for interacting with the virus”. To prove this statement is reasonable, could the authors provide more evidence?

Response: Thank you for this comment. The enrichment of α-helices in VTPs can be

explained from several perspectives. First, α-helices are common recognition elements in protein-protein interactions, and their stable interfaces facilitate viral protein binding. For example, the α-helical domain of ALIX provides a specific binding pocket for retroviruses, directly modulating their binding efficiency (PMID: 17350572). Second, because α-helices are frequently located in functionally critical regions of host proteins, viral targeting of these structures can efficiently disrupt cellular processes. For instance, the influenza A virus NS1 protein inserts into the α-helical domain of host TRIM25, thereby effectively inhibiting antiviral signaling. (PMID: 19454348). Moreover, α-helices in host proteins are often highly conserved, rendering them common interaction targets for diverse viral proteins. For example, influenza virus NS1, Ebola virus VP35, and SARS-CoV-2 NSP13 all bind directly to the α4 helix of IRF3, thereby blocking IRF3 activation and subsequent innate immune responses. (PMID: 10933707, 12829834, 37314155). Regarding the lower coil content in VTPs, this is probably because such proteins are important functional proteins in cells, which are structurally conserved and adopt well-folded conformations. Viruses tend to target conformationally stable and interface-conserved host domains for specific binding, whereas highly flexible and disordered regions are unfavorable for stable and efficient virus-host interactions. (PMID: 24184279). The revision is provided in Lines 163-165.

12. The prediction results showed that influenza A (H1N1) and HIV-1 had the highest number of candidates (2,555 and 1,681, respectively). Given the size of the human proteome, the predicted VTP number appears unexpectedly large (more than 10%). The authors may wish to comment on this issue.

Response: Thank you very much. The predicted number of VTP candidates appears unexpectedly large because these two viruses inherently possess a greater number of known VTPs. When applying MultiVTP to the human proteome, the threshold for each virus was selected based on the actual distribution of known VTPs (Figure R13). This ensures that the predicted candidates reflect biologically relevant priors and reduces bias that could arise from using arbitrary thresholds. The higher counts for H1N1 and HIV-1 primarily stem from their nature as RNA viruses with limited coding capacity, which extensively hijack host proteins for replication. H1N1's long-term circulation with multiple variants diversifies its targets, while HIV-1's chronic infection necessitates complex regulation of host pathways, engaging numerous host proteins at each step of its life cycle.

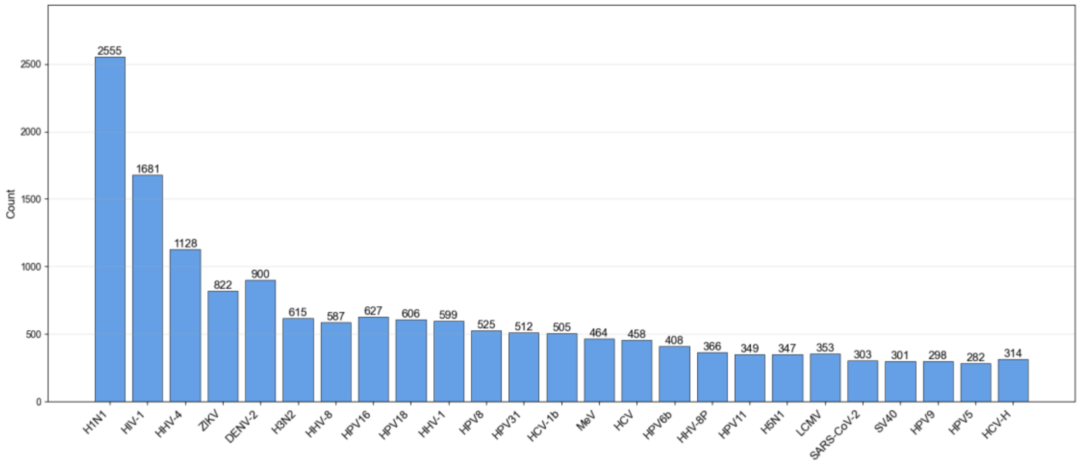

Figure R13. Number of known VTPs included in HVIDB across 25 viruses.

13. Regarding the functional analysis of predicted VTPs, the authors may conduct a comparison of VTPs and HDFs (the HDFs for some viruses are known). Such a comparison may provide new hints to indirectly validate the prediction results.

Response: Thank you for your comment. After collecting the host dependency factors (HDFs) of HIV-1, we performed functional enrichment analysis using the DAVID database. Figure R14 shows the overlap of enriched terms between HDFs and viral-targeted protein (VTP) candidates, suggesting that these two types of host proteins were highly associated during the infection process (Figure R14A). We then ranked the biological process terms of HDFs based on their enrichment significance. Among the top 20 terms, HDFs were associated with core infection-related processes, such as defense response to virus, immune and inflammatory regulation, regulation of apoptosis, and regulation of gene expression. Notably, VTP candidates were also enriched in 15 out of these 20 key terms (Figure R14B). Moreover, we calculated the recovery rate (overlap ratio) of VTP candidates for the top k terms. For terms within the top 50, the recovery rate of VTP candidates exceeded 70%. Even for the top 100 terms, the recovery rate remained stable at approximately 60%, and it only dropped below 50% for terms beyond the top 200 (Figure R14C). These indicated that the predicted VTPs were biologically reliable and closely related to known infectious functions. Subsequently, we focused on the term ‘regulation of autophagy’, which was enriched in both VTP candidates and HDFs. Among the 12 candidates, 8 corresponded to known VTPs, and an additional 3 were supported by literature mining. Notably, USP10 (UniProt ID: Q14694) and MTOR (UniProt ID: P42345) were identified as HDFs (PMID: 34983926, 32579936). Moreover, MTOR has been validated as a key regulator of autophagy during ZIKV and HCMV infections, implying that it may exert a similar function during HIV-1 infection (Figure R14D) (PMID: 24108761). The revision is provided in Lines 373-377.

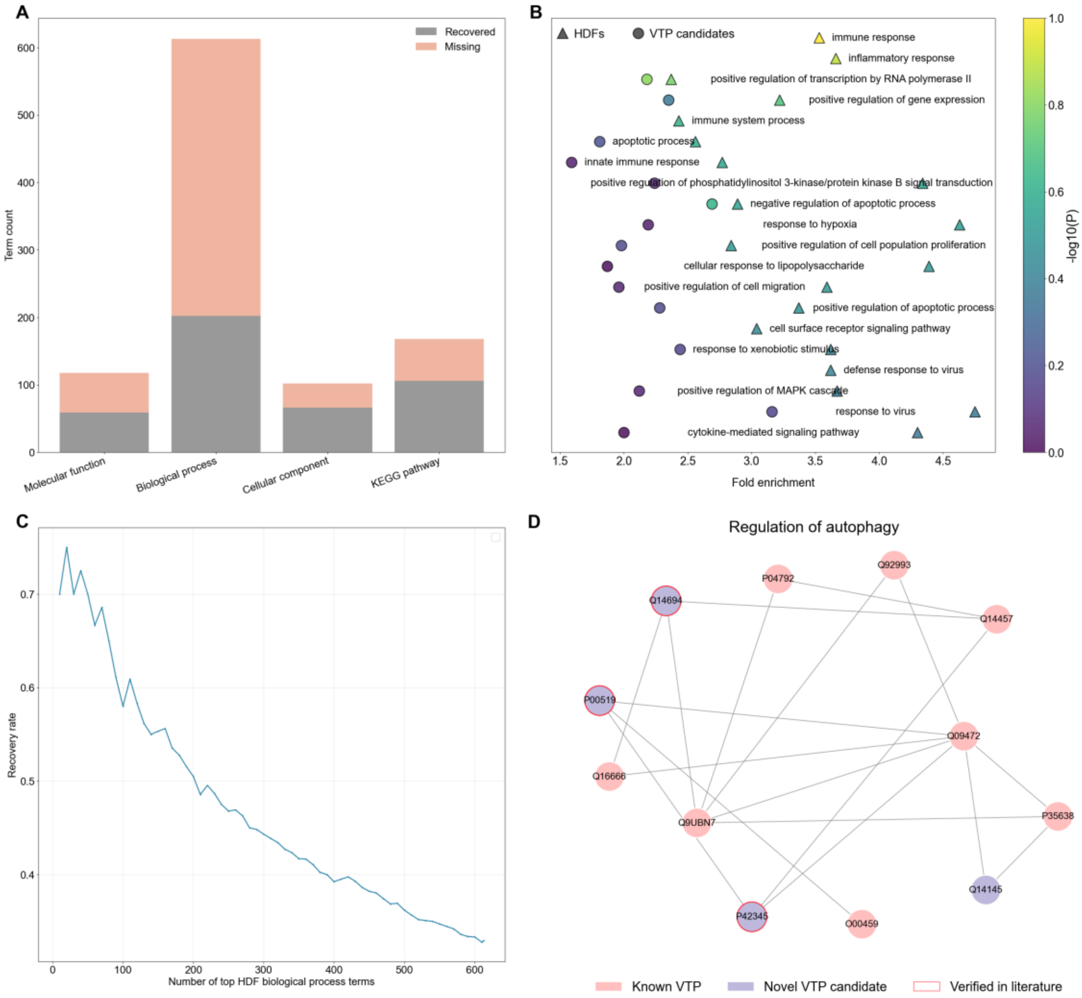

Figure R14. Analysis of VTP candidates and known HDFs in HIV-1 infection. (A) Distribution of functional terms enriched in predicted VTPs and HDFs. The results are classified into two categories: recovered (overlapping terms between VTP candidates and HDFs), and missing (terms enriched exclusively in HDFs). (B) Top 20 biological processes enriched in HDFs. (C) Recovery rate of VTP candidates for the top k terms enriched in HDFs. (D) Interaction network of VTPs related to the 'regulation of autophagy' term.

Response to reviewer 3

1. Scope and framing of the Introduction
The Introduction is generally well written but historically narrow and heavily method-focused. It would benefit from stronger grounding in field-defining experimental host–virus interactome studies.
Key examples that should be discussed include:
Jäger et al., (2011) — https://pubmed.ncbi.nlm.nih.gov/20708689/
Gordon et al., (2020) — https://pubmed.ncbi.nlm.nih.gov/32353859/
Stukalov et al., (2021) — https://pubmed.ncbi.nlm.nih.gov/33845483/
These foundational datasets underpin much of the training data used in computational host–virus PPI prediction and would better contextualise the importance of the problem.

Response: Thank you for your suggestion. We have rephrased the first paragraph in the introduction section and cited the three mentioned papers as follows: Human-virus protein-protein interactions (PPIs) are involved in various biological processes that viruses hijack for their replication. Virus target proteins (VTPs) are host proteins specifically recognized and bound by viral components. In host cells, these proteins not only participate in immune regulation but also orchestrate multiple stages of the viral life cycle. For instance, the human protein angiomotin promotes human immunodeficiency virus 1 (HIV-1) budding by bridging the viral Gag protein and the host protein NEDD4L. Identifying VTPs is essential for elucidating the molecular mechanisms of viral pathogenesis and provides promising avenues for antiviral drug development. Leveraging experimental techniques such as yeast two-hybrid assays (Y2H) and affinity purification mass spectrometry (AP-MS), a great number of virus-host protein interactions have been characterized. For example, Jäger et al. constructed a high-quality HIV-human protein interactome using AP-MS. Shapira et al. identified 1,745 host factors involved in influenza virus replication through Y2H. Gordon et al. generated a global protein interaction map of SARS-CoV-2 using AP-MS, while Stukalov et al. profiled the interactomes of SARS-CoV-2 and SARS-CoV to perform a multi-omics analysis. Collectively, these studies provide critical experimental datasets for developing computational methods to identify VTPs. The revision is provided in Lines 57-65.

2. Protein language models and foundation models
The manuscript does not adequately cover recent advances in protein language models (PLMs) and foundation models for interaction prediction.
For example:
Liu et al (2025) PLM-interact: Extending protein language models to predict protein–protein interactions - https://pubmed.ncbi.nlm.nih.gov/41145424/
This and related studies represent an important methodological shift and should be incorporated into the discussion of state-of-the-art approaches.

Response: Thank you for your suggestion. The mentioned PLM is suitable for a protein

pair rather than a single protein. Instead, we have included the latest ESM C model as a baseline method for comparison. However, cross-validation results indicated that ESM C did not yield a marked performance improvement on this task (Figure R15A). For the state-of-the-art approaches, we selected two representative deep learning models as supplementary baselines, namely D-SCRIPT and HBFormer. D-SCRIPT is a sequence-based PPI prediction model with strong generalization in cross-species interaction prediction, whereas HBFormer uses a hybrid attention transformer combined with multimodal features for host-virus PPI prediction. Because these models were originally designed for binary PPI predictions, we adjusted the prediction strategy to ensure a fair comparison. Specifically, each host protein was paired with all viral proteins in the HVIDB database, and the highest score among all PPI predictions was used as the prediction score for the host protein. Considering the high computational cost of this pairing strategy, D-SCRIPT was evaluated on all 25 viruses due to its higher efficiency, whereas HBFormer was only tested on the five viruses with the highest VTP counts owing to its lower efficiency. The results suggested that MultiVTP remarkably outperformed the baseline models in terms of AUPR across all viruses (Figure R15B). This may be because selecting the highest PPI score leads the model to more easily classify host proteins as false positives. Therefore, traditional binary PPI classification methods are not suitable for VTP prediction. The revision is provided in Lines 289-292.

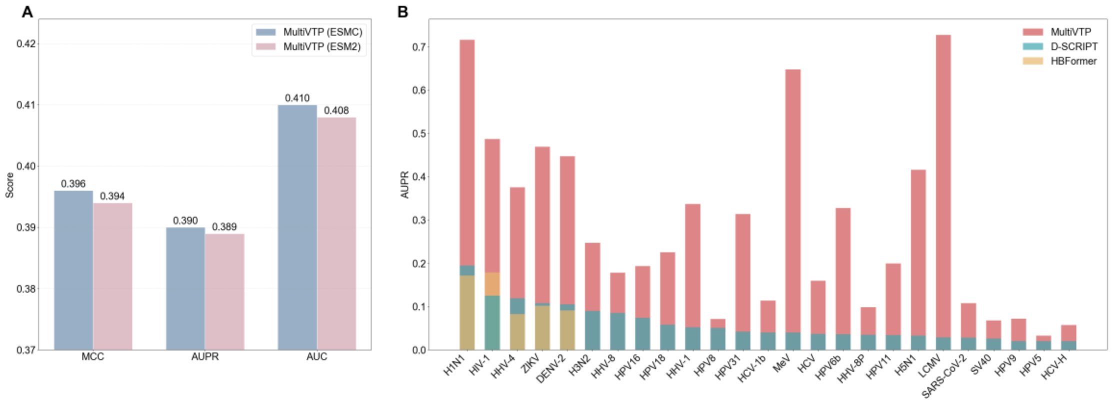

Figure R15. Performance comparison between MultiVTP and state-of-the-art approaches. (A) Performance comparison of ESM2 and ESMC. (B) Performance comparison of MultiVTP and PPI prediction models for VTP identification.

3. Directionality of virus–host interactions

The manuscript treats virus target proteins largely as a single functional class. However, host–virus interactions are biologically heterogeneous and include:

Pro-viral (host dependency factors)

Anti-viral (restriction factors)

The distinction is important both mechanistically and therapeutically. The authors already cite Chai et al. (PMID: 35134057) but do not introduce the complexity of the virus target proteins.

The authors should also discuss whether their framework can distinguish — or could be extended to distinguish — interaction directionality.

Response: Thank you very much for your comment. The host proteins associated with viral infections could be divided into HDFs and HRFs according to their functions. Specifically, HDFs are host proteins essential for the virus to complete its life cycle, whereas HRFs function to inhibit viral infection and replication. To extend MultiVTP for interaction directionality prediction, we first annotated HIV-1 VTPs based on Chai et al.'s work and categorized them into forward (HDFs) and backward (HRFs) types. We then expanded our model's output for HIV-1 into two dedicated channels, each corresponding to one directionality type. Results show that the two types of directional predictions achieved comparable performance, and the superior performance of HDF prediction could be attributed to the larger number of training samples for HDFs (695 samples) compared with HRFs (only 290 samples) (Figure R16). The revision is provided in Lines 433-439.

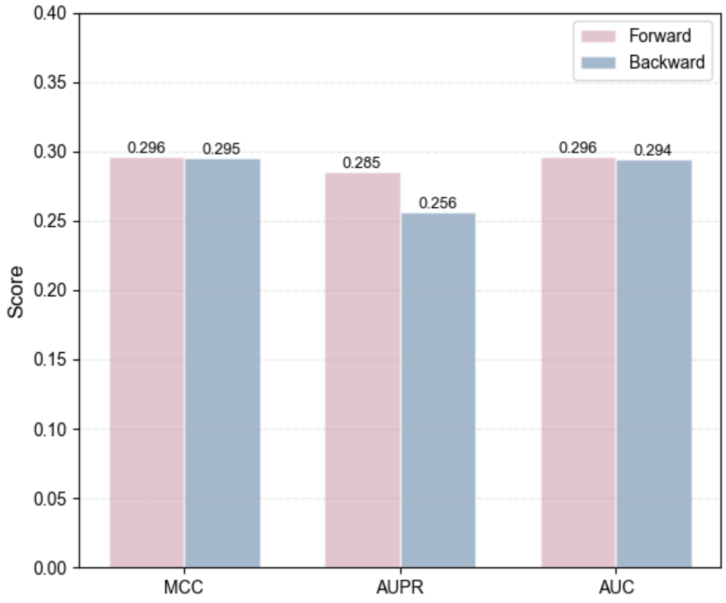

Figure R16. Performance of MultiVTP for interaction directionality prediction.

4. Methodological considerations: negative sampling

A key issue in PPI prediction that is not discussed is the construction of negative datasets.

Common challenges include:

Lack of experimentally validated true negative PPIs

Reliance on random protein pairing

Bias introduced into performance metrics

A good starting reference would be:

Ben-Hur & Noble, Bioinformatics (2006) — On negative examples in PPI prediction

This limitation should be acknowledged in the Methods or Discussion.

Response: Thanks for your suggestion. To evaluate the effect induced by the quality of non-VTPs, we adopted two strategies to construct the training set. The first strategy utilizes the positive-unlabeled learning method with a spy technique. We randomly selected 15% of VTPs as 'spy samples' and combined them with non-VTPs to train an

XGB classifier. We then set the lowest score covering 95% of the spy samples as the threshold, and considered non-VTPs with a score below this threshold as reliable negative samples. We retrained our model with this refined training set and evaluated its performance on test sets. The results showed that all metrics except recall decreased. This is likely because while the strategy successfully removes false negative samples, the selected non-VTPs differ markedly from VTPs in terms of their features. As a result, the model fails to adequately learn from 'difficult negative samples', and thus struggles to accurately predict non-VTPs that resemble genuine VTPs. For the second strategy, we performed functional enrichment analysis on VTPs and selected GO terms and KEGG pathways with a p-value < 0.05 and fold enrichment > 2 as more conserved functions for viral infection. Non-VTPs not annotated to these terms were then regarded as reliable non-VTPs. The results indicated that the MCC, recall, precision, and F1 measures increased, while the AUPR measure remained stable (Table R2). This could be attributed to the higher quality of negative samples and the reduction of label noise. Altogether, our analyses demonstrate that the initial non-VTP selection scheme reliably reflects the predictive power of our model. The revision is provided in Lines 427-433.

Table R2 Performance comparison of different negative sampling strategies

Negative sampling strategy	D_{virus}					
	MCC	AUPR	AUC	RC	F1	Pre
MultiVTP	0.347	0.375	0.839	0.433	0.370	0.390
PU_spy	0.323	0.362	0.830	0.558	0.336	0.314
Not in BP	0.372	0.376	0.840	0.482	0.390	0.408
Not in CC	0.373	0.375	0.840	0.507	0.390	0.395
Not in KEGG	0.376	0.375	0.841	0.482	0.393	0.421
Not in any conserved terms	0.372	0.371	0.838	0.469	0.390	0.417

5. Acronyms and terminology

Several acronyms are not defined at first use. For clarity:

GCN — Graph Convolutional Network

GAT — Graph Attention Network

Please define all abbreviations when first introduced.

Response: Thank you for your suggestion. We have defined all abbreviations at their first mention in the revised manuscript. The revision is provided in Lines 247-248.

6. Cross-virus interaction overlap

The manuscript states:

"This result indicated that MultiVTP could better capture cross-virus interaction patterns."

However, the extent of shared host targeting between viruses is not quantified.
It would strengthen the claim to report, for example:
Number of shared host proteins between virus pairs
Overlap coefficients / Jaccard indices

Response: Thanks for this comment. Following your suggestion, we have quantitatively measured the shared host proteins between viruses using Jaccard indices in the dataset. The results indicated that viruses such as HIV-1 and H1N1 exhibit a higher Jaccard index, while viruses associated with HHV share similar targeting patterns (Figure R17A). To investigate the performance discrepancy between SVTPs and MVTPs, we re-evaluated our model on these two groups separately using 5-fold cross-validation. Our results showed that the model achieved lower measures for SVTPs than for MVTPs. This is likely because SVTPs are mainly governed by virus-specific molecular patterns, which are difficult to infer from host information alone (Figure R17B). In contrast, MVTPs exhibit more conserved interaction patterns and are thus easier to predict correctly. The revision is provided in Lines 382-386.

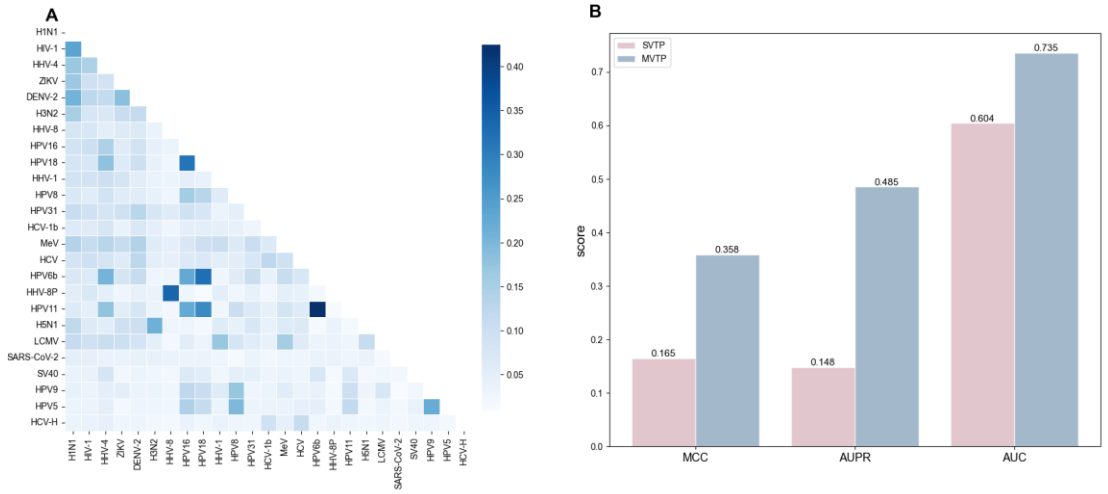

Figure R17. Evaluation of our model using overlapping and virus-specific VTPs. (A) Overlap of VTPs among different viruses by Jaccard indices. (B) Performance of our model on SVTPs and MVTPs.

7. Figures and visualisation clarity
Several figures would benefit from improved labelling and legend placement.
General comment:
In many cases, colour legends or numeric virus identifiers are used where direct axis labelling with virus names would be clearer.
Specific suggestions:
Figure S1 — Difficult to interpret without x-axis labels
Figure S2 — Please add virus names on both x- and y-axes
Figure 2 (A–D) — Improve axis labelling clarity
Figure S8 — Clear and easy to interpret (good example)
Figure S5 — Current format acceptable given long family names
Improved labelling would substantially enhance accessibility.

Response: We greatly appreciate your kind suggestion. Considering the large number of virus types and their lengthy names, directly displaying the full virus names on the axes would result in severe overcrowding and reduced overall readability. Therefore, we retain the current labeling and legend design.

8. Discussion of limitations

The Discussion should be expanded to address key limitations, including:

Inability to distinguish pro-viral vs anti-viral targeting

Potential biases in negative interactions

Virus coverage and generalizability

Response: Thank you very much. We performed the relevant experiments as follows:

Inability to distinguish pro-viral vs anti-viral targeting: see Response 6.

Potential biases in negative interactions: see Response 4.

Virus coverage and generalizability:

To examine whether MultiVTP could be applied to newly emerging viruses, we extended the original model by incorporating virus-specific expert modules and output channels, followed by transfer learning. In addition, we constructed a challenging few-shot dataset consisting of 15 novel viruses (each with 5-17 VTPs), with none of these samples appearing in the training set (Figure R17A). Under this setting, models trained from scratch suffered from severe performance collapse (AUPR < 0.1). In contrast, our fine-tuning strategy achieved clearly better performance, with AUPR values ranging from 0.1 to 0.7 for most viruses(Figure R17B). Relatively poor performance was observed for the remaining viruses (e.g., HPV3, HPV16, H7N7, and H9N2), likely because some of their targets are SVTPs that lack cross-virus generalizable patterns, which remains a challenge for the current model. Collectively, our model is suitable for understudied or newly emerging viruses, demonstrating its potential generalizability. The revision is provided in Lines 329-332.

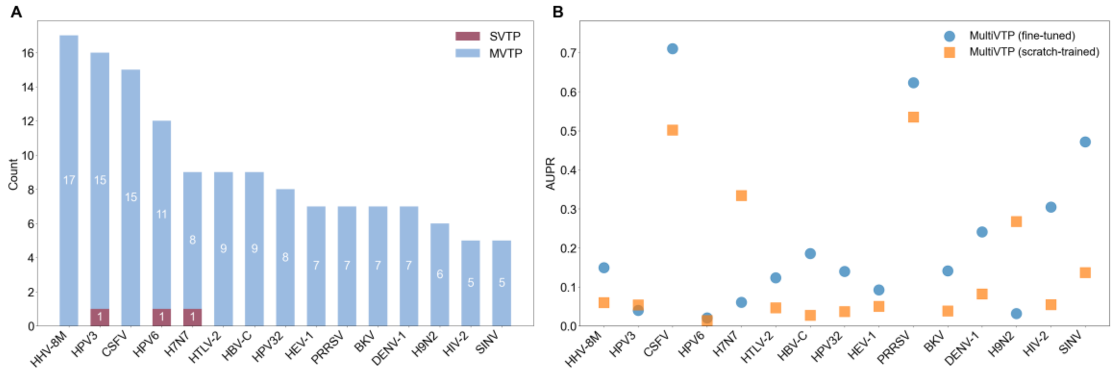

Figure R18. Evaluation of MultiVTP on understudied viruses. (A) Distribution of VTP counts across viruses in the few-shot dataset. (B) Performance of our model on understudied viruses.

Response to reviewer 4

1. The central premise of this study is the reformulation of virus target prediction as a multilabel task relying exclusively on host protein information. While methodologically appealing, this assumption requires substantially stronger biological justification. In many viral systems, targeting specificity is primarily governed by viral proteins that determine binding interfaces and interaction selectivity. By excluding viral determinants entirely, the proposed task may be closer to estimating host susceptibility rather than predicting genuine host–virus interactions. The authors should more clearly define the biological question their model is capable of addressing; otherwise, a mismatch may exist between the computational objective and the underlying molecular mechanisms. It is particularly important to clarify under which categories of host–virus interactions this assumption is expected to hold, and where it is likely to fail, especially for interactions mediated by virus-specific structural domains or accessory proteins.

Response: Thank you for your valuable comment. In our study, we hypothesized that statistically significant biological differences exist between VTPs and non-VTPs, as well as among VTPs from different viruses, and that these differences can be captured by our model. To validate this hypothesis, we performed a systematic comparative analysis and found that VTPs tend to have a higher proportion of negatively charged amino acids, exhibit stronger evolutionary conservation, are enriched in regular protein secondary structures, and occupy topological central positions in the protein interaction network. Furthermore, we performed functional enrichment analysis for the VTPs of each virus and selected the top 100 terms as functional hotspots for each virus. We then calculated the overlap of these hotspot terms across different viruses. As shown in the heatmap, although different viruses share certain overlapping functions (e.g., essential host pathways commonly hijacked by viruses to support replication), each virus also possesses unique functional hotspots with virus-specific characteristics (Figure R19A). Accordingly, the differences between VTPs and non-VTPs across viruses enable the prediction of VTPs using host protein features alone.

To determine whether our model predicts genuine host-virus interactions or merely captures host susceptibility, we categorized VTPs into different types. First, VTPs were divided into multiple virus target proteins (MVTPs) and single virus target proteins (SVTPs) according to the number of targeting viruses. Second, VTPs were grouped into forward (pro-viral) and backward (anti-viral) types based on interaction directionality. Forward interactions mainly promote viral replication and are largely mediated by viral proteins, whereas backward interactions typically involve host immune-related proteins and are primarily mediated by host proteins. Because we only collected directionality annotations for HIV-1 VTPs, we used this virus as a case study. Analysis of prediction scores from 5-fold cross-validation showed that non-VTPs and VTPs targeted by other

viruses exhibited relatively low scores, whereas HIV-associated MVTPs and SVTPs achieved higher scores. Notably, HIV-1 SVTPs possessed higher scores than MVTPs targeted by multiple distinct viruses (including HIV-1). This implies that our model not only learns a common host susceptibility signature but also effectively identifies HIV-specific biological characteristics, thereby distinguishing true virus-specific host-virus interactions. For forward interactions mediated by viral proteins, the median prediction score was similar to that of non-VTPs, and only the upper quartile suggested that the model could capture relevant interaction patterns. In contrast, for backward interactions mediated by host proteins, their prediction scores were significantly higher than those of other categories (Figure R19B). This confirms that our model excels at predicting host protein-mediated interactions but has limitations in predicting those mediated by viral proteins. The revision is provided in Lines 208-217.

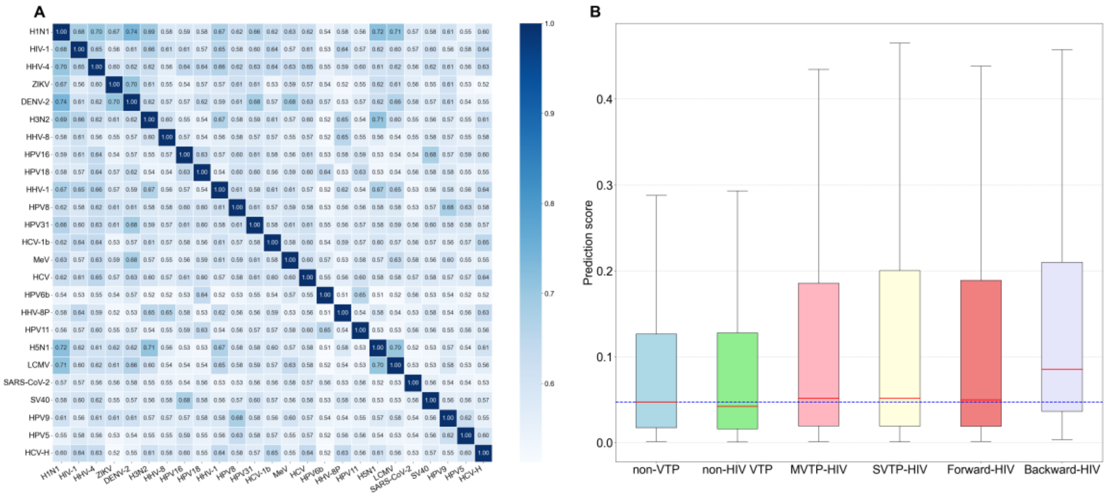

Figure R19. Functional comparison of VTPs for different viruses and prediction scores of HIV-1 target proteins. (A) Overlap rate of functional terms among different viruses. (B) Distribution of prediction scores for HIV-1 target proteins.

2. The model demonstrates weaker performance for single-virus target proteins (SVTPs). This limitation appears biologically inevitable rather than algorithmic, as SVTPs are often driven by virus-specific molecular determinants that cannot be inferred from shared host characteristics alone. This observation raises concerns about whether the framework can truly capture mechanistically specific interactions rather than broad host vulnerability patterns.

Response: Thank you for this suggestion. To investigate the performance discrepancy between SVTPs and MVTPs, we re-evaluated our model on these two groups separately using 5-fold cross-validation. Our results showed that the model achieved lower measures for SVTPs than for MVTPs (Figure R20). This is consistent with your opinion that SVTPs are mainly governed by virus specific molecular patterns, which are difficult to infer from host information alone. In contrast, MVTPs participate in

fundamental biological processes and possess more conserved functional and network properties. Moreover, the relatively larger data volume for these proteins allows the model to learn more robust and generalizable patterns. Especially, MVTPs represent promising targets for broad-spectrum antiviral drug development, the better performance of our model on this class highlights its practical value. Furthermore, the predictive performance for SVTPs outperforms the random baseline, demonstrating that our framework is capable of capturing biologically meaningful signals underlying these specific virus-host interactions. The revision is provided in Lines 382-386.

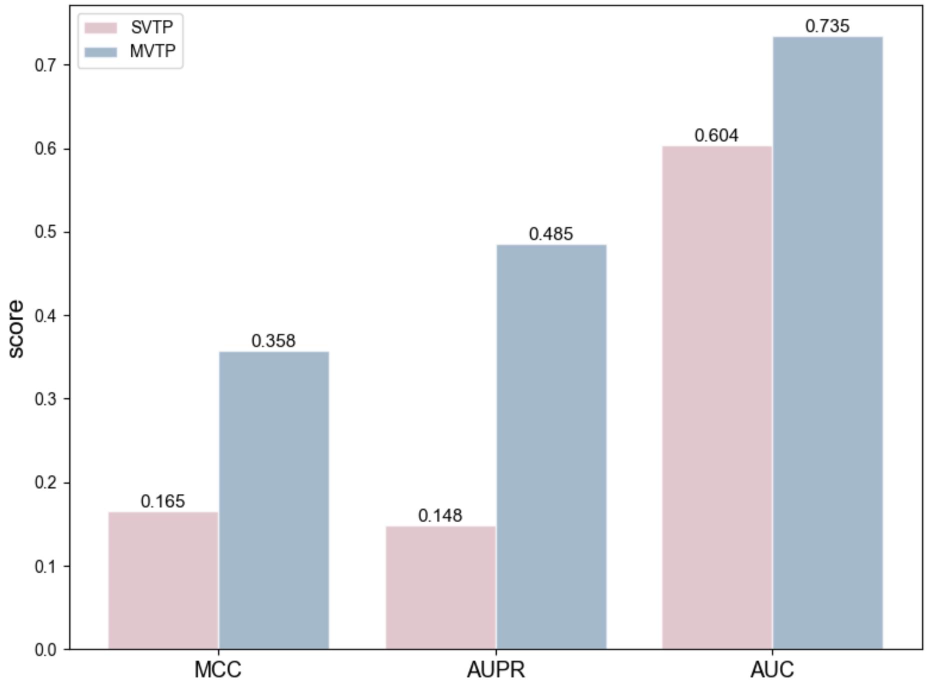

Figure R20. Performance comparison of our model on SVTPs and MVTPs

3. Different viruses frequently converge on the same signaling pathways while employing fundamentally different molecular strategies. For example, two viruses may both target the NF-kB pathway, yet one may directly bind NFKB1 whereas another modulates upstream adaptor proteins. Without incorporating viral protein information, it remains unclear whether the proposed model can differentiate these mechanistic scenarios or merely capture pathway-level correlations.

Response: Thank you for your insightful comment. To investigate whether our model could identify the target proteins of different viruses in the same pathway, we analyzed VTPs and non-VTPs within the pathways from the test set. For the NF-κB pathway, the analysis showed that the predicted scores for VTPs of HIV-1, HHV-4, and HPV16 were higher than those of non-VTPs, while the results of HHV-1 and HCV-1b showed the opposite trend (Figure R21A). This discrepancy may stem from the limited number of proteins covered by the NF-κB pathway in the test set (only 12 proteins), resulting in insufficient statistical power. We further extended our analysis to the cytokine-cytokine receptor interaction pathway (41 proteins), the immune pathway containing the largest

number of proteins in the test set (Figure R21B). Due to the scarcity of known VTPs in this pathway, the results remain inconclusive. To provide a more robust and convincing analysis, we retrained our model after excluding all NF-κB pathway-related proteins from the training set and re-evaluated the model on these proteins along with those from the test set (a total of 60 proteins). The result showed that VTPs had higher prediction score than non-VTPs, demonstrating that our model could effectively predict the VTPs of different viruses in the same pathway (Figure R21B). Regarding the direct and indirect interactions mentioned by the reviewer, such a mechanistic analysis may be difficult to perform given the interpretability of our current model and the limited data. We will continue to investigate this issue in the future. The revision is provided in Lines 208-217.

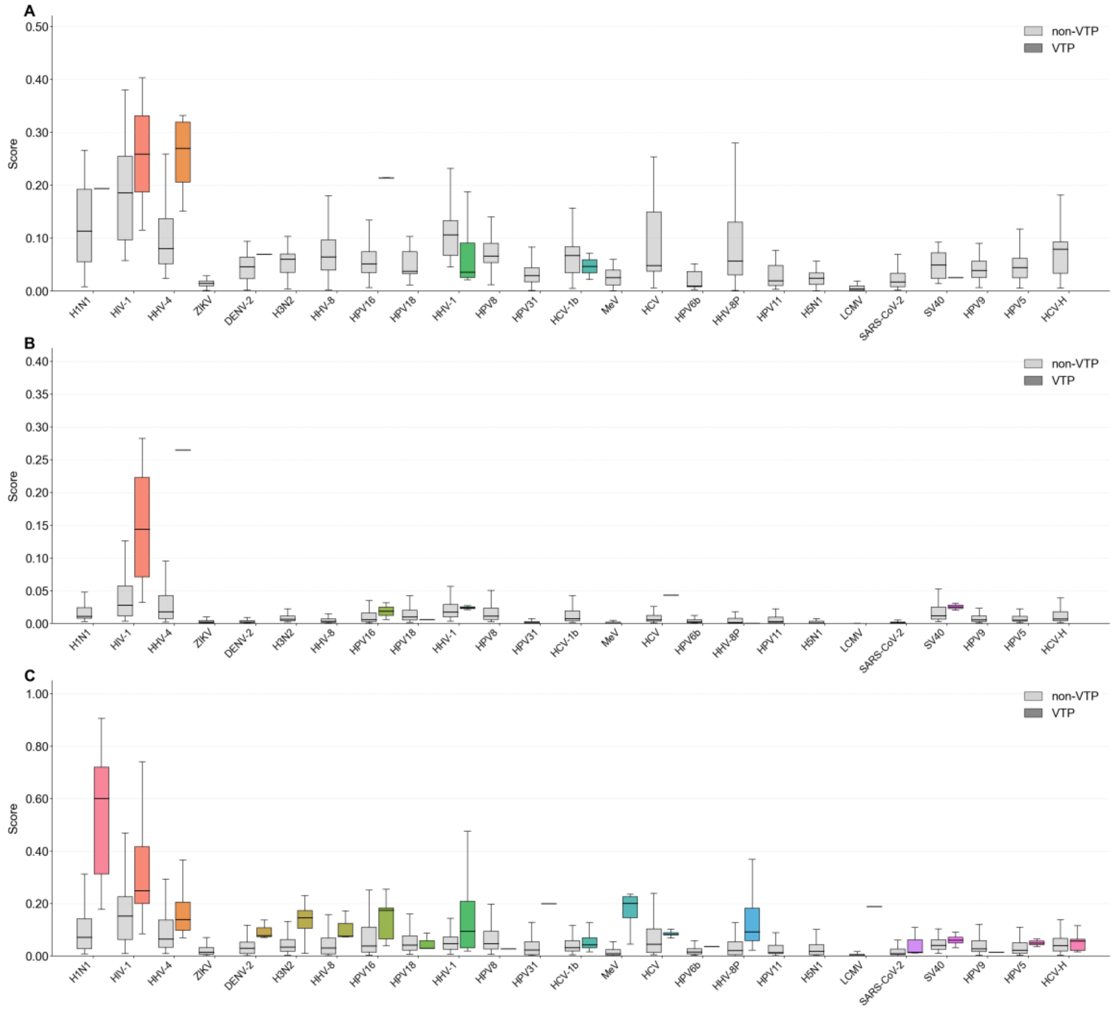

Figure R21. Comparison of prediction scores for VTPs and non-VTPs in the same pathway. (A) Prediction scores of proteins involved in the NF-κB pathway. (B) Prediction scores of proteins involved in the cytokine-cytokine receptor interaction pathway. (C) Prediction scores of proteins involved in the NF-κB pathway using the retrained model.

4. The construction of negative samples warrants careful reconsideration. Proteins lacking documented interactions are treated as non-VTPs; however, host–virus interaction databases remain highly incomplete. As a result, the negative set likely

contains undiscovered true targets, introducing label noise that may cause the model to learn patterns reflecting research intensity rather than biological reality. The authors should discuss this limitation more explicitly and consider alternative strategies such as positive–unlabeled learning or more conservative negative sampling.

Response: To evaluate the effect induced by the quality of non-VTPs, we adopted two strategies suggested by the reviewer to construct the training set. The first strategy utilizes the positive-unlabeled learning method with a spy technique. We randomly selected 15% of VTPs as ‘spy samples’ and combined them with non-VTPs to train an XGB classifier. We then set the lowest score covering 95% of the spy samples as the threshold, and considered non-VTPs with a score below this threshold as reliable negative samples. We retrained our model with this refined training set and evaluated its performance on test sets. The results showed that all metrics except recall decreased. This is likely because while the strategy successfully removes false negative samples, the selected non-VTPs differ markedly from VTPs in terms of their features. As a result, the model fails to adequately learn from ‘difficult negative samples’, and thus struggles to accurately predict non-VTPs that resemble genuine VTPs. For the second strategy, we performed functional enrichment analysis on VTPs and selected GO terms and KEGG pathways with a p-value < 0.05 and fold enrichment > 2 as more conserved functions for viral infection. Non-VTPs not annotated to these terms were then regarded as reliable non-VTPs. The results indicated that the MCC, recall, precision, and F1 measures increased, while the AUPR measure remained stable (Table R3). This could be attributed to the higher quality of negative samples and the reduction of label noise. Altogether, our analyses demonstrate that the initial non-VTP selection scheme reliably reflects the predictive power of our model. The revision is provided in Lines 427-433.

Table R3 Performance comparison of different negative sampling strategies

Negative sampling strategy	D_{virus}					
	MCC	AUPR	AUC	RC	F1	Pre
MultiVTP	0.347	0.375	0.839	0.433	0.370	0.390
PU_spy	0.323	0.362	0.830	0.558	0.336	0.314
Not in BP	0.372	0.376	0.840	0.482	0.390	0.408
Not in CC	0.373	0.375	0.840	0.507	0.390	0.395
Not in KEGG	0.376	0.375	0.841	0.482	0.393	0.421
Not in any conserved terms	0.372	0.371	0.838	0.469	0.390	0.417

5. Ablation experiments indicate that network topology contributes most strongly to

predictive performance. This raises a critical concern that the model may primarily function as a detector of high-centrality proteins rather than identifying mechanistically relevant targets. Hub proteins in PPI networks are well known to be overrepresented in interaction datasets due to observational bias. If predictions are largely driven by centrality signals, the model may be capturing dataset artifacts rather than biological principles. Re-evaluating performance under degree-controlled settings would help clarify this issue.

Response: Thank you for this comment. To determine whether the model detects merely high-centrality proteins or truly virus targets, we re-evaluated its performance under the degree-controlled settings. Specifically, we divided the proteins into 10 bins based on their degree measures in the PPI network and assessed the performance using cross-validation. Although performance decreased across the (10, 70) intervals, our model still attained a notable AUPR of 0.287 for the lowest-degree interval, suggesting that VTP prediction does not solely rely on centrality signals (Figure R22A). Furthermore, we conducted two additional ablation studies to ensure the model is not merely detecting topological features and to prevent inflated performance due to network proximity. First, we excluded all network-related components, including traditional topological properties, global/local topological attributes, and subgraphs. As a result, the model still retained robust performance, suggesting that its utility is not solely driven by these topological features. Second, we masked edges between the training and test samples to prevent information leakage and re-extracted all network topological features and subgraphs. The results showed that the model maintained stable performance, confirming that the predictive power of the model is not due to information leakage from neighboring proteins (Figure R22B). The revision is provided in Lines 243-245.

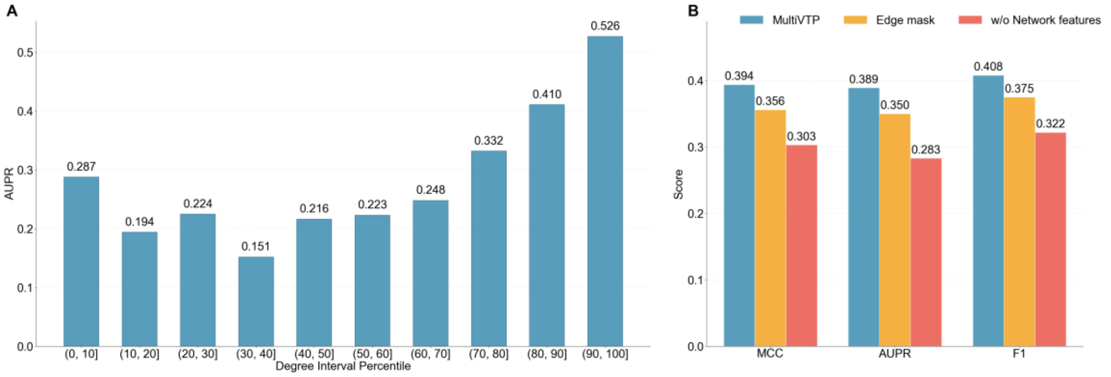

Figure R22. Validation of MultiVTP's network dependency. (A) Performance evaluation on proteins with different degree measures. (B) Ablation studies of network features.

6. Although the authors include few-shot experiments, viruses with extremely limited annotations (e.g., fewer than 20 validated targets) are not evaluated. This omission is non-trivial, as real-world deployment of such models is most critical precisely in scenarios involving emerging or poorly characterized viruses, where labeled data are inherently scarce. Consequently, the current experimental design may overestimate the practical utility of the framework. Moreover, the few-shot setting considered in this

study still assumes the availability of a relatively informative training signal. It remains unclear whether the model can maintain stability when supervision becomes extremely sparse or highly imbalanced. Without testing under more stringent low-data conditions, the claim of robustness appears insufficiently supported. More challenging evaluation strategies, such as leave-one-virus-out validation or ultra-low-shot settings, would better approximate realistic application scenarios and demonstrate true generalizability.

Response: Thank you for this suggestion. To assess the model's robustness under more stringent low-data conditions, we conducted two experiments as requested. First, we simulated scenarios with extremely scarce labeled data by progressively removing VTPs for each virus in the training set at a high ratio. Notably, when 99% of VTPs were removed, even for the virus with the largest number of targets (H1N1), fewer than 20 VTPs remained available for training. The model was then evaluated on the test set. All experiments were repeated five times to reduce fluctuations caused by random sampling under limited data. The results showed that our model maintained stable AUPR values even after removing 90% of known VTPs, while performance dropped sharply when 99% of VTPs were eliminated (Figure R23A). These results confirmed that the model remains effective under scarce labeled data conditions. Second, we performed leave-one-virus-out validation on five randomly selected viruses. Specifically, the model was pre-trained on a training set that excluded VTPs of the target virus. Because the pre-trained model lacked an output channel for the new virus, we introduced a virus-specific expert and prediction head, which were then fine-tuned using only 20 targets from that virus. The model was subsequently evaluated on the test set, with all experiments repeated five times. Compared with the original training scheme, this strategy yielded reduced but still effective performance across the five viruses, verifying the model's generalization ability to unseen viruses (Figure R23B). The revision is provided in Lines 329-332.

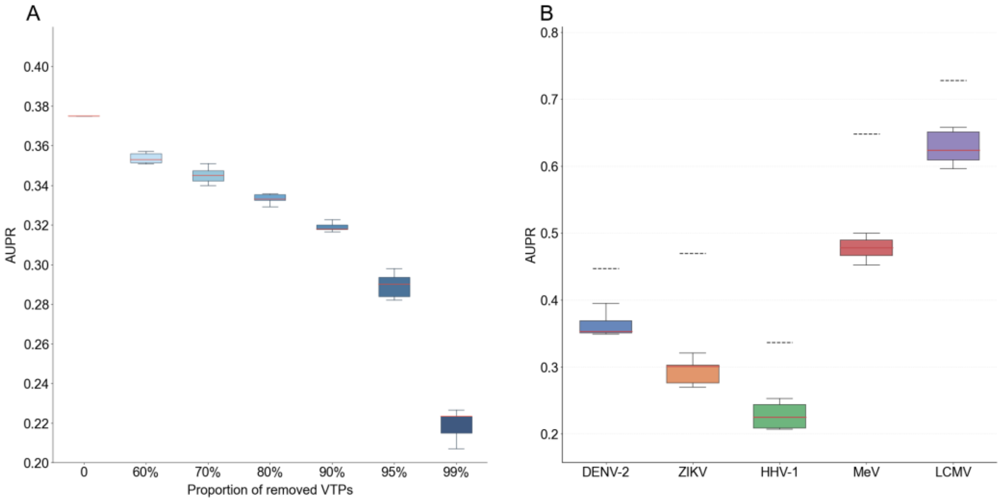

Figure R23. Robustness of MultiVTP with limited training data. (A) Performance of MultiVTP after removing different fractions of training VTPs. (B) Performance of leave-one-virus-out validation.

7. Potential data leakage represents another important concern. Proteins targeted by multiple viruses frequently share sequence similarity, evolutionary origin, or network proximity. If homologous or topologically related proteins appear across training and testing splits with correlated label vectors, the model may benefit from implicit information transfer, thereby inflating predictive performance. While the manuscript mentions sequence redundancy reduction, it remains unclear whether redundancy was sufficiently controlled at the label level across folds. Standard clustering thresholds may not fully eliminate functional similarity, particularly within large protein families. Consequently, the reported performance may partially reflect memorization rather than genuine predictive capability. Providing a quantitative assessment of cross-fold homology, functional similarity, or neighborhood overlap would substantially strengthen confidence in the evaluation.

Response: Thank you for this insightful suggestion. To analyze the impact of potential similarities on the performance, we quantified the similarity between the training and test sets across three dimensions: protein sequence, functional annotation, and network topology.

For sequence homology, we employed BLAST to conduct sequence similarity analysis for each fold during cross-validation. Specifically, we compared each protein in the validation set against all proteins in the corresponding training set, and used the highest sequence identity as the homologous measure. As a result, the distributions of sequence similarity were consistent across all folds (Figure R24A). Subsequently, we evaluated the performance on validation samples based on their similarity relative to training samples. This evaluation revealed that our model's effectiveness did not degrade as sequence identity decreased. Even in the lowest sequence identity range (0, 20), the MCC, AUPR, and F1-score all remained above 0.4 (Figure R24B). These results implied that the model's predictive ability was not dependent on homologous information, and that no overfitting risk existed at the sequence level.

For functional annotation, we used GOSemSim to quantify functional similarity. The highest functional similarity score was assigned to each protein in the validation set. The results showed that the distribution of functional similarity was consistent, with values centered at approximately 0.3 (Figure R24C). Although the model's performance fluctuated with decreasing functional similarity, the MCC and F1-score remained relatively stable even in the lowest functional similarity range (0, 0.3) (Figure R24D). This indicates that the model maintained effective predictive performance for samples with functional annotations clearly distinct from those in the training set, demonstrating that it truly learned the biological principles underlying VTPs.

For network proximity, topological similarity was quantified using the maximum neighborhood overlap rate between each validation protein and all training proteins. The low neighborhood overlap (around 0.1) indicates limited topological resemblance between the training and test sets. Threshold-based evaluations revealed that the model maintained strong performance for proteins with an overlap rate above 0.2, while performance dropped noticeably for those below 0.1. The inferior performance may be attributed to their inherent biological characteristics. In the highly connected host PPI

network, these proteins are typically peripheral or isolated nodes that rarely participate in core functional pathways. Viral targeting of such proteins may be highly specific yet idiosyncratic, thus lacking generalizable patterns. Besides, these proteins often have sparse functional and interaction annotations in public databases, further increasing prediction difficulty. To address the concern that the observed performance may be overestimated due to implicit information leakage in the network, we performed an edge-masking ablation study as detailed in Response 5. After blocking information propagation, the model still retained robust performance. The revision is provided in Lines 228-235.

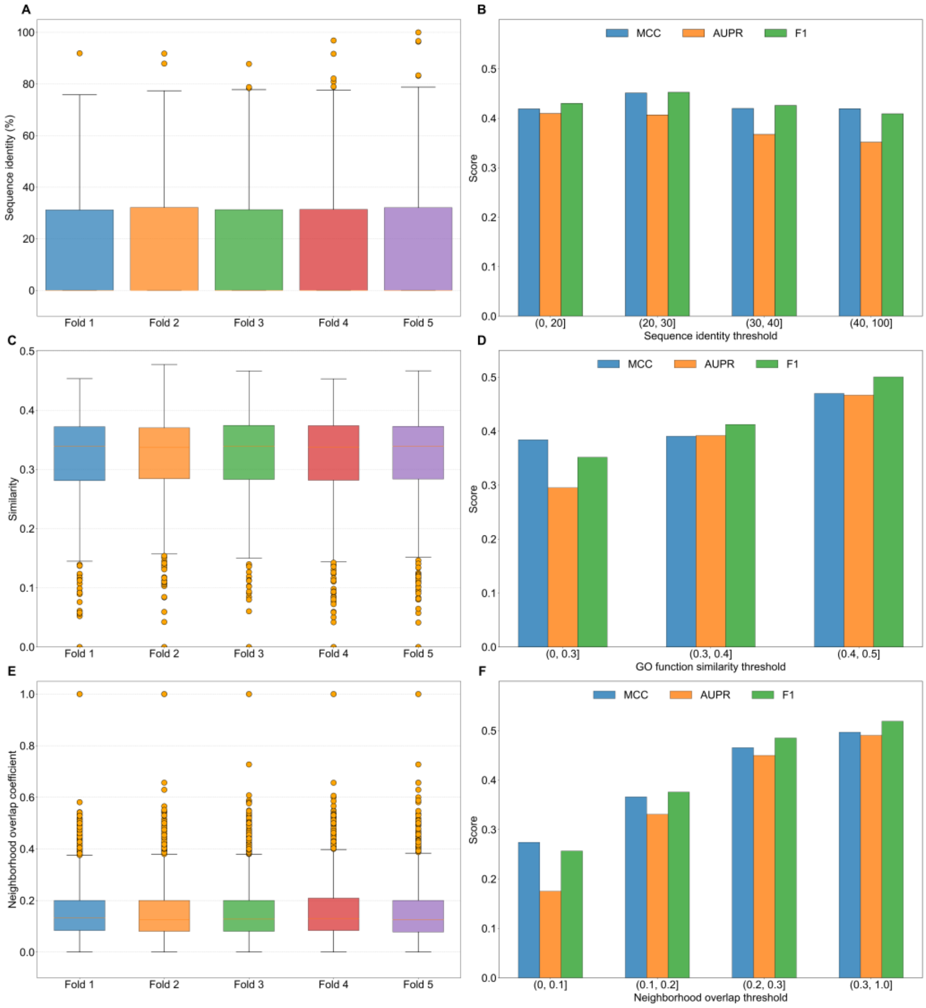

Figure R24. Cross-fold similarity and model performance. (A) Sequence identity distribution across all folds. (B) Performance on cross-validation sets under different sequence identity thresholds. (C) Functional similarity distribution across all folds. (D) Performance on cross-validation sets under different functional similarity thresholds. (E) Topological similarity distribution across all folds. (F) Performance on cross-validation sets under different topological similarity thresholds.

8. The exclusion of viruses with fewer than 200 annotated targets raises concerns about potential systematic bias. Well-studied viruses typically possess richer interaction data, more complete annotations, and stronger network signals, all of which may inadvertently simplify the prediction task. As a result, the model may become optimized

for data-rich pathogens while exhibiting reduced reliability for understudied or newly emerging viruses. This filtering strategy may distort the empirical distribution of host–virus interactions and limit the ecological validity of the framework. In practice, computational tools are often most valuable precisely where experimental knowledge is limited. A model that performs well only on heavily characterized viruses may therefore have restricted translational relevance.

Response: Thank you for this comment. In the initial training procedure, we included viruses with at least 200 VTPs to avoid the seesaw phenomenon and negative transfer caused by severe label imbalance across tasks in multi-task learning, ensuring that the model can learn generalizable principles underlying host-virus interactions. For newly emerging viruses, we extended the original model by incorporating virus-specific expert modules and output channels, followed by transfer learning. In leave-one-virus-out validation, the performance of our model remained stable across five randomly selected viruses (see Response 6). To further address the issue of viruses with limited available data, we constructed a more challenging few-shot dataset consisting of 15 novel viruses (each with 5-17 VTPs), with none of these samples appearing in the training set (Figure R25A). Under this setting, models trained from scratch suffered from severe performance collapse (AUPR < 0.1). In contrast, our fine-tuning strategy achieved clearly better performance, with AUPR values ranging from 0.1 to 0.7 for most viruses. Relatively poor performance was observed for the remaining viruses (e.g., HPV3, HPV16, H7N7, and H9N2), likely because some of their targets are SVTPs that lack cross-virus generalizable patterns, which remains a challenge for the current model (Figure R25B). Collectively, our model is suitable for understudied or newly emerging viruses. The revision is provided in Lines 329-332.

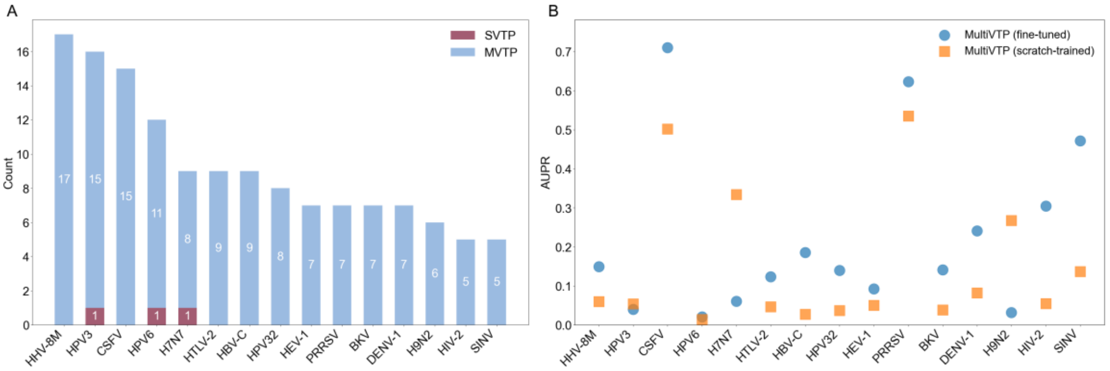

Figure R25. Evaluation of MultiVTP on understudied viruses. (A) Distribution of VTP counts across viruses in the few-shot dataset. (B) Performance of our model on understudied viruses.

9. Regarding future work, please discuss the possibility of applying advanced learning techniques (10.1109/TCBBIO.2025.3610881 and 10.1109/JBHI.2025.3600045) for improved performance of the proposed model.

Response: Thank you. DeepHIV (10.1109/TCBBIO.2025.3610881) provides valuable inspiration for improving MultiVTP. In our current model, sequence features are encoded using ESM2 embeddings followed by mean pooling, but this process lacks

positional information. Given that the viral binding capability of VTPs depends on critical residues and functional domains, we can enhance the encoder by incorporating an attention mechanism. Such an improvement is expected to not only optimize sequence representation but also enhance model interpretability. Additionally, the imbalanced distribution of VTPs across different viruses may cause the model to overlook signals from understudied viruses. To miigate this issue, we plan to introduce a dynamically weighted loss function that assigns higher penalties to viruses with fewer VTPs, thereby strengthening the model's ability to learn from limited samples. Inspired by the multi-view contrastive learning and multi-modal modeling of MCL-DDI (10.1109/JBHI.2025.3600045), we propose three potential optimizations for MultiVTP. First, we introduce contrastive learning within each modality to minimize the distance between VTPs of the same virus while maximizing their separation from non-VTPs. Second, we replace the original feature concatenation with attention-gated fusion, enabling the model to adaptively integrate multimodal information. Third, we extend the original subgraphs by incorporating additional edge types such as regulatory and modification relationships. This yields a heterogeneous graph that can be processed using relational graph neural networks to improve graph representation learning. The revision is provided in Lines 440-453.

Response to reviewer 5

1. Dataset construction and potential information leakage

Because features include network topology, GO similarity graphs, and pretrained embeddings, proteins closely related to training examples may remain strongly coupled to them through the PPI network or functional similarity structure.

The authors should clarify whether homologous proteins or highly connected network neighbors are separated across splits, and whether alternative splitting strategies were explored. Without this clarification, performance estimates may be optimistic.

Response: Thank you for this insightful suggestion. To analyze the impact of potential similarities on the performance, we quantified the similarity between the training and test sets across three dimensions: protein sequence, functional annotation, and network topology.

For sequence homology, we employed BLAST to conduct sequence similarity analysis for each fold during cross-validation. Specifically, we compared each protein in the validation set against all proteins in the corresponding training set, and used the highest sequence identity as the homologous measure. As a result, the distributions of sequence similarity were consistent across all folds (Figure R26A). Subsequently, we evaluated the performance on validation samples based on their similarity relative to training samples. This evaluation revealed that our model's effectiveness did not degrade as sequence identity decreased. Even in the lowest sequence identity range (0, 20), the MCC, AUPR, and F1-score all remained above 0.4 (Figure R26B). These results implied that the model's predictive ability was not dependent on homologous information, and that no overfitting risk existed at the sequence level.

For functional annotation, we used GOSemSim to quantify functional similarity. The highest functional similarity score was assigned to each protein in the validation set. The results showed that the distribution of functional similarity was consistent, with values centered at approximately 0.3 (Figure R26C). Although the model's performance fluctuated with decreasing functional similarity, the MCC and F1-score remained relatively stable even in the lowest functional similarity range (0, 0.3) (Figure R26D). This indicates that the model maintained effective predictive performance for samples with functional annotations clearly distinct from those in the training set, demonstrating that it truly learned the biological principles underlying VTPs.

For network proximity, topological similarity was quantified using the maximum neighborhood overlap rate between each validation protein and all training proteins. The low neighborhood overlap (around 0.1) indicates limited topological resemblance between the training and test sets (Figure R26E). Threshold-based evaluations revealed that the model maintained strong performance for proteins with an overlap rate above 0.2, while performance dropped noticeably for those below 0.1 (Figure R26F). The inferior performance may be attributed to their inherent biological characteristics. In the highly connected host PPI network, these proteins are typically peripheral or isolated nodes that rarely participate in core functional pathways. Viral targeting of such proteins may be highly specific yet idiosyncratic, thus lacking generalizable

patterns. Besides, these proteins often have sparse functional and interaction annotations in public databases, further increasing prediction difficulty.

Furthermore, we conducted two additional ablation studies to ensure the model is not merely detecting topological features and to prevent inflated performance due to network proximity. First, we excluded all network-related components, including traditional topological properties, global/local topological attributes, and subgraphs. As a result, the model still retained robust performance, suggesting that its utility is not solely driven by these topological features. Second, we masked edges between the training and test samples to prevent information leakage and re-extracted all network topological features and subgraphs. The results showed that the model maintained stable performance, confirming that the predictive power of the model is not due to information leakage from neighboring proteins (Figure R27). The revision is provided in Lines 228-235 and 243-245.

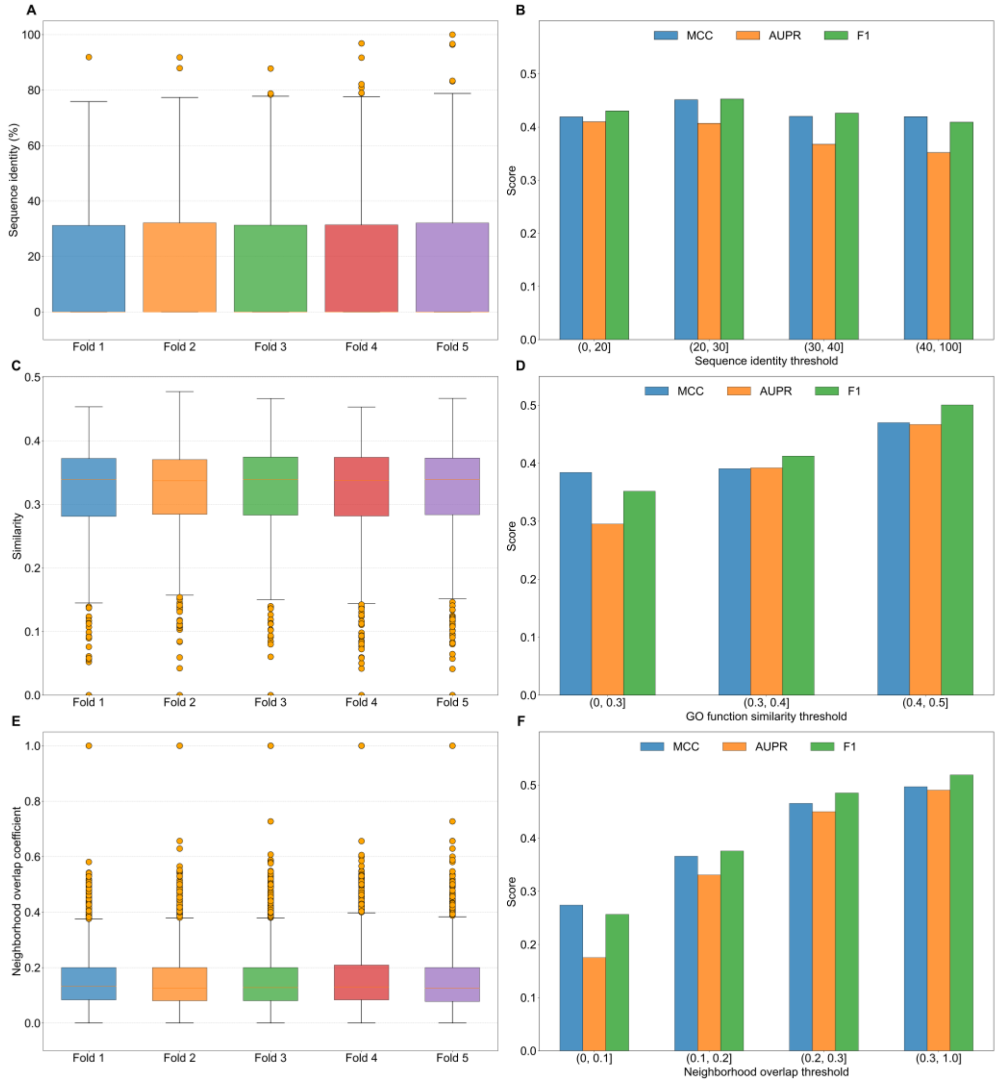

Figure R26. Cross-fold similarity and model performance. (A) Sequence identity distribution across all folds. (B) Performance on cross-validation sets under different sequence identity thresholds. (C) Functional similarity distribution across all folds. (D) Performance on cross-validation sets under different functional similarity thresholds. (E) Topological similarity distribution across all folds. (F) Performance on cross-validation sets under different topological similarity thresholds.

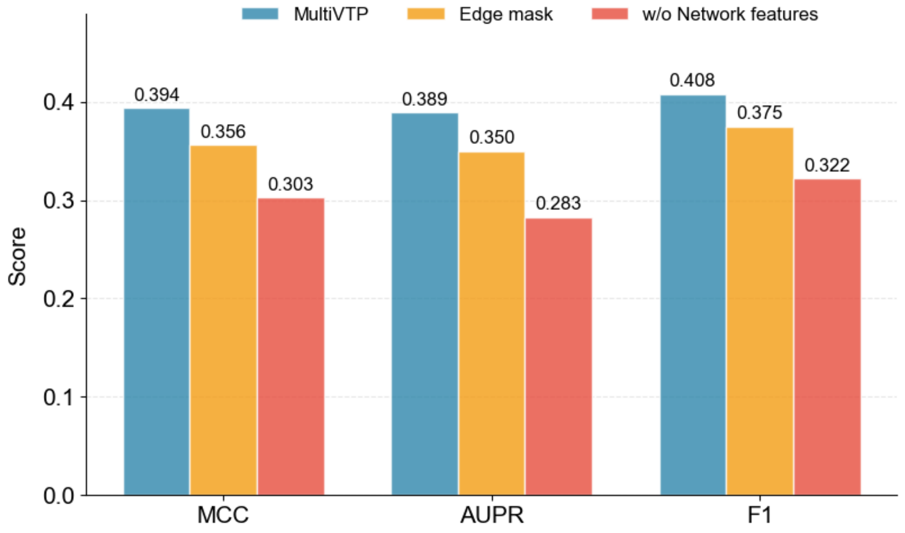

Figure R27. Ablation studies of network information in our model

2. Benchmarking against recent methods

Comparisons focus largely on classical machine learning models and HIVPRE. Given recent advances in protein language models and deep-learning approaches for host–virus interaction prediction, additional contextualization would strengthen the manuscript. Either stronger comparisons or discussion of limitations in performing such comparisons would be helpful.

Response: Thank you for this valuable suggestion. We selected two representative deep learning models as supplementary baselines, namely D-SCRIPT and HBFormer. D-SCRIPT is a sequence-based PPI prediction model with strong generalization in cross-species interaction prediction, whereas HBFormer uses a hybrid attention transformer combined with multimodal features for host-virus PPI prediction. Because these models were originally designed for binary PPI predictions, we adjusted the prediction strategy to ensure a fair comparison. Specifically, each host protein was paired with all viral proteins in the HVIDB database, and the highest score among all PPI predictions was used as the prediction score for the host protein. Considering the high computational cost of this pairing strategy, D-SCRIPT was evaluated on all 25 viruses due to its higher efficiency, whereas HBFormer was only tested on the five viruses with the highest VTP counts owing to its lower efficiency. The results suggested that MultiVTP remarkably outperformed the baseline models in terms of AUPR across all viruses (Figure R28). This may be because selecting the highest PPI score leads the model to more easily classify host proteins as false positives. Therefore, traditional binary PPI classification methods are not suitable for VTP prediction. The revision is provided in Lines 289-292.

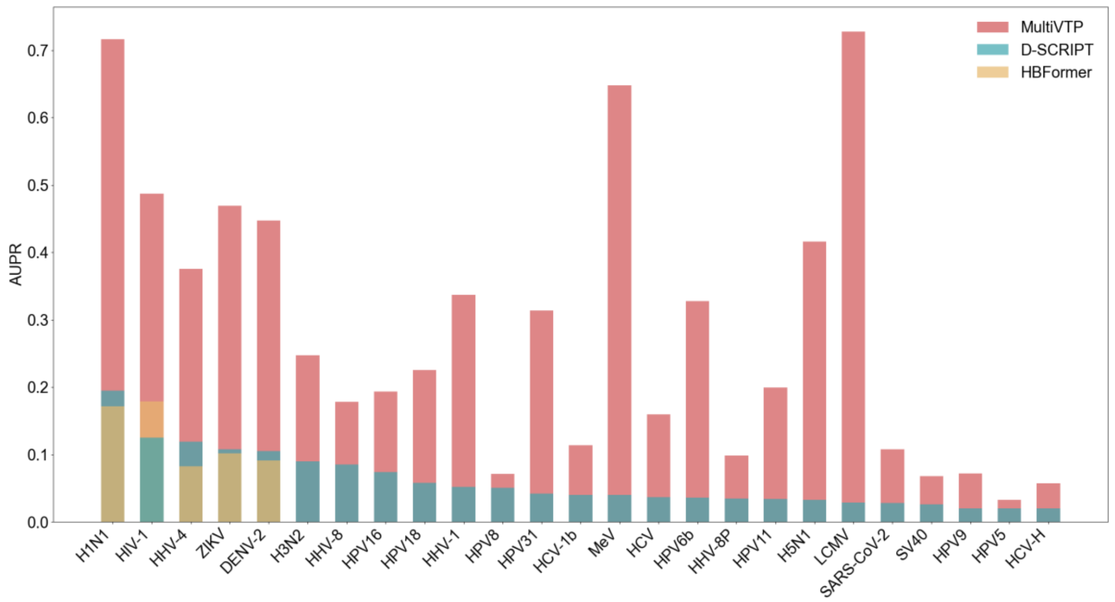

Figure R28. Performance comparison of MultiVTP and PPI prediction models for VTP identification.

3. Model over(?)-complexity

The model combines:

• Traditional handcrafted biological features
• ESM2 sequence embeddings
• node2vec global topology embeddings
• Shortest-path distance encodings
• Graphormer-based attention
• A Progressive Layered Extraction (PLE) multilabel module

Individually, each component is reasonable. However, taken together, the architecture becomes quite layered and potentially redundant. Although the ablation studies show that removing certain components degrades performance, the reported improvements over strong baselines are moderate. This raises the question of whether a simpler architecture (e.g., ESM2 + node2vec + shallow GCN or shared multilabel MLP head) might achieve comparable performance with reduced complexity. Such a comparison would help clarify whether the proposed design is necessary.

Response: Thank you for your comment. To evaluate whether simpler architectures can achieve comparable performance, we conducted three additional ablation studies using only node2vec and ESM2 embeddings as features: (1) replacing the PLE module with a shared MLP head; (2) replacing the Graphormer with a 2-layer GCN; and (3) applying both modifications simultaneously. The results demonstrated that each component in the original model contributes to the overall performance, and a substantial performance drop occurs when these key components are replaced (Figure R29). The revision is provided in Lines 249-251.

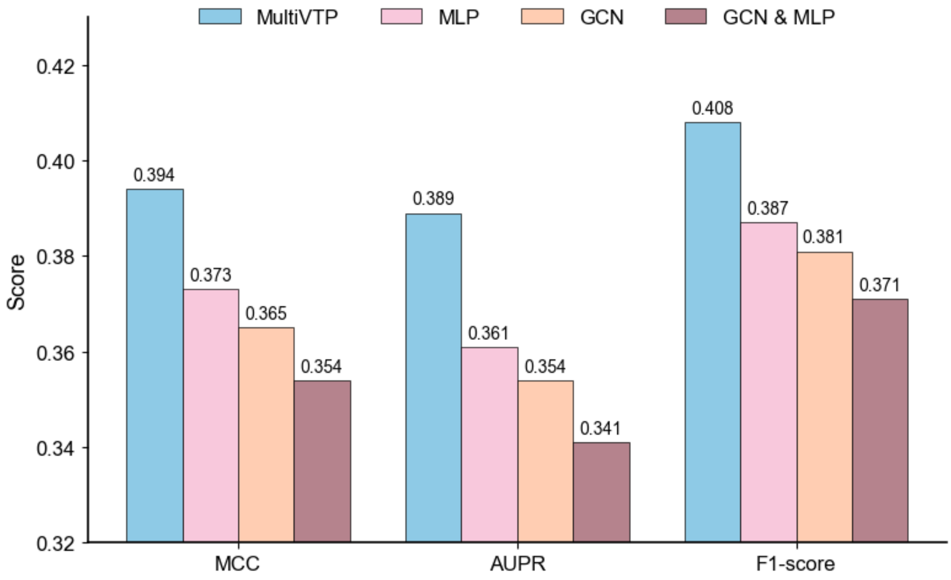

Figure R29. Performance comparison between MultiVTP and simpler architectures.
